# The Genetic Architecture for Phenotypic Plasticity of the Rice Grain Ionome

**DOI:** 10.3389/fpls.2020.00012

**Published:** 2020-02-25

**Authors:** Yongjun Tan, Jieqiang Zhou, Jiurong Wang, Liang Sun

**Affiliations:** ^1^ Key Laboratory of Agro-Ecological Processes in Subtropical Region, Institute of Subtropical Agriculture, Chinese Academy of Sciences, Changsha, China; ^2^ University of Chinese Academy of Science, Beijing, China; ^3^ College of Agronomy, Hunan Agricultural University, Changsha, China

**Keywords:** rice, grain ionome, phenotypic plasticity, genetic architecture, phenotypic divergence

## Abstract

The ionome of the rice grain is crucial for the health of populations that consume rice as a staple food. However, the contribution of phenotypic plasticity to the variation of rice grain ionome and the genetic architecture of phenotypic plasticity are poorly understood. In this study, we investigated the rice grain ionome of a rice diversity panel in up to eight environments. A considerable proportion of phenotypic variance can be attributed to phenotypic plasticity. Then, phenotypic plasticity and mean phenotype were quantified using Bayesian Finlay-Wilkinson regression, and a significant correlation between them was observed. However, the genetic architecture of mean phenotype was distinct from that of phenotypic plasticity. Also, the correlation between them was mainly attributed to the phenotypic divergence between rice subspecies. Furthermore, the results of whole-genome regression analysis showed that the genetic loci related to phenotypic plasticity can explain a considerable proportion of the phenotypic variance in some environments, especially for Cd, Cu, Mn, and Zn. Our study not only sheds light on the genetic architecture of phenotypic plasticity of the rice grain ionome but also suggests that the genetic loci which related to phenotypic plasticity are valuable in rice grain ionome improvement breeding.

## Introduction

The ability of one genotype to produce multiple phenotypes in response to environmental change has been termed “phenotypic plasticity” (Bradshaw, 1965; Via and Lande, 1985; Des Marais et al., 2013; Kusmec et al., 2018). Variation in phenotypic plasticity in a diversity panel defines the genotype-by-environment interaction (G×E) (Bradshaw, 1965; Des Marais et al., 2013). The prominence of phenotypic plasticity in crops depends on traits and environmental scenarios. Lower plasticity in disease resistance is crucial to broadly-adaptability cultivars, while phenotypic plasticity can be harnessed to improve the cultivars' yield performance in determined environmental scenarios with an adequate supply of water and fertilizer. For traits show G×E, incorporating G×E in the genomic prediction can boost its accuracy, especially in field experiments performed in a wide range of environmental scenarios (Lopez-Cruz et al., 2015; Malosetti et al., 2016; Millet et al., 2019). The prerequisite for utilizing phenotypic plasticity in breeding practice is investigating the effect of phenotypic plasticity on phenotypic variance and dissecting the genetic architecture for phenotypic plasticity.

Phenotypic plasticity has been investigated in several crop species such as maize, wheat, rye, oat, barley, and rice for different traits including morphology, yield, and resistance to abiotic stress (Lacaze et al., 2009; Sasaki et al., 2015; Wang et al., 2015; Kikuchi et al., 2017; Kusmec et al., 2017; Parent et al., 2017; Rispail et al., 2018). The variations in phenotypic plasticity in these traits contributed substantially to the total variance of the phenotypes that were measured in multiple environments (Millet et al., 2016; Gage et al., 2017; Parent et al., 2017). For example, Gage et al. (2017) showed that phenotypic plasticity in 858 unique maize hybrids explained a considerable proportion of the phenotypic variance (between 1% and 6% of the total variance) for 11 morphological and agronomic traits which were measured in 21 environments. Similar results were also observed in a rice panel for yield in response to planting density (Kikuchi et al., 2017). Furthermore, phenotypic plasticity is under genetic control (Huang et al., 2016a; Gage et al., 2017), and numerous genetic loci accounting for phenotypic plasticity have been identified through genome-wide association studies (GWAS) (Sasaki et al., 2015; Millet et al., 2016; Gage et al., 2017; Kikuchi et al., 2017; Kusmec et al., 2017) or quantitative trait locus (QTL) mapping (Ungerer et al., 2003; Lacaze et al., 2009; Welcker et al., 2011). The candidate genes for mean phenotype and phenotypic plasticity are distinct in maize (Kusmec et al., 2017). In addition, the genetic loci related to phenotypic plasticity of some traits had been selected in the breeding history of maize (Gage et al., 2017).

The total mineral element content, known as the ionome, in the rice grain is crucial for the health of nearly half of the world's human population who consume rice as their staple food (Parengam et al., 2010). Firstly, the planting environment can significantly affect the rice grain ionome (Zhang et al., 2014; Yang et al., 2018). Rice accessions grown under flooded can accumulate more As but less Cd in rice grain than those grown under unflooded, because the flooding of rice paddies can decrease the bioavailability of Cd but increase the bioavailability of As in the soil (Norton et al., 2012; Zhang et al., 2014; Honma et al., 2016). Secondly, the rice grain ionome was also controlled by the genotype, and a large number of studies have been performed to dissect its genetic basis. Dozens of genes were identified in functional genomic studies, most of them were found to encode transporters, such as *OsHMA2* for Cd and Zn (Satoh-Nagasawa et al., 2012), *OsNRAMP5* for Cd and Mn (Ishikawa et al., 2012; Sasaki et al., 2012), and *Lsi1/2* for As and Si (Ma et al., 2007; Ma et al., 2008). Moreover, QTL mapping (Lu et al., 2008; Kashiwagi et al., 2009; Ishikawa et al., 2010; Norton et al., 2010; Abe et al., 2011; Zhang et al., 2014; Yu et al., 2015; Hu et al., 2016; Descalsota et al., 2018) and GWAS (Norton et al., 2014; Huang et al., 2015; Nawaz et al., 2015; Yang et al., 2018; Zhang et al., 2018) were also used to identify genetic loci which are related to the accumulation of mineral elements in the rice grain. Several QTLs have been cloned, such as *HMA3* (Ueno et al., 2010) and *OsCd1* (Yan et al., 2019) for Cd, *OsHMA4* (Huang et al., 2016b) for Cu. Finally, the interaction between the genotype and environment was also observed, and dozens of environmental-specific genetic loci were identified. For example, a different number of QTLs for the rice grain ionome were identified in flooded (N = 92) and non-flooded (N = 47) environments using the same introgression lines (ILs), while only three QTLs were detected repeatedly under these two conditions (Zhang et al., 2014). In a GWAS performed by Yang et al. (2018), only two of 53 significantly associated loci (SALs) could be detected repeatedly in two environmental conditions which varied for soil pH and elemental concentration. Xu et al. (2015) identified significant G×E effects for some identified QTLs using multiple environments test (MET). The identification of these environmental-specific genetic loci indicates that the interaction between genotype and environment was also under genetic control. However, the bi-parental populations used in the majority of these studies limit the identification of abundant genetic loci in all cultivars, and the limited number of planting environments cannot represent the diversity of environmental scenarios encountered during field production. Besides, the genetic relationship between mean phenotype and phenotypic plasticity of rice grain ionome was not investigated in these studies. Therefore, we conducted a systematic exploration of phenotypic plasticity of the rice grain ionome in a large and diverse population to address the following questions: (1) how much effect does phenotypic plasticity have on the phenotypic variance of each element in field environments? (2) What is the genetic architecture for mean phenotype and phenotypic plasticity, and are they correlated? And (3) can genetic loci which related to phenotypic plasticity be used in molecular breeding to optimize the elemental concentrations in rice grain?

To understand the role of phenotypic plasticity in the rice grain ionome, the concentrations of 16 elements in rice grain of a rice diversity panel were measured in four to eight environments. The response of each accession to the macro-environment and micro-environment was measured as linear plasticity and non-linear plasticity, respectively. The relationships between mean phenotype and two plasticity measures were investigated using the Pearson correlation coefficient (*r*) and the genetic correlation coefficient (*r_g_*). With significantly associated loci (SALs) identified by GWAS, the genetic architectures of the three phenotypic measures and the relationship among them were further investigated. In addition, the effect of phenotypic plasticity-related SALs was also estimated in each environment. Our study paves the way toward utilizing phenotypic plasticity of the rice grain ionome in diverse environments and will benefit the balance of the nutritional elements for human health.

## Materials and Methods

### Rice Diversity Panel and Determination of the Rice Grain Ionome

A rice diversity panel that contains 294 *indica*, 239 *japonica*, 20 *AUS*, and 22 *admix* accessions was used in this study (Mao et al., 2019; Tan et al., 2019). The genotyping of the entire panel was accomplished by a whole-genome resequencing (WGRS) strategy with a mean genomic coverage of 5.4× per accession. The genotyping procedure was described in our previous study (Mao et al., 2019; Tan et al., 2019). In total, a set of 6,493,721 SNPs and 833,968 Indels (length <6 bp) with minor allele counts (MAC) >5 were obtained.

The concentration of 16 elements in the rice grain of the diversity panel was measured in four to eight environments with a total of 20 replicates. A total of 5 to 13 soil samples (0–15 cm depth) were collected after harvest in each environment for analyzing the total elemental concentration and pH. These eight environments varied with respect to soil pH (from 5.2 to 7.8) and total element concentration; all of this information including sowing dates are detailed in **Table S1**. In order to minimize the spatial variation of soil properties, accessions of each replicate were planted in a nearly square field with one row (eight plants) per accession. The arrangement of all accessions in each replicate followed a randomized complete block design with a spacing of 17 cm between plants and a distance of 20 cm between rows in each field. All accessions growing in the same environment followed the same water regime (flooded or unflooded) during the period from the flowering of the first accession to the harvest of the last accession. The unflooded field was flush irrigated about 6 h when needed to prevent water stress. The rice grains from four plants in the middle of each row were harvested and air-dried. Then grains were dehusked with a modified rice huller, in which the roller was made from polyurethane instead of rubber to prevent metal contamination. The digestion of the brown rice (~0.25 g) was performed in Pyrex tubes with 5 mL nitric acid at 110℃ for 12 h (Lahner et al., 2003; Huang et al., 2016b). A total of 16 elements (As, Ca, Cd, Cr, Cu, Fe, K, Mg, Mn, Na, Ni, P, Pb, S, Se, and Zn) were quantified using ICP-AES (Agilent 720) or ICP-MS (Agilent 7900). The concentrations of 10 micro-elements (As, Cd, Cr, Cu, Fe, Mn, Ni, Pb, Se, and Zn) were used in another study which focused on dissecting the genetic relationships among the trace minerals in the rice grain (Tan et al., 2019). For each element, accessions that were measured in less than three environments were not included in the following analysis.

### Phenotype Analysis

The concentration of elements measured in each environment was illustrated using the R (version 3.5.2, https://www.r-project.org/) package ggplot2 (Wickham, 2016). Because the experimental design was unbalanced, the proportion of phenotypic variance for each element contributed by genotype, environment, and genotype-by-environment interaction was estimated by the linear mixed model. The calculation was performed in the R package lmer4 (Bates, 2014).

Phenotypic plasticity of each element was estimated in the R package FW by fitting phenotypes to Bayesian Finlay-Wilkinson Regression (Bayesian-FWR) (Finlay and Wilkinson, 1963; Lian and De Los Campos, 2015). The Bayesian-FWR implemented this equation: *y* *_ij_ = μ + g_i_ + (1+b_i_)h_j_ + ϵ_ij_*, on each element, where *y_ij_* is the element concentration of the *i*th accession measured in the *j*th environment, *g_i_* is the main genetic effect of the *i*th accession, *h_j_* is the mean effect of the *j*th environment, *(1+b_i_)* is the estimated slope of the *i*th accession, and *ϵ_ij_* is the residual error. The value of *g_i_* was obtained as the estimated mean phenotype value of the *i*th accession, and the value of *(1+b_i_)* was obtained as the linear response of the *i*th accession to macro-environments, or linear plasticity. The log-transformed variance of *ϵ_ij_* for each accession was obtained as the non-linear response to micro-environments, or non-linear plasticity (Kusmec et al., 2017).

The skewness (*g_1_*) and kurtosis (*g_2_*) of the three phenotypic measures (mean phenotype, linear plasticity, and non-linear plasticity) were calculated using the R package EnvStats (Craigmile, 2016). The overall performances of the different rice subspecies or subgroups for the three phenotypic measures were compared using the Kruskal-Wallis test in the R function “kruskal.test.” The Pearson correlation coefficient (*r*) among the three phenotypic measures for each element was calculated using the R function “cor.test.” The genetic correlation coefficient (*r_g_*) was estimated in the R function “mmer” from the package sommar, and the Kinship (K) used in this step was calculated from a set of 57,388 LD pruned SNPs. The same Kinship was also obtained in the R package heritability to calculate the marker-based heritability (*h^2^*) of each phenotypic measure. The phenotypic divergence (*P_ST_*) of each phenotypic measure between *indica* and *japonica* subspecies was calculated in the R package Pstat. All other data manipulation and illustrations were performed in R.

### Genome-Wide Association Study

In order to identify the genetic loci that account for the three phenotypic measures, a genome-wide association study (GWAS) was performed in the entire diversity panel (575 accessions), the *indica* subspecies (294 accessions) and *japonica* subspecies (239 accessions) by the FarmCPU (Liu et al., 2016) model which was implemented in the R package MVP (https://github.com/XiaoleiLiuBio/rMVP). The first three principal components (PCs), which were calculated using all SNPs in EIGENSTART (Price et al., 2006) (version 4.2), were obtained in the FarmCPU model to control the population structure. Because the majority of phenotypic measures were non-normal distribution, individuals with extreme values (departure from the mean larger than three times of standard deviation) were discarded; then a Box-Cox transformation was performed to minimize the departure of data from the assumption of the GWAS model. We observed that the thresholds calculated based on Bonferroni correlation at 0.05 significant level were more stringent than the thresholds estimated by permutation test at 0.05 significant level but close to thresholds estimated at 0.01 significant level. In order to guarantee type I error below 5%, the thresholds of the GWAS in three panels were defined based on the Bonferroni correction at the 0.05 significance level, which were defined as 1.8E-8 (0.05/2719301), 2.7E-8(0.05/1819762), and 5.5E-8(0.05/847835) for the whole panel, *indica*, and *japonica* subspecies, respectively. Variants with *P* values exceeding the threshold were declared to be significantly associated loci (SALs). The SALs for the three phenotypic measures were denoted as either mSALs (SALs for mean phenotype), lSALs (SALs for linear plasticity), or nlSALs (SALs for non-linear plasticity). Manhattan plots and quantile-quantile plots of the GWAS results were produced in the R package qqman (Turner, 2014) with few modifications to the color, shape, and size of the SALs.

### Identification of Overlap Between SALs

The overlap between mSAL, lSAL, and nlSAL of the same element was identified based on their confidence intervals. Because the FarmCPU model was adopted to conduct the GWAS, the significant P value was not expected for the variants adjacent to the peak variant. The confidence interval of each SAL was estimated through an LD-(linkage disequilibrium) based procedure: First, the LD (measured as *r^2^*) between the SAL and the flanking variable loci was calculated with Plink (Purcell et al., 2007); Then the confidence interval of each SAL was identified based on the criteria of *r^2^* > 0.6.

A permutation test with 10,000 resamplings was performed to estimate the probability of random overlap between the mSALs, lSALs, and nlSALs for the same element. In each permutation, a set of putative loci were randomly selected based on the number of SALs for this element. The null-distribution of the overlap between the three types of SAL was estimated from these 10,000 permutations. The *P* values for each kind of overlap were estimated based on the corresponding null-distributions. All analyses and the illustrations were performed in R.

### Estimation of the Phenotypic Variance Explained by Three Types of SALs

The phenotypic variances explained by three types of SAL (mSAL, lSAL, and nlSAL) were estimated by the whole-genome-regression approach (Meuwissen et al., 2001) which was implemented in the R package “BGLR” (Perez and De Los Campos, 2014). The model describes the element concentration of the *i*th accession in the *j*th environment for each element as follows: *y_ij_ = μ_j_ + g_mSAL_ + g_lSAL_ + g_nlSAL_ + ϵ_ij_*, where *y_ij_* is the element concentration of the *i*th accession measured in the *j*th environment, *μ_j_* is the mean concentration in the *j*th environment, *g_mSAL_* is the genomic correlation matrix (GRM) calculated using all mSALs identified for this element, *g_lSAL_* is the GRM calculated using all lSALs identified for this element, *g_nlSAL_* is the GRM calculated using all nlSALs, and *ϵ_ij_* is the residual error. The whole-genome-regression was not performed on phenotypic measures with SALs less than two.

In order to confirm that the phenotypic variance explained by the lSALs was not caused by its genetic correlation to the mSAL or population structure, a permutation test was performed to generate the null-distribution of the percent of the variance explained (PVE). In each permutation test, the same model was used but the lSALs of each element were replaced by the same number of randomly selected genetic loci. The permutation test was performed 200 times for every element in each environment. All the permutation results were analyzed and illustrated in R.

## Results

### Phenotypic Plasticity Explained a Considerable Proportion of Phenotypic Variance

The concentrations of 16 elements (As, Ca, Cd, Cr, Cu, Fe, K, Mg, Mn, Na, Ni, P, Pb, S, Se, and Zn) in rice grains which were harvested from 575 rice cultivars were measured in four to eight environments that varied in soil biochemical properties, elemental concentrations, and irrigation regimes (**Table S1**). A wide range of responses were observed when averaging elemental concentrations of the diversity panel in each environment (**Figure S1**). Large variations were observed in Cd, Ni, and Se, which showed differences of 108.52-, 50.69-, and 21.89-fold between the highest and lowest mean elemental concentrations in the diversity panel in these environments. Moderate variations were observed in Cr, Pb, Fe, As, and Mg (7.76-, 6.46-, 4.72-, 2.92-, and 2.2-fold changes, respectively), and there were relatively small variations in Zn, Mn, Ca, Cu, K, S, Na, and P (1.96-, 1.91-, 1.88-, 1.59-, 1.24-, 1.20-, 1.17-, and 1.09-fold, respectively). It is worth noting that the range of variation in the non-essential elements (Cd, Ni, Cr, Pb, and As) was larger than that of the essential elements such as Fe, Zn, Mn, and Cu. In total, the concentrations of mineral elements in the rice grain were significantly affected by the planting environment, but the magnitude of the influences varied among elements.

**Figure 1:**
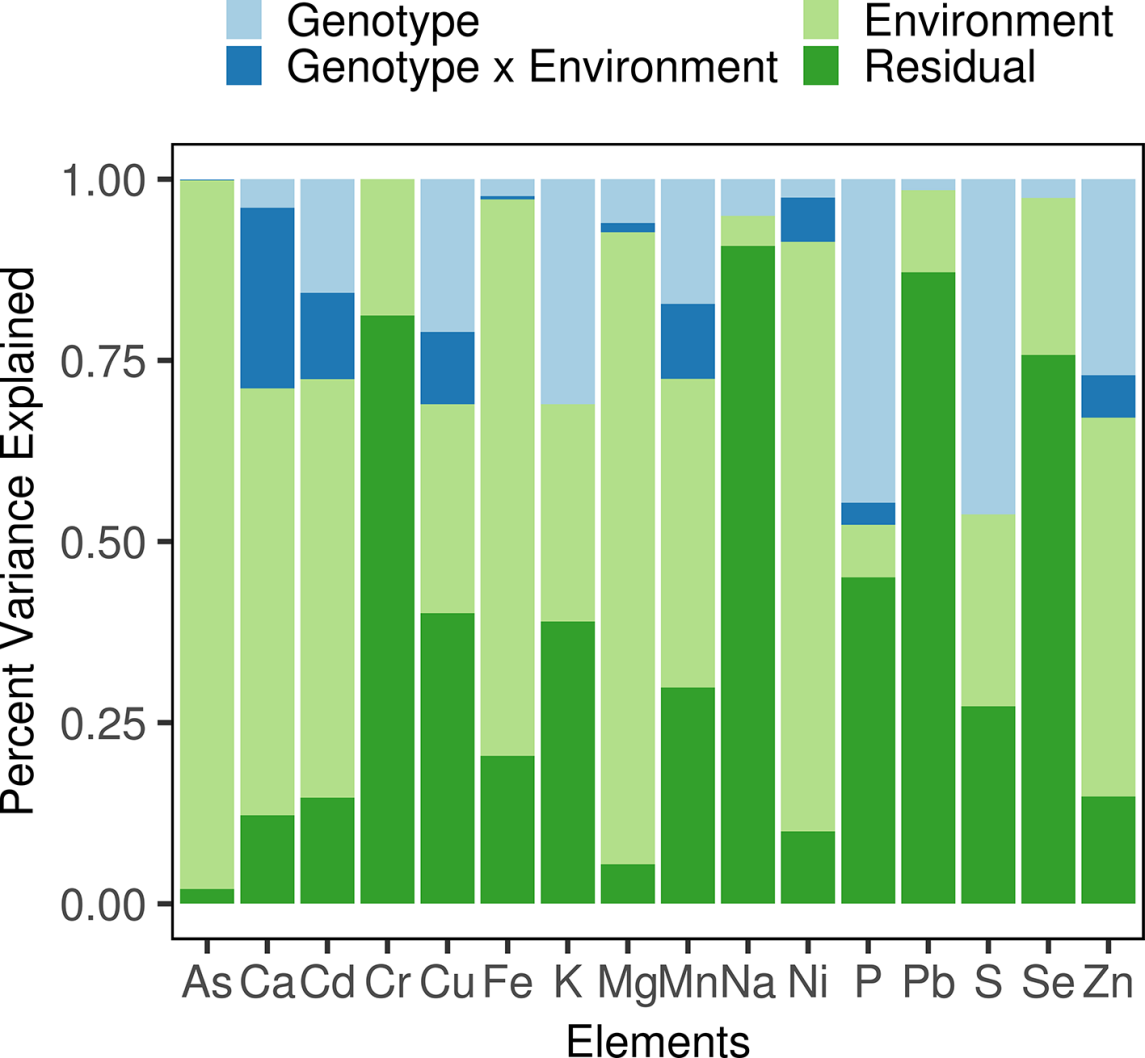
Phenotypic variance component estimation. The proportions of the phenotypic variance attributed to each term were estimated using a linear mixed model and are shown in different colors.

Variation in phenotypic plasticity, termed “genotype-by-environment interaction” (G×E), was observed in the diversity panel. For example, the difference between the mean Cd concentrations measured in 2016Field-2 and 2017Field-1 (2.52 and 0.13 mg/kg, respectively) was 2.39 mg/kg. For each accession in the diversity panel, the differences between Cd concentrations measured in these two environments showed large variations, ranging from 0.25 mg/kg to 4.6 mg/kg with an IQR (interquartile range) of 1.63 mg/kg. We then performed an analysis of variance (ANOVA) for each element to determine the phenotypic variance assigned to the genotype, environment, and G×E (**Figure 1**, **Table S2**). The environment accounted for the largest proportion of phenotypic variance which ranged from 4.18% (Na) to 97.80% (As) with a mean of 43.96%. The phenotypic variance attributed to the genotype ranged from 0.02% (Cr) to 46.25% (S) with a mean of 14.19%. The G×E also explained a considerable proportion of phenotypic variance with a mean of 4.62%: The highest proportion of the variance explained by G×E was observed for Ca (24.91%), followed by Cd (11.88%), Mn (10.33%), Cu (9.97%), Ni (6.10%), and Zn (5.85%), and was relatively low for the other elements (< 5%). In other words, the effect of G×E on elemental accumulation was high in Ca, moderate in Cd, Mn, Cu, Ni, and Zn, while low in the other elements. Therefore, in addition to the environment and genotype which mainly affects the rice grain ionome, the G×E, i.e. the variation in phenotypic plasticity, played a great role in the ionome variation in the diversity panel, especially for Ca, Cd, Mn, Cu, Ni, and Zn.

### Variability in Phenotypic Plasticity

In order to systematically investigate phenotypic plasticity in the diversity panel, we quantified phenotypic plasticity with the Bayesian Finlay-Wilkinson Regression (Bayesian-FWR). Three parameters including mean phenotype, slope, and residual were estimated from the Bayesian-FWR. Specifically, the mean phenotype refers to the main genetic effect of each accession in all environments. The slope was termed linear plasticity, which describes the response of each accession to the planting environment or the macro-environment. The variance of the fitted model's residuals was termed non-linear plasticity, which describes each accession's response to the micro-environment within the planting environment (Finlay and Wilkinson, 1963; Wu, 1998; Kusmec et al., 2017). All elements showed variability in mean phenotype, linear plasticity, and non-linear plasticity (**Figures S2**, **4**). The dispersions of these three phenotypic measures in all elements were investigated by calculating the kurtosis (*g_2_*, the fourth standardized moment of distribution) (**Table S3**). The kurtosis of non-linear plasticity was significantly higher than that of the mean phenotype (*P <*0.05, Wilcoxon rank-sum test), and the kurtosis of linear plasticity was in between them. This observation indicates that the mean phenotype shows the greatest variation in these three phenotypic measures, followed by linear plasticity and non-linear plasticity. The dispersions of linear plasticity were greater than that of non-linear plasticity in the majority of elements (As, Cd, Cr, Cu, Fe, Mg, Mn, Na, Ni, P, Pb, Se, and Zn), suggesting that responses to macro-environments (linear plasticity) show greater variation than responses to micro-environments (non-linear plasticity) in the diversity panel. Comparing the linear plasticity of these elements, As, Mg, S, Cd, and K, showed great dispersion (*g_2_* < 0.5), while P (*g_2_ = * 9.17) and Ca (*g_2_ =* 25.7) showed an apparently centralized distribution.

Next, we investigated the degree of phenotypic plasticity that resulted from genetic variation by calculating the marker-based heritability (*h^2^*) (**Table S4**, **Figure S5**). The mean phenotypes of all elements showed relatively high heritability which ranged from 0.41 (Se) to 0.86 (S) with a mean of 0.61, except for Na (*h^2^* = 0.11). The *h^2^* of linear plasticity was lower than that of mean phenotype but showed greater dispersion, which ranged from 0.02 (Ca) to 0.90 (Cd) with a mean of 0.43. However, the *h^2^* of non-linear plasticity was low and ranged from 0.01 (Pb) to 0.64 (Cd) with a mean of 0.21, which might be explained by the random environmental error that was assigned to non-linear plasticity in the Bayesian-FWR. This indicates that phenotypic plasticity of rice grain ionome is also under genetic control, but shows lower heritability than mean phenotype.

### Phenotypic Plasticity Is Correlated With Mean Phenotype in the Diversity Panel

**Figure 2:**
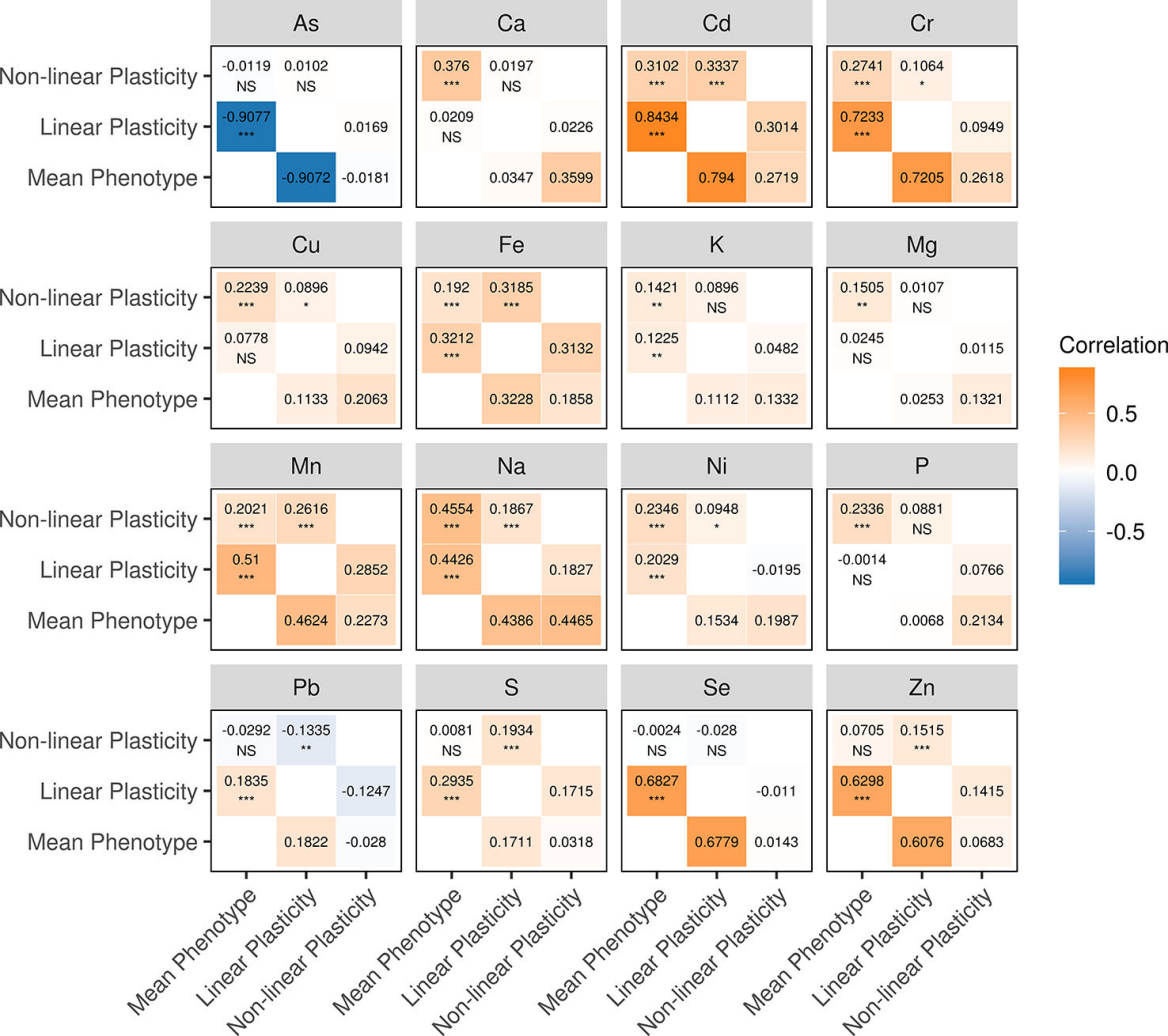
Correlations among the mean phenotype, linear plasticity, and non-linear plasticity for each element. (upper-triangle) The numbers are the values of the Pearson correlation coefficients (*r*). The significance levels (adjusted with the Bonferroni correction) of the correlations are shown below the correlation coefficients: “***” indicates a *P* value < 0.001, “**” indicates a *P* value < 0.01, “*” indicates a *P* value < 0.05, “NS” indicates a *P* value ≥ 0.05. (lower-triangle) The genetic correlation coefficients (*r_g_*).

We examined the relationship between phenotypic plasticity and mean phenotype of the same element by calculating the Pearson correlation coefficient (*r*). It was found that mean phenotype and two measures of phenotypic plasticity tend to correlate in the diversity panel, especially between mean phenotype and linear plasticity (**Figure 2**). A total of 12 elements showed significant correlations (*P <*0.05) between mean phenotype and linear plasticity, followed by the correlation between mean phenotype and non-linear plasticity for 11 elements, and between linear plasticity and non-linear plasticity for 10 elements. Comparing their Pearson correlation coefficients, the highest correlation coefficients were observed between mean phenotype and linear plasticity (Kruskal-Wallis test*, P <*0.05), which ranged from 0.0014 (P) to 0.9077 (As) with a mean of 0.3742. The other two pairs (between mean phenotype and non-linear plasticity, between linear plasticity and non-linear plasticity) had lower Pearson correlation coefficients with means of 0.1823 and 0.1322, respectively. In addition, we also computed their genetic correlation (*r_g_*) with the genotype of the diversity panel, and a similar correlation situation was observed (**Figure 2**).

### The Genetic Architecture of Phenotypic Plasticity Is Distinct From That of Mean Phenotype

A genome-wide association study (GWAS) was performed with the FarmCPU (Fixed and random model circulating probability unification) model using three phenotypic measures (transformed with the Box-Cox procedure) as input to identify their causal genetic loci. Loci with *P* values in excess of the genome-wide threshold (0.05 significance level after Bonferroni correlation) were declared to be significantly associated loci (SALs). A total of 319 SALs were identified, containing 133 SALs related to mean phenotype (mSAL) of 13 elements (except for As, Na, and Pb), 116 SALs related to linear plasticity (lSAL) of 14 elements (except for As and Pb) and 70 SALs related to non-linear plasticity (nlSAL) of 12 elements (except for Ca, K, Na, and P) (**Tables S5**, **Figure S6**–**54**). The total number of SALs related to each element varied from two (Na) to 35 (Zn). In these SALs, a total of 91 SALs were co-located with QTLs or SALs identified in previous studies, which contain 33 mSALs, 40 lSALs, and 18 nlSALs (**Table S5**). In addition, seven previously characterized element-related genes were hit by eight SALs, which contain four mSALs and four lSALs, but no nlSAL (**Table S5**). For example, the Mn transporter *MTP8.1* was hit by the major effect SAL for linear plasticity of Mn identified in the whole panel (*P =* 1.80E-10 at 6,719,733 bp on chromosome 3). The Cd transporter *HMA3* located just near the major effect SAL for the mean phenotype of Cd concentration (*P =* 7.12E-17 at 7,473,929 bp on chromosome 7).

**Figure 3:**
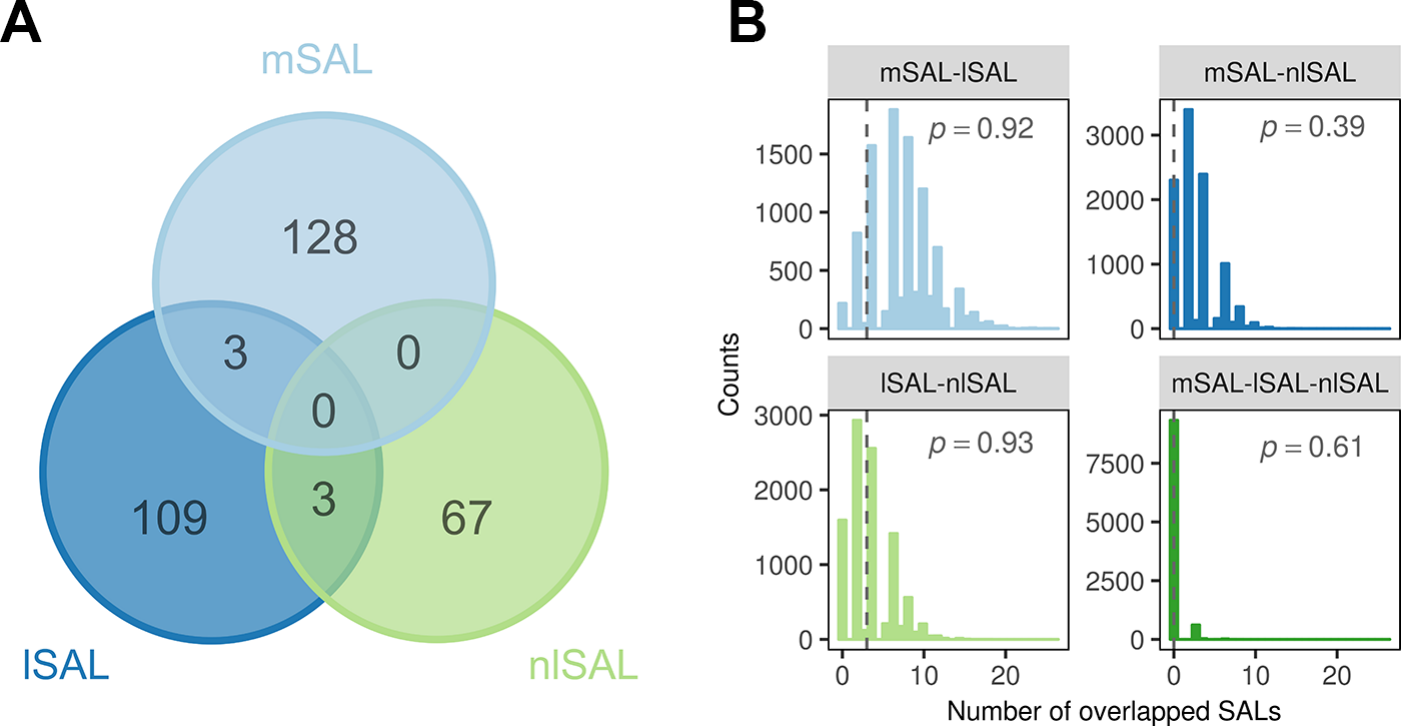
The overlap between three types of SALs for the same element. **(A)** Venn plot of overlap between the mSALs, lSALs, and nlSALs. **(B)** The null-distribution of overlap between different types of SALs estimated from 10,000 permutations. The vertical lines denote the number of overlaps between the three types of SALs identified in this study. The *P* values of the overlaps calculated in this study are labeled in each plot.

We then compared the location of mSALs, lSALs, and nlSALs for the same element. Six chromosomal regions that were overlapped by different types of SALs for the same element were identified (**Figure 3A**). Three of them were overlapped by mSALs and lSALs (two related to Cd, one related to P), the other three were overlapped by lSALs and nlSALs (related to Mg, Mn, and Zn respectively). No chromosomal region was found to be simultaneously overlapped by both mSAL and nlSAL or all three types of SALs. Furthermore, the number of overlapping SALs identified in this study was not greater than that expected by chance in the permutation test (*P > *0.05, **Figure 3B**). Therefore, the majority of the genetic loci accounting for mean phenotype, linear plasticity, and non-linear plasticity of rice grain ionome were distinct, and the majority of the causal genes for them are not the same.

### Population Structure Shaped the Correlation Between Phenotypic Plasticity and Mean Phenotype

**Figure 4:**
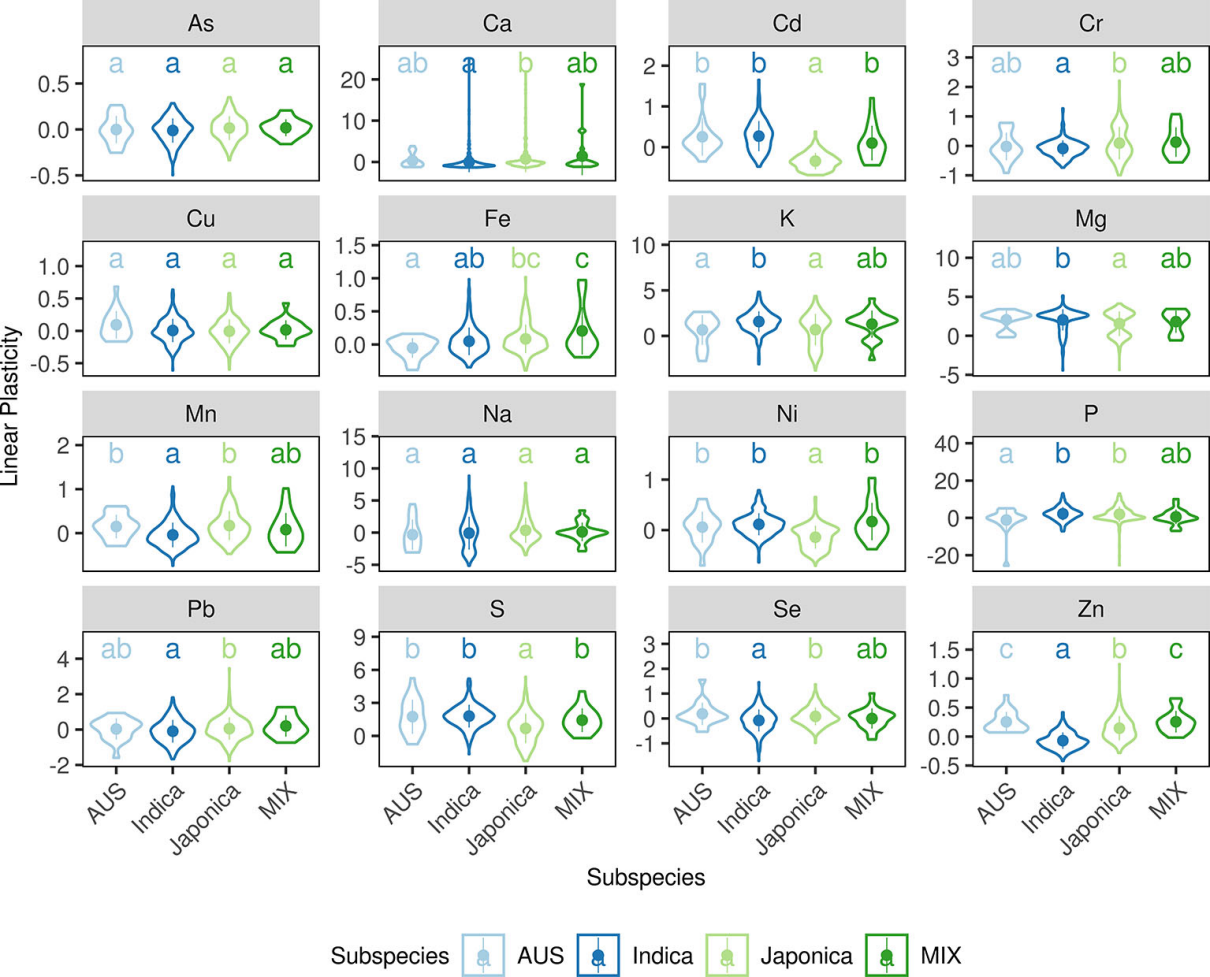
Violin plots of linear plasticity in each rice subspecies. The width of each violin denotes the kernel density, the point and line in each violin denotes the mean value and the standard deviation. The x-axes show the rice subgroups, and the y-axes show the values for linear plasticity. The letters above each violin denote the significant differences between different subspecies (Kruskal-Wallis test, *P* < 0.05).

**Figure 5:**
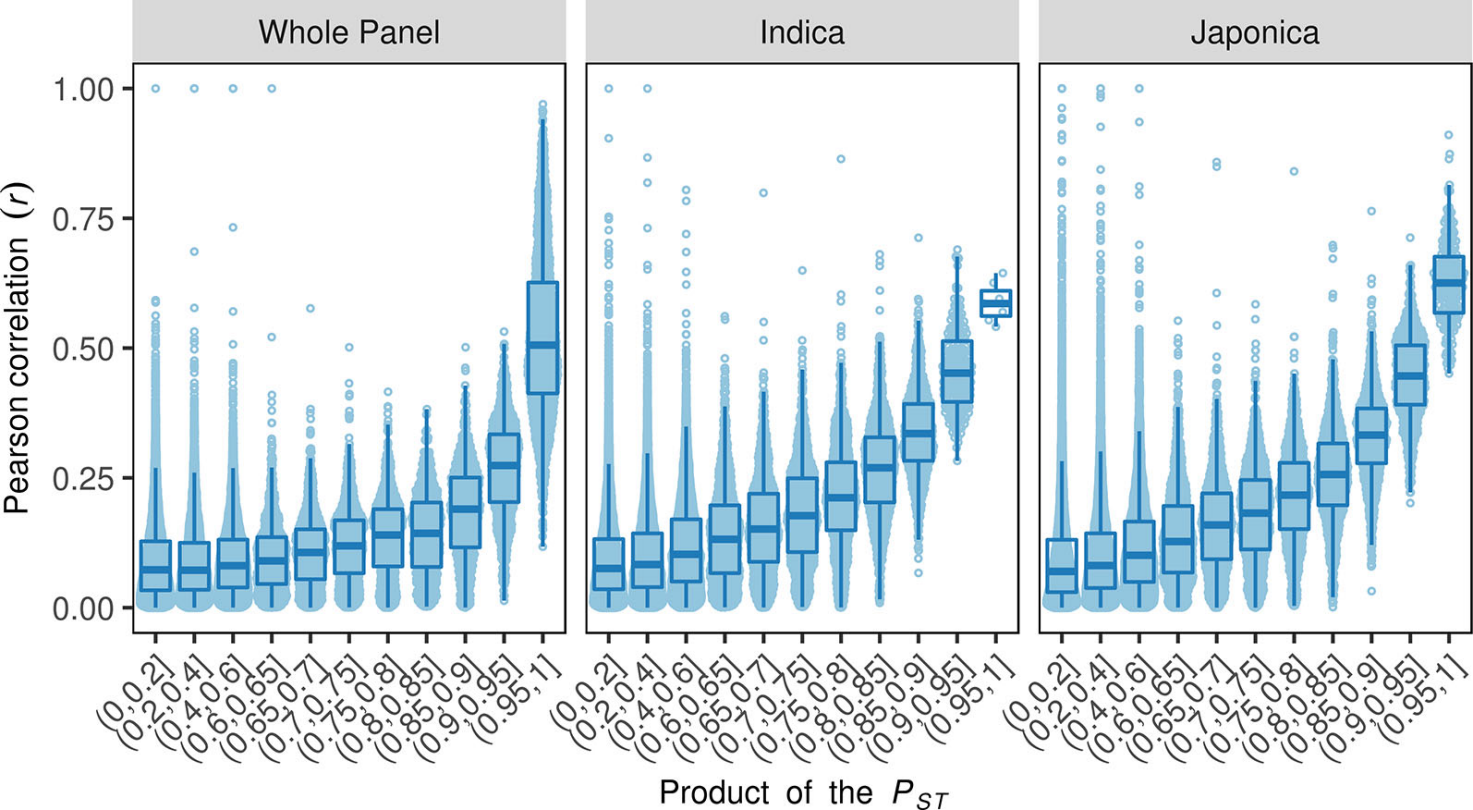
The relationships between the products of the two phenotypes' phenotypic divergence (*P*_ST_) and their correlation in the whole panel and two rice subspecies. The x-axes show the intervals of the product of the two phenotypes' *P*_ST_, the y-axes indicate the absolute value of the Pearson correlation coefficients between the two phenotypes.

A correlation between phenotypic plasticity and mean phenotype was detected in this study (**Figure 2**); however, the fact that overlaps between their corresponding SALs were not greater than expected by chance suggests that the correlations are not due to gene pleiotropy or linkage disequilibrium (**Figure 3**). We then tested the role of population structure, which is also a potential causative factor in addition to gene pleiotropy and linkage disequilibrium, on the correlation between mean phenotype and phenotypic plasticity (Solovieff et al., 2013). Asian rice accessions were highly stratified and were mainly comprised of the two rice subspecies *O. sativa* subsp. *indica* and *O. sativa* subsp. *japonica*. In this study, the phenotypic divergence between *indica* and *japonica* subspecies was observed in three phenotypic measures (**Figures 4** and **S55**-**56**). For example, the accessions in the *indica* subspecies showed higher linear plasticity for Cd, K, Mg, Ni, and S but lower linear plasticity for Ca, Cr, Mn, Pb, Se, and Zn when compared to the accessions in the *japonica* subspecies. With the SNPs identified by whole-genome resequencing, the *indica* subspecies can be further divided into three subgroups: *indica* I, *indica* II, and *indica* intermediate; and the *japonica* subspecies can be further divided into three subgroups: *temperate japonica* (*TEJ*), *tropical japonica* (*TRJ*), and *japonica* intermediate (Tan et al., 2019). The phenotypic divergence was also observed between different rice subgroups (**Figures S57**–**59**). For example, the accessions in the *indica* I subgroup showed significantly lower mean phenotype and linear plasticity for Cd than those from the *indica* II subgroup; however, mean phenotype and linear plasticity of the *indica* I subgroup were higher than *indica* II for Zn. In order to investigate the relationship between the phenotypic divergence and the Pearson correlation coefficients, we used the *P_ST_* index to quantify the phenotypic divergence between two rice subspecies, or between different subgroups in each subspecies (*indica* I and *indica* II in the *indica* subspecies, *TEJ* and *TRJ* in the *japonica* subspecies). A simulation test showed that the Pearson correlation coefficients (*r*) between two phenotypes were positively correlated with the products of the two phenotypes' *P*_ST_ values in the whole panel and two subspecies (**Figure 5**). This suggests that phenotypic divergence is correlated with phenotypic correlation. In this study, varying levels of divergence were observed for mean phenotype (mean *P_ST_* = 0.63), linear plasticity (mean *P_ST_* = 0.55), and non-linear plasticity (mean *P_ST_* = 0.27). For each element, significant correlations (Pearson correlation, *r* = 0.4837, *P* = 0.0005) were found between the products of any two phenotypic measures' *P*_ST_ and the Pearson correlation coefficients between them. Therefore, the population structure of the diversity panel, which leads to the phenotypic divergence, was at least in part responsible for the correlations among the three measures of elements.

### The LSALs Were Valuable in Rice Grain Ionome Improvement Breeding

In order to estimate the value of mSAL, lSAL, and nlSAL in rice grain ionome improvement breeding, a whole-genome regression approach (Gusev et al., 2014; Gage et al., 2017) was used to estimate the proportion of phenotypic variance attributed by these three types of SALs in each environment. Considering that minor allele frequencies (MAFs) of the causal loci may interfere with the estimation of the SALs' PVE, we first compared the MAFs of the three types of SALs, and no significant differences were observed (**Figure S60**, Kruskal-Wallis test, *P > *0.05). Therefore, this possibility was excluded. Large variations were observed when comparing the PVE of the three types of SALs (**Figure S61**, **Table S6**). Overall, the mSALs explained a relatively large proportion of the phenotypic variance for 12 elements which ranged from 0.66% (Ca) to 41.59% (S) with a mean of 13.15%, the lSALs explained a smaller proportion of the phenotypic variance for 13 elements, ranging from 0.83% (Mg) to 33.11% (Zn) with a mean of 8.13%, while the nlSALs explained only a tiny proportion of the phenotypic variance for 10 elements, ranging from 0.16% (Fe) to 3.70% (Se) with a mean of 1.31%. This suggested that mSALs and lSALs are more valuable than nlSALs in breeding. Comparing the PVE of mSAL and lSAL of the same element, the mean PVE of mSALs were higher than for lSALs in Cu, Fe, K, Mg, and P, while they were lower in Ca, Cd, Cr, Mn, Ni, and Zn. It should be noted that the PVE of lSALs was relatively high in Zn (33.11%), Cd (18.19%), Mn (14.96%), Ni (12.69%), K (7.58%), Cr (4.67%), and Cu (4.43%). Thus, the utilization of lSALs in rice breeding depends on the elements and target environments.

**Figure 6:**
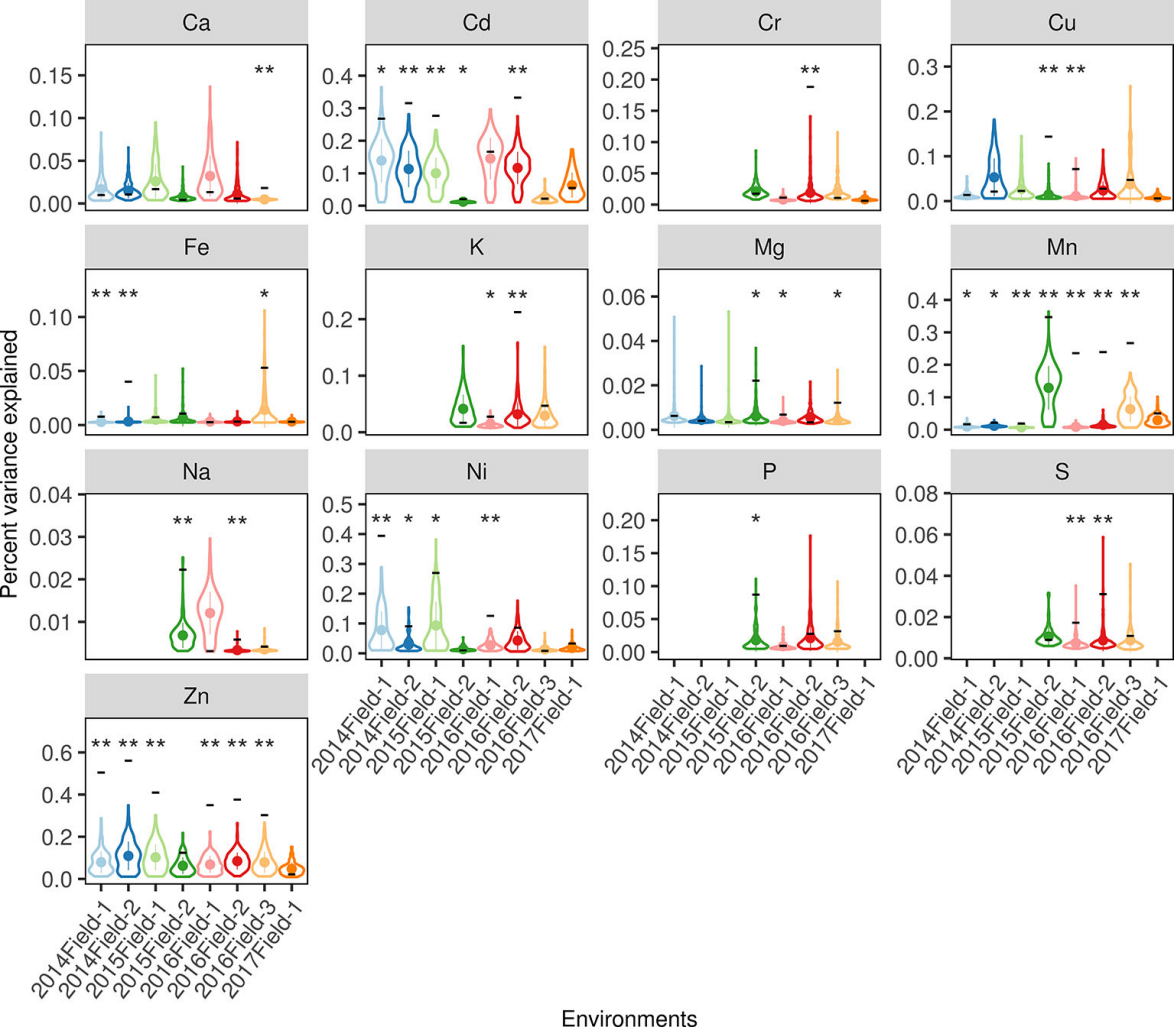
The PVE of the lSALs estimated in each environment. The x-axes indicate the eight planting environments and the y-axes indicate the percent of the variance explained. The violin plots denote the null-distribution of the percent of the variance explained (PVE) estimated from a permutation test with 200 resamplings. The width of each violin denotes the kernel density, the point and line in each violin denotes the mean value and the standard deviation. The black horizontal line located on the violin indicates the real PVE of the lSALs in the corresponding environments. As, Se, and Pb were excluded for their lSALs less than 2. The blanks denote the elemental concentrations were not measured in the corresponding environments. The labels above each violin denote the significance level if the real PVE was significantly higher than the null-distribution: “**” indicates a *P* value < 0.01, “*” indicates a *P* value < 0.05, blank indicates a *P* value > 0.05.

To exclude the possibility that the PVE of lSALs estimated in the whole-genome regression was caused by similarities between the genetic correlation matrix (GRM) of lSALs and the GRM of the whole genome (population structure), a permutation test was performed to compare the PVE of lSALs with randomly selected loci (described in Materials and Methods). The permutation results indicated that the PVE of lSALs was significantly higher than the null-distribution in nearly half of the environments (39/84) for 13 elements (**Figure 6**, permutation test, *P < *0.05). These results confirmed that lSALs contributed a considerable proportion of the phenotypic variance in some environments. Thus, lSALs can also be used in breeding practice, especially for Cd, Cu, Mn, and Zn.

## Discussion

In this paper, the genetic architecture for phenotypic plasticity of rice grain ionome was investigated for the first time using a rice diversity panel. Based on the results of this study, it can be concluded that: (1) A considerable proportion (from 0 to 24.91%) of the rice grain ionome variation could be attributed to the variation of phenotypic plasticity; (2) The genetic architecture of phenotypic plasticity was quite different from that of mean phenotype; (3) The genetic loci involved in phenotypic plasticity of rice grain ionome can be utilized in breeding, especially for Cd, Cu, Mn, and Zn.

The proportions of phenotypic variance attributed to the environment, genotype, and G×E determined the strategy of rice grain ionome improvement. With rice grain ionome measured in up to eight environments, we revealed that the G×E explained a relatively large proportion (from 0 to 24.91%) of phenotypic variance in the diversity panel. It was consistent with the result observed in the agronomic traits of a maize panel (Gage et al., 2017). We then dissected the G×E into linear plasticity and non-linear plasticity with Bayesian-FWR, which reflect the responses of each accession to the macro-environment and micro-environment respectively. The narrow-sense heritabilities (*h^2^*) of phenotypic plasticity were relatively high, especially for linear plasticity of some elements such as Cd, K, Ni, and Zn. The result of our study shows that it is feasible to improve the rice grain ionome by harnessing linear phenotypic plasticity in rice breeding. However, we failed to obtain a convincing relationship between environmental factors and elemental accumulation because some environmental factors correlated in eight environments. In addition, environmental factors fluctuate over the duration of the crop growing season, and the temporal and spatial variation of different environmental factors and their effect on rice grain ionome also need to be investigated. These can be investigated by phenotyping cultivars in a series of environments in which more environmental factors are strictly controlled and systematically recorded, just as quantifying the sensitivities of wheat to water deficiency and high temperature (Parent et al., 2017). All of these studies require not only a high-throughput phenotyping platform but also a high-efficiency environmental characterization system.

Dissecting the genetic architecture of phenotypic plasticity and its relationship to mean phenotype is crucial for breeding cultivars that can adapt to variable planting environments. Three genetic models for phenotypic plasticity, which contain the over-dominance model (Gillespie and Turelli, 1989), the allelic sensitivity model (Via and Lande, 1985; Via, 1993), and the regulatory gene model (Scheiner and Lyman, 1989; Scheiner, 1993), have been proposed. Li et al. (2016) have suggested that phenotypic plasticity of flowering time in maize is more inclined to be regulated by the allelic sensitively model, because most of the environmental response QTLs are shared with flowering time QTLs. Another study of 11 morphological and agronomic traits in maize supports the regulatory gene model for the plastic response to macro-environments, because more genetic loci are located in non-genic regions of the genome (Gage et al., 2017). The study by Kusmec et al. (2017), which investigated 23 phenotypes in maize, strongly supported the regulation model but also found some evidence for the allelic sensitivity model. In our study, we observed that the SALs for mean phenotype and phenotypic plasticity for rice grain ionome were distinct. This observation did not support the allelic sensitivity model but did support the regulatory gene model. In addition, the relatively large linkage disequilibrium (LD) decay distance in rice (~123 kb and ~167 kb in *indica* and *japonica* subspecies, respectively) (Huang et al., 2010) hinders the further dissection of the causal genes and the regulatory network for phenotypic plasticity in this study, such as performing gene ontology (GO) enrichment analysis with candidate genes (Kusmec et al., 2017) and classifying SALs based on their positions to the nearest gene model (Gage et al., 2017). The relatively large LD decay distance in rice also makes it difficult to find accessions with ideal allelic combinations of adjacent genes in rice breeding. Nevertheless, the distinct genetic bases for mean phenotype and phenotypic plasticity revealed in this study indicate that it is feasible to simultaneously utilize genetic loci of them in rice breeding with the abundant alleles provided by the subspecies divergence.

The lSALs contributed to the phenotypic variance in certain environments. These loci can be further utilized in cultivating environment-specific accessions through marker-assisted selection (MAS) or genomic selection (GS). However, both of these strategies were hindered because the correspondence relationship between the genetic locus alleles and the detailed environmental factors were not dissected for the limited amount of data. Further studies that systematically investigate phenotypic plasticity of the rice grain ionome in response to various environmental factors are needed. Firstly, phenotypic plasticity needs to be investigated in a series of widespread planting environments such as the Global Rice Science Partnership (GRiSP) phenotyping network, which covers a broader range of variation on environmental factors. Limited environmental scenarios can be clustered from these planting environments based on their environmental factors. Thereafter, the correspondence relationships between plasticity-related genetic loci and environmental factors or scenarios can be investigated, and the favorable alleles of these genetic loci can be utilized in MAS to cultivate environmental-specific cultivars. In addition, the rice grain ionome recorded in these studies can also be used to perform GS across varied environments, just as the prediction of maize yield under G×E interaction (Millet et al., 2019).

## Data Availability Statement

The raw sequence data used in this paper can be download from the Genome Sequence Archive in the BIG Data Center, Beijing Institute of Genomics (BIG), Chinese Academy of Sciences, under accession numbers CRA000778, CRA000779, and CRA000995 that are publicly accessible at http://bigd.big.ac.cn/gsa.

## Ethics Statement

The authors declare that the experiments comply with the current laws of the country in which they were performed.

## Author Contributions

YT and LS designed the research. YT, JZ, LS, and JW performed the field experiments and elements concentration determination. YT analyzed the data. YT and LS wrote the paper. All the authors read and approved the manuscript.

## Funding

This work was jointly supported by the National Key Research and Development Plan (2017YFD0800901) and the National Natural Science Foundation of China (31470443 and 31501391), and the Key Research Program of the Chinese Academy of Sciences (KFZD-SW-111).

## Conflict of Interest

The authors declare that the research was conducted in the absence of any commercial or financial relationships that could be construed as a potential conflict of interest.
