# Supplementary material for: The Genetic Architecture for Phenotypic Plasticity of the Rice Grain Ionome
Source: Front Plant Sci. 2020 Feb 25;11:12. doi: 10.3389/fpls.2020.00012 (PMC7052182; doi:10.3389/fpls.2020.00012)
Supplement: Supplementary file 1 [file DataSheet_1.pdf]

Supplementary data for

The genetic architecture for phenotypic plasticity of the rice grain ionome

To appear in: *Frontiers in Plant Science*

Authors:

Yongjun Tan[1,2], Jieqiang Zhou [1,3], Jiurong Wang[1], Liang Sun [1#]

[1] Key Laboratory of Agro-Ecological Processes in Subtropical Region, Institute of Subtropical Agriculture, Chinese Academy of Sciences, Changsha, Hunan, 410125, China

[2] University of Chinese Academy of Science, Beijing 100049, China

[3] College of Agronomy, Hunan Agricultural University, Changsha 410128, China

Contact information:

Yongjun Tan (tanyongjun0815@163.com, ORCID: 0000-0003-1041-4912)

Jieqiang Zhou (crackzjq@163.com),

Jiurong Wang (jrwang@isa.ac.cn),

Liang Sun (sunliang037@isa.ac.cn)

[#] Correspondence should be addressed to Liang Sun (sunliang037@isa.ac.cn).

This PDF file include:

Supplemental Table 1-6

Supplemental Figure 1-61

Table S1 List of all planting environments

Environments	2014Field-1	2014Field-2	2015Field-1	2015Field-2	2016Field-1	2016Field-2	2016Field-3	2017Field-1
Position	Changsha, Hunan,China	Changsha, Hunan,China	Changsha, Hunan,China	Songjiang, Shanghai,China	Changsha, Hunan,China	Changsha, Hunan,China	Changsha, Hunan,China	Chenzhou, Hunan,China
Latitude	28°44'N	28°44'N	28°44'N	30°94'N	28°44'N	28°44'N	28°19'N	25°70'N
Longitude	113°06'E	113°06'E	113°06'E	121°13'E	113°06'E	113°06'E	113°08'E	112°71'E
Irrigation[a]	Unflooded	Unflooded	Unflooded	Unflooded	Unflooded	Unflooded	Unflooded	Flooded
Replicates	3	3	3	1	3	3	1	3
As (μg/g)	NA	NA	NA	12.3±0.17	13.1±0.16	10.7±0.31	25.2±0.34	54.0±2.4
Ca(mg/g)	2.34±0.08	2.15±0.10	2.37±0.19	3.24±0.13	2.85±0.21	2.75±0.16	2.37±0.07	NA
Cd (μg/g)	1.43±0.18	1.62±0.25	1.52±0.19	0.06±0.01	1.22±0.2	0.56±0.19	0.09±0.02	1.30±0.15
Cr (μg/g)	NA	NA	NA	NA	42.7±5.7	56.1±3.7	39.7±4.1	41.6±3.8
Cu (μg/g)	19.5±1.9	18.5±2.7	23.7±1.8	24.6±3.4	20.2±2.6	25.9±4.1	16.4±1.8	22.5±2.5
Fe (mg/g)	32±4.1	52±4.3	52±3.7	34±3.9	24±4.1	39±5.2	51±3.8	43±2.8
K(mg/g)	NA	NA	NA	4.21±0.3	3.97±0.26	4.02±0.15	3.78±0.08	NA
Mg(mg/g)	2.06±0.15	2.14±0.21	3.16±0.14	3.76±0.11	3.31±0.20	3.41±0.18	3.67±0.24	NA
Mn (μg/g)	268.4±13.2	189.3±12.9	248.2±15.7	198.08±8.4	256.6±15.8	208.7±13.4	428.1±20.5	234.7±19.5
N(mg/g)	NA	NA	2.41±0.15	2.79±0.09	2.73±0.16	2.61±0.14	2.78±0.10	3.12±0.17
Ni (μg/g)	38.6±5.3	15.3±3.4	20.1±5.7	6.8±2.9	14.8±2.9	11.5±4.1	3.5±1.2	2.9±2.1
P(μg/g)	NA	NA	NA	19±3.7	31±4.2	29±6.1	24±4.9	NA
Pb (μg/g)	94.1±6.2	25.5±5.4	87.7±9.2	76.1±6.6	94.6±6.2	84.4±5.1	36.8±5.4	86.2±6.7
S(mg/g)	NA	NA	NA	0.26±0.02	0.31±0.04	0.46±0.02	0.37±0.03	NA
Se (μg/g)	NA	NA	NA	0.25±0.03	0.49±0.12	0.82±0.21	0.48±0.07	0.46±0.15
Zn (μg/g)	119.4±7.5	78.3±3.7	134.9±6.7	52.4±3.8	126.3±8.4	119.6±4.1	107.3±3.4	94.5±6.1
pH	5.8±0.2	6.2±0.3	5.2±0.1	6.4±0.2	5.5±0.1	5.2±0.2	6.7±0.2	7.8±0.4
Sowing date	2014.05.30	2014.05.30	2015.06.1	2015.06.8	2016.05.25	2016.05.25	2016.05.27	2017.06.13

A total of 5-13 soil samples (0-15 cm depth) were collected after harvest in each field for analyzing the total elemental concentration and pH of soil. Data are means ± SD of all samples.

a: The water regime in the period from the flowering of the first accession to the harvest of the last accession. The unflooded field was flush irrigated about six hours when needed to prevent water stress.

Table S2 Phenotypic variance partition with analysis of variance (ANOVA)

Source	As	Ca	Cd	Cr	Cu	Fe	K	Mg	Mn	Na	Ni	P	Pb	S	Se	Zn	Mean
Genoeype(G)	0.05%	3.94%	15.69%	0.02%	21.07%	2.34%	31.06%	6.03%	17.21%	5.05%	2.55%	44.66%	1.52%	46.25%	2.59%	27.06%	14.19%
Environment(E)	97.80%	58.92%	57.76%	18.82%	28.87%	76.81%	29.97%	87.20%	42.63%	4.18%	81.36%	7.24%	11.31%	26.49%	21.65%	52.30%	43.96%
GxE	0.12%	24.91%	11.88%	0.01%	9.97%	0.43%	0.00%	1.30%	10.33%	0.00%	6.10%	3.03%	0.00%	0.01%	0.00%	5.85%	4.62%
Residual	2.03%	12.22%	14.67%	81.18%	40.09%	20.42%	38.96%	5.48%	29.83%	90.77%	9.99%	45.07%	87.17%	27.24%	75.77%	14.79%	37.23%

Table S3 The kurtosis (g_2) of each phenotypic measure

	As	Ca	Cd	Cr	Cu	Fe	K	Mg	Mn	Na	Ni	P	Pb	S	Se	Zn
Mean Phenotype	0.02	0.39	-0.16	-0.46	0.27	0.80	0.43	0.74	1.23	0.97	4.05	0.42	0.64	0.35	0.54	-0.23
Linear Plasticity	0.08	25.66	0.46	2.09	0.95	2.45	0.46	0.33	0.70	0.60	0.58	9.17	1.39	0.39	1.11	2.16
Non-linear Plasticity	6.63	-0.11	3.82	6.72	6.93	6.20	0.34	0.90	6.91	1.62	14.42	1.79	15.89	-0.16	6.49	11.14

Table S4 Marker-based heritability (h^2) of three phenotypic measures for each element

	As	Ca	Cd	Cr	Cu	Fe	K	Mg	Mn	Na	Ni	P	Pb	S	Se	Zn
Mean Phenotype	0.50	0.57	0.50	0.50	0.62	0.54	0.76	0.82	0.53	0.11	0.53	0.82	0.50	0.86	0.41	0.61
Linear Plasticity	0.12	0.02	0.90	0.26	0.41	0.39	0.66	0.56	0.58	0.32	0.74	0.32	0.02	0.47	0.33	0.73
Non-linear Plasticity	0.03	0.36	0.64	0.04	0.03	0.05	0.07	0.60	0.22	0.13	0.13	0.38	0.01	0.40	0.07	0.14

Table S5 List of all identified SALs(significantly associated loci)

Phenotypic Measures	Elements	Chr	Pos	P.value (GWAS)	Panel	MAF	PVE (Phenotypic measures)	Significant Region Start(Mb)	Significant Region End(Mb)	Allele Effect (Nip)	Overlap (Between Panels)	Overlap (Between Measures)	Reference	Genes
Mean Phenotype	Ca	1	1,683,482	1.05E-08	Whole Panel	0.40	1.92%	1.43	1.93	Low				
Mean Phenotype	Ca	5	3,646,613	1.49E-08	Whole Panel	0.47	0.00%	3.58	3.78	Low				
Mean Phenotype	Ca	11	1,029,853	1.09E-08	Whole Panel	0.08	7.11%	0.94	1.14	High				
Mean Phenotype	Cd	1	795,990	7.33E-10	Whole Panel	0.42	16.89%	0.55	1.05	High			q	
Mean Phenotype	Cd	4	33,879,941	7.67E-11	*Indica*	0.06	5.92%	33.78	33.98	Low				
Mean Phenotype	Cd	5	7,349,022	7.79E-09	Whole Panel	0.43	4.75%	7.10	7.60	High			q	
Mean Phenotype	Cd	7	7,473,929	7.12E-17	*Indica*	0.10	18.73%	7.22	7.72	High			g	*HMA3*
Mean Phenotype	Cd	7	8,430,087	6.01E-13	Whole Panel	0.47	16.89%	8.18	8.68	High		Y	a	
Mean Phenotype	Cd	7	10,293,897	3.94E-16	Whole Panel	0.10	0.07%	10.04	10.54	Low				
Mean Phenotype	Cd	7	16,637,858	9.99E-09	Whole Panel	0.34	11.89%	16.39	16.89	High				
Mean Phenotype	Cd	8	8,735,225	4.28E-09	Whole Panel	0.32	0.09%	8.64	8.84	Low				
Mean Phenotype	Cd	9	6,130,081	2.50E-10	*Indica*	0.37	20.38%	6.03	6.23	Low	Y	Y		
Mean Phenotype	Cd	9	6,130,081	3.08E-09	Whole Panel	0.21	0.01%	6.12	6.56	Low	Y	Y		
Mean Phenotype	Cd	10	280,882	1.73E-08	Whole Panel	0.08	0.00%	0.19	0.39	High				
Mean Phenotype	Cd	11	2,446,720	1.76E-12	*Indica*	0.34	0.11%	2.33	2.53	Low				*ENA1*
Mean Phenotype	Cd	11	10,244,670	1.18E-10	Whole Panel	0.26	0.77%	9.99	10.49	Low				
Mean Phenotype	Cd	11	24,068,075	6.76E-09	Whole Panel	0.41	2.25%	23.86	24.29	Low			q	
Mean Phenotype	Cd	11	28,454,193	9.67E-09	*Indica*	0.24	20.38%	28.09	28.49	Low				
Mean Phenotype	Cr	3	17,957,999	1.07E-08	*Japonica*	0.07	10.33%	17.86	18.06	Low				
Mean Phenotype	Cr	7	4,921,009	7.15E-10	*Japonica*	0.47	10.33%	4.67	5.17	Low				
Mean Phenotype	Cr	11	6,584,782	7.76E-10	*Japonica*	0.15	10.33%	6.48	6.68	Low				
Mean Phenotype	Cr	12	17,801,781	1.29E-08	*Japonica*	0.14	10.33%	17.58	17.92	High				
Mean Phenotype	Cu	1	35,202,111	6.87E-12	*Japonica*	0.29	11.60%	34.98	35.22	Low			k,m	
Mean Phenotype	Cu	2	31,630,506	1.13E-08	Whole Panel	0.44	2.89%	31.38	31.88	Low				
Mean Phenotype	Cu	3	15,448,987	2.53E-08	*Japonica*	0.10	5.26%	15.34	15.54	High				
Mean Phenotype	Cu	6	19,265,035	2.95E-10	Whole Panel	0.06	2.89%	19.02	19.52	High			k	
Mean Phenotype	Cu	8	8,788,169	1.29E-10	Whole Panel	0.33	2.82%	8.54	9.04	Low			i,m	
Mean Phenotype	Cu	11	2,434,306	8.74E-12	Whole Panel	0.20	0.44%	2.22	2.48	High			m	*ENA1*
Mean Phenotype	Cu	11	6,159,144	1.56E-10	*Japonica*	0.05	11.60%	6.06	6.26	High			r	
Mean Phenotype	Cu	11	20,514,087	2.19E-10	*Japonica*	0.43	1.87%	20.41	20.61	Low				
Mean Phenotype	Fe	4	802,734	1.04E-14	Whole Panel	0.08	12.90%	0.69	0.97	High				

Phenotypic Measures	Elements	Chr	Pos	P.value (GWAS)	Panel	MAF	PVE (Phenotypic measures)	Significant Region Start(Mb)	Significant Region End(Mb)	Allele Effect (Nip)	Overlap (Between Panels)	Overlap (Between Measures)	Reference	Genes
Mean Phenotype	Fe	5	5,835,605	1.30E-13	Whole Panel	0.24	12.90%	5.72	5.92	Low				
Mean Phenotype	Fe	5	27,539,473	6.88E-09	Whole Panel	0.08	9.22%	27.45	27.65	High				
Mean Phenotype	Fe	8	2,797,478	1.89E-14	Whole Panel	0.32	8.73%	2.72	2.92	Low				
Mean Phenotype	Fe	8	8,506,328	4.15E-08	*Japonica*	0.07	13.70%	8.41	8.61	High			r	
Mean Phenotype	Fe	8	20,439,074	2.06E-09	Whole Panel	0.09	5.87%	20.33	20.53	High				
Mean Phenotype	Fe	11	13,583,539	4.37E-08	*Japonica*	0.07	2.85%	13.48	13.68	Low				
Mean Phenotype	Fe	11	16,231,792	8.31E-19	*Japonica*	0.07	14.50%	16.13	16.33	High				
Mean Phenotype	Fe	12	2,197,415	3.21E-12	*Japonica*	0.27	3.65%	2.09	2.29	High			j	
Mean Phenotype	K	3	33,239,260	2.74E-08	*Indica*	0.11	11.87%	32.99	33.49	Low				
Mean Phenotype	K	6	1,792,792	6.96E-14	*Indica*	0.48	2.19%	1.54	2.04	Low	Y		d,o,q	
Mean Phenotype	K	6	1,792,792	5.90E-13	Whole Panel	0.31	0.01%	1.54	2.04	Low	Y		d,o,q	
Mean Phenotype	K	6	11,125,709	9.66E-10	Whole Panel	0.11	20.42%	11.03	11.23	High				
Mean Phenotype	K	6	18,141,616	2.01E-08	*Japonica*	0.17	11.87%	17.89	18.39	High			o	
Mean Phenotype	K	7	13,556,162	9.38E-09	Whole Panel	0.29	3.47%	13.31	13.81	High				
Mean Phenotype	K	8	8,820,787	2.11E-10	*Indica*	0.19	18.55%	8.73	8.93	Low				
Mean Phenotype	K	9	21,115,786	1.55E-15	Whole Panel	0.17	12.65%	20.91	21.24	High			b,n	
Mean Phenotype	Mg	1	27,525,475	6.25E-09	*Indica*	0.13	0.11%	27.43	27.63	High				
Mean Phenotype	Mg	1	36,346,000	1.38E-08	Whole Panel	0.16	9.06%	36.30	36.50	Low				
Mean Phenotype	Mg	2	12,199,639	1.17E-10	*Japonica*	0.33	10.83%	12.13	12.33	Low				
Mean Phenotype	Mg	2	14,212,780	4.68E-12	*Japonica*	0.05	16.38%	13.96	14.46	High				
Mean Phenotype	Mg	3	33,203,042	6.58E-16	*Indica*	0.11	15.63%	32.98	33.34	Low			n	
Mean Phenotype	Mg	6	8,819,531	2.73E-10	Whole Panel	0.12	9.10%	8.57	9.07	High				
Mean Phenotype	Mg	6	12,292,040	5.59E-13	*Indica*	0.22	7.41%	12.14	12.34	Low				
Mean Phenotype	Mg	6	24,560,232	3.61E-09	*Indica*	0.22	2.76%	24.46	24.66	Low			r	
Mean Phenotype	Mg	7	497,481	2.39E-09	Whole Panel	0.12	3.42%	0.40	0.60	High				
Mean Phenotype	Mg	7	9,836,660	6.09E-11	Whole Panel	0.13	17.50%	9.59	10.09	High				
Mean Phenotype	Mg	7	25,313,250	2.67E-09	Whole Panel	0.06	0.97%	25.11	25.32	High				
Mean Phenotype	Mg	9	15,773,490	4.39E-13	*Indica*	0.31	5.92%	15.35	15.80	High			b	
Mean Phenotype	Mg	10	20,599,205	7.23E-14	*Japonica*	0.06	7.24%	20.19	20.67	Low				
Mean Phenotype	Mg	11	19,813,409	5.20E-10	Whole Panel	0.10	15.78%	19.71	19.91	High				
Mean Phenotype	Mg	11	25,804,573	1.47E-08	Whole Panel	0.11	0.35%	25.55	25.90	Low				
Mean Phenotype	Mg	11	26,819,570	1.18E-10	Whole Panel	0.46	18.74%	26.73	26.93	High	Y			

Phenotypic Measures	Elements	Chr	Pos	P.value (GWAS)	Panel	MAF	PVE (Phenotypic measures)	Significant Region Start(Mb)	Significant Region End(Mb)	Allele Effect (Nip)	Overlap (Between Panels)	Overlap (Between Measures)	Reference	Genes
Mean Phenotype	Mg	11	26,965,337	1.90E-09	*Indica*	0.44	15.79%	26.72	27.22	High	Y			
Mean Phenotype	Mg	12	2,240,721	1.16E-10	Whole Panel	0.36	2.25%	1.99	2.49	Low			r	
Mean Phenotype	Mg	12	27,065,837	3.77E-10	*Indica*	0.26	0.71%	26.97	27.17	High				
Mean Phenotype	Mn	3	8,642,556	1.13E-08	Whole Panel	0.18	2.94%	8.39	8.89	High				
Mean Phenotype	Mn	4	904,276	1.07E-09	Whole Panel	0.32	2.94%	0.65	1.15	Low				
Mean Phenotype	Mn	5	27,900,026	1.04E-08	*Indica*	0.35	0.91%	27.84	28.16	High			i	
Mean Phenotype	Mn	5	29,360,515	6.15E-09	*Indica*	0.08	0.91%	29.21	29.45	High				
Mean Phenotype	Mn	9	21,541,460	1.04E-08	Whole Panel	0.25	1.01%	21.36	21.56	Low				
Mean Phenotype	Mn	11	8,513,929	2.33E-10	Whole Panel	0.24	0.31%	8.26	8.76	High			o	
Mean Phenotype	Mn	11	17,899,745	1.83E-13	Whole Panel	0.22	2.94%	17.75	17.95	High	Y			
Mean Phenotype	Mn	11	17,941,309	1.28E-08	*Indica*	0.15	0.91%	17.69	18.19	High	Y			
Mean Phenotype	Mn	11	20,854,832	2.57E-08	*Indica*	0.20	0.91%	20.84	21.05	High	Y		r	
Mean Phenotype	Mn	11	21,138,486	1.78E-08	Whole Panel	0.07	2.94%	20.89	21.15	High	Y		k,r	
Mean Phenotype	Ni	1	21,156,160	1.25E-09	Whole Panel	0.09	12.41%	20.91	21.41	Low				
Mean Phenotype	Ni	3	15,514,427	4.33E-11	Whole Panel	0.31	11.73%	15.42	15.62	High				
Mean Phenotype	Ni	4	26,107,854	1.72E-08	*Japonica*	0.09	8.28%	25.86	26.36	High				
Mean Phenotype	Ni	6	26,674,937	1.05E-09	Whole Panel	0.49	15.03%	26.42	26.92	Low				
Mean Phenotype	Ni	8	5,567,280	2.65E-08	*Indica*	0.07	10.52%	5.46	5.66	High				
Mean Phenotype	Ni	9	14,238,893	5.66E-09	Whole Panel	0.10	14.92%	14.16	14.48	High				
Mean Phenotype	P	2	12,199,639	4.63E-10	Whole Panel	0.28	15.43%	11.95	12.45	Low	Y			
Mean Phenotype	P	2	12,199,639	2.55E-14	*Japonica*	0.33	3.39%	12.13	12.33	Low	Y			
Mean Phenotype	P	2	29,123,952	2.16E-08	*Indica*	0.12	0.26%	29.00	29.34	High			o,r	
Mean Phenotype	P	4	13,505,438	1.75E-12	*Indica*	0.19	20.05%	13.47	13.81	High				
Mean Phenotype	P	4	20,701,268	2.25E-11	*Japonica*	0.14	18.46%	20.61	20.81	High				
Mean Phenotype	P	6	7,776,248	1.02E-09	*Japonica*	0.05	2.17%	7.70	7.90	High				
Mean Phenotype	P	7	13,633,881	7.33E-17	Whole Panel	0.10	16.02%	13.38	13.88	High				
Mean Phenotype	P	7	20,394,327	2.14E-09	Whole Panel	0.07	10.81%	20.14	20.64	High				
Mean Phenotype	P	10	1,354,405	4.87E-09	*Indica*	0.47	9.16%	1.12	1.40	Low				
Mean Phenotype	P	10	4,193,962	2.13E-09	Whole Panel	0.27	0.79%	3.94	4.44	High				
Mean Phenotype	P	10	17,950,353	2.38E-11	Whole Panel	0.09	0.04%	17.70	18.20	Low				
Mean Phenotype	P	11	19,813,393	3.38E-10	*Japonica*	0.45	16.40%	19.71	19.91	High				
Mean Phenotype	P	11	20,938,745	4.16E-08	*Japonica*	0.15	0.06%	20.69	21.19	High				

Phenotypic Measures	Elements	Chr	Pos	P.value (GWAS)	Panel	MAF	PVE (Phenotypic measures)	Significant Region Start(Mb)	Significant Region End(Mb)	Allele Effect (Nip)	Overlap (Between Panels)	Overlap (Between Measures)	Reference	Genes
Mean Phenotype	P	11	28,129,959	2.17E-16	*Indica*	0.10	20.05%	27.88	28.38	High		Y		
Mean Phenotype	P	11	28,743,343	6.81E-10	Whole Panel	0.09	16.72%	28.49	28.99	High		Y		
Mean Phenotype	P	12	8,928,232	8.15E-18	*Indica*	0.44	5.92%	8.68	9.18	High				
Mean Phenotype	P	12	12,162,328	2.74E-08	*Indica*	0.10	9.90%	12.06	12.26	Low				
Mean Phenotype	S	1	3,085,472	4.17E-09	Whole Panel	0.22	0.61%	2.84	3.34	High	Y			
Mean Phenotype	S	1	3,095,195	1.53E-14	*Japonica*	0.38	12.44%	2.85	3.35	Low	Y			
Mean Phenotype	S	1	4,193,983	1.36E-13	*Indica*	0.06	16.56%	4.10	4.30	Low				
Mean Phenotype	S	1	29,781,448	5.61E-12	Whole Panel	0.18	3.40%	29.68	29.88	High			r	
Mean Phenotype	S	3	7,617,517	6.50E-09	Whole Panel	0.12	3.40%	7.37	7.87	Low				
Mean Phenotype	S	3	29,032,123	2.28E-09	Whole Panel	0.19	0.27%	28.78	29.28	Low			r	
Mean Phenotype	S	5	8,221,364	1.34E-11	Whole Panel	0.27	3.40%	7.97	8.47	High	Y			
Mean Phenotype	S	5	8,334,198	3.04E-09	*Indica*	0.22	6.15%	8.23	8.43	Low	Y			
Mean Phenotype	S	5	23,266,629	1.64E-08	Whole Panel	0.41	0.87%	23.02	23.52	High				
Mean Phenotype	S	5	24,441,905	5.37E-13	*Indica*	0.16	0.23%	24.19	24.69	Low				
Mean Phenotype	S	6	7,210,104	1.11E-09	Whole Panel	0.30	3.40%	6.96	7.46	High				
Mean Phenotype	S	6	8,879,934	4.97E-08	*Japonica*	0.08	12.44%	8.85	9.05	High	Y			
Mean Phenotype	S	6	9,035,697	2.08E-09	Whole Panel	0.28	3.40%	8.79	9.29	Low	Y			
Mean Phenotype	S	7	2,235,815	1.38E-08	*Japonica*	0.18	8.75%	2.14	2.34	High				
Mean Phenotype	S	7	4,562,470	1.67E-08	*Indica*	0.35	15.85%	4.46	4.66	High				
Mean Phenotype	S	7	17,156,503	2.28E-09	Whole Panel	0.40	0.02%	16.91	17.41	High			r	
Mean Phenotype	S	8	7,825,922	1.37E-08	*Indica*	0.13	8.62%	7.73	7.93	High				
Mean Phenotype	S	9	20,009,713	1.20E-08	*Japonica*	0.13	6.70%	19.90	20.21	High			r	
Mean Phenotype	S	11	25,049,208	3.89E-08	*Japonica*	0.26	0.40%	24.86	25.17	Low				
Mean Phenotype	S	11	26,963,127	2.33E-19	*Indica*	0.30	18.68%	26.71	27.21	High	Y			
Mean Phenotype	S	11	26,963,127	2.29E-13	Whole Panel	0.16	3.40%	26.71	27.21	High	Y			
Mean Phenotype	S	12	16,565,856	7.42E-10	Whole Panel	0.44	3.40%	16.32	16.82	High				
Mean Phenotype	S	12	21,536,923	1.30E-09	*Indica*	0.13	9.44%	21.44	21.64	High				
Mean Phenotype	Se	1	23,589,633	1.03E-08	Whole Panel	0.06	16.10%	23.30	23.64	High				
Mean Phenotype	Zn	2	29,834,911	5.87E-17	*Indica*	0.07	14.76%	29.76	29.96	Low			c,p,r	
Mean Phenotype	Zn	2	34,966,943	5.15E-09	*Indica*	0.42	0.01%	34.84	35.04	Low				
Mean Phenotype	Zn	3	7,371,940	1.44E-08	*Japonica*	0.28	7.61%	7.12	7.62	Low				*DMAS1*
Mean Phenotype	Zn	3	9,205,841	2.20E-08	*Japonica*	0.08	7.61%	9.08	9.28	High				

Phenotypic Measures	Elements	Chr	Pos	P.value (GWAS)	Panel	MAF	PVE (Phenotypic measures)	Significant Region Start(Mb)	Significant Region End(Mb)	Allele Effect (Nip)	Overlap (Between Panels)	Overlap (Between Measures)	Reference	Genes
Mean Phenotype	Zn	3	11,205,562	5.17E-08	*Japonica*	0.16	7.61%	11.08	11.28	Low			j,n	
Mean Phenotype	Zn	3	29,335,674	3.94E-09	*Indica*	0.46	11.19%	29.31	29.65	Low				
Mean Phenotype	Zn	4	33,597,164	1.68E-08	Whole Panel	0.25	14.91%	33.49	33.69	Low			m	
Mean Phenotype	Zn	8	20,686,031	2.70E-08	*Indica*	0.06	12.16%	20.65	20.99	High			d,n	
Mean Phenotype	Zn	12	103,541	4.67E-09	*Indica*	0.12	2.78%	-0.15	0.35	High				
Mean Phenotype	Zn	12	15,809,441	5.00E-10	*Indica*	0.47	0.24%	15.56	16.06	High				
Linear Plasticity	Ca	2	7,939,328	1.71E-08	*Indica*	0.08	1.10%	7.84	8.04	Low			d	
Linear Plasticity	Ca	2	14,724,782	9.49E-09	*Indica*	0.07	1.10%	14.62	14.82	Low				
Linear Plasticity	Ca	2	25,151,605	1.72E-08	*Indica*	0.18	1.10%	24.90	25.40	Low				
Linear Plasticity	Ca	12	770,389	7.07E-09	*Indica*	0.05	1.10%	0.67	0.87	Low				
Linear Plasticity	Cd	2	25,113,972	1.64E-09	*Indica*	0.26	4.62%	24.86	25.36	Low			q	*CAL*
Linear Plasticity	Cd	5	23,479,652	8.00E-11	*Indica*	0.20	5.17%	23.29	23.49	Low				
Linear Plasticity	Cd	5	29,749,551	8.44E-11	*Indica*	0.19	5.99%	29.65	29.85	Low				
Linear Plasticity	Cd	6	8,556,505	4.55E-09	Whole Panel	0.50	8.78%	8.31	8.81	High				
Linear Plasticity	Cd	7	6,967,567	4.91E-20	*Indica*	0.14	14.04%	6.72	7.22	High			a,g,r	
Linear Plasticity	Cd	7	8,311,571	2.19E-16	Whole Panel	0.43	7.20%	8.06	8.56	High		Y	a	
Linear Plasticity	Cd	7	9,161,046	9.63E-10	*Indica*	0.49	14.04%	8.90	9.35	High			q,r	*NRAMP1*
Linear Plasticity	Cd	8	19,262,502	5.41E-10	*Japonica*	0.20	11.15%	19.19	19.39	High				
Linear Plasticity	Cd	8	21,150,062	6.25E-11	*Indica*	0.06	9.37%	21.09	21.32	Low			r	
Linear Plasticity	Cd	8	26,913,733	9.74E-09	Whole Panel	0.14	8.78%	26.79	26.99	High	Y		q	
Linear Plasticity	Cd	8	26,968,333	3.77E-10	*Indica*	0.08	3.66%	26.94	27.18	High	Y		q	
Linear Plasticity	Cd	9	6,131,885	2.44E-08	*Indica*	0.17	14.04%	6.03	6.23	High		Y		
Linear Plasticity	Cd	9	8,983,294	1.68E-09	Whole Panel	0.28	7.20%	8.73	9.23	High				
Linear Plasticity	Cd	12	12,985,244	4.46E-11	Whole Panel	0.05	8.78%	12.89	13.09	High			q	
Linear Plasticity	Cr	1	16,099,387	3.45E-10	Whole Panel	0.42	0.78%	15.85	16.35	High				
Linear Plasticity	Cr	1	18,056,466	9.34E-10	*Indica*	0.32	3.59%	17.81	18.31	High				
Linear Plasticity	Cr	2	16,307,502	4.32E-13	*Indica*	0.08	3.59%	16.25	16.69	High				
Linear Plasticity	Cr	4	2,542,125	9.28E-09	*Indica*	0.16	3.59%	2.44	2.64	High				
Linear Plasticity	Cr	4	14,599,445	9.15E-11	*Japonica*	0.08	12.97%	14.50	14.70	High				
Linear Plasticity	Cr	5	6,188,177	6.36E-12	Whole Panel	0.17	0.29%	6.05	6.36	Low				
Linear Plasticity	Cr	6	24,059,277	1.20E-09	Whole Panel	0.40	2.18%	23.81	24.31	Low				
Linear Plasticity	Cr	7	367,318	1.29E-11	Whole Panel	0.28	0.02%	0.12	0.62	High				

Phenotypic Measures	Elements	Chr	Pos	P.value (GWAS)	Panel	MAF	PVE (Phenotypic measures)	Significant Region Start(Mb)	Significant Region End(Mb)	Allele Effect (Nip)	Overlap (Between Panels)	Overlap (Between Measures)	Reference	Genes
Linear Plasticity	Cr	7	11,996,735	1.18E-12	Whole Panel	0.28	21.59%	11.75	12.25	Low	Y			
Linear Plasticity	Cr	7	11,997,353	9.34E-12	*Japonica*	0.37	12.97%	11.75	12.25	Low	Y			
Linear Plasticity	Cr	9	2,275,721	4.52E-09	*Indica*	0.15	0.24%	2.18	2.38	High				
Linear Plasticity	Cr	10	8,553,984	7.77E-09	*Indica*	0.11	3.59%	8.45	8.65	High				
Linear Plasticity	Cr	12	23,375,323	1.22E-08	Whole Panel	0.09	0.60%	23.31	23.51	Low				
Linear Plasticity	Cu	1	27,503,457	8.89E-09	Whole Panel	0.50	2.11%	27.25	27.75	Low			d,m	
Linear Plasticity	Cu	2	13,521,478	2.06E-09	*Japonica*	0.41	4.04%	13.27	13.77	Low				
Linear Plasticity	Cu	5	6,002,352	2.73E-09	*Japonica*	0.10	1.29%	5.75	6.25	Low				
Linear Plasticity	Cu	5	6,538,008	1.44E-10	Whole Panel	0.10	12.35%	6.34	6.56	Low				
Linear Plasticity	Cu	6	11,184,487	4.41E-11	*Japonica*	0.09	1.33%	11.13	11.54	Low			k,m	
Linear Plasticity	Cu	9	6,640,156	5.19E-10	Whole Panel	0.27	14.53%	6.58	6.78	High				
Linear Plasticity	Cu	9	9,787,327	2.12E-09	*Japonica*	0.05	12.00%	9.54	9.79	High				
Linear Plasticity	Fe	1	4,957,933	7.18E-09	Whole Panel	0.43	1.76%	4.75	4.98	Low				
Linear Plasticity	Fe	2	32,978,883	2.10E-14	Whole Panel	0.39	0.07%	32.73	33.23	High				
Linear Plasticity	Fe	4	26,495,979	2.31E-10	Whole Panel	0.25	15.07%	26.25	26.75	Low				
Linear Plasticity	Fe	6	10,460,265	9.16E-09	*Indica*	0.15	7.92%	10.21	10.71	Low			q	
Linear Plasticity	K	1	21,861,259	1.41E-13	Whole Panel	0.08	0.92%	21.61	22.11	Low			b	
Linear Plasticity	K	1	33,555,927	3.80E-15	Whole Panel	0.14	17.96%	33.46	33.66	Low	Y			
Linear Plasticity	K	1	33,555,927	7.75E-19	*Japonica*	0.29	7.29%	33.46	33.66	Low	Y			
Linear Plasticity	K	1	42,574,579	8.86E-09	*Indica*	0.07	12.62%	42.56	42.86	Low				
Linear Plasticity	K	2	24,633,260	5.95E-10	Whole Panel	0.25	0.04%	24.52	24.72	Low			n,r	
Linear Plasticity	K	5	14,061,514	5.51E-09	Whole Panel	0.09	2.29%	13.81	14.31	Low				
Linear Plasticity	K	7	17,406,472	4.97E-10	Whole Panel	0.28	7.29%	17.30	17.50	High				
Linear Plasticity	K	8	15,800,282	1.15E-08	*Indica*	0.11	11.38%	15.70	15.90	High			b	
Linear Plasticity	K	8	21,211,932	6.68E-11	Whole Panel	0.05	7.29%	21.15	21.35	High				
Linear Plasticity	K	9	10,851,557	1.79E-08	*Japonica*	0.24	8.89%	10.53	11.02	High			r	
Linear Plasticity	K	11	19,598,306	4.47E-09	Whole Panel	0.09	7.29%	19.58	19.89	High				
Linear Plasticity	K	11	22,313,144	2.29E-08	*Japonica*	0.15	5.84%	22.21	22.41	Low				
Linear Plasticity	Mg	1	33,555,927	4.36E-12	Whole Panel	0.14	9.74%	33.46	33.66	Low			k	
Linear Plasticity	Mg	2	35,267,878	7.25E-10	Whole Panel	0.42	9.74%	35.16	35.36	High			r	
Linear Plasticity	Mg	4	814,308	5.57E-11	Whole Panel	0.11	9.74%	0.56	1.06	Low				
Linear Plasticity	Mg	4	16,008,180	7.58E-11	Whole Panel	0.08	5.89%	15.76	16.26	Low			k	

Phenotypic Measures	Elements	Chr	Pos	P.value (GWAS)	Panel	MAF	PVE (Phenotypic measures)	Significant Region Start(Mb)	Significant Region End(Mb)	Allele Effect (Nip)	Overlap (Between Panels)	Overlap (Between Measures)	Reference	Genes
Linear Plasticity	Mg	4	35,057,073	1.89E-10	Whole Panel	0.09	9.74%	34.94	35.14	Low				
Linear Plasticity	Mg	7	29,065,923	6.64E-09	*Japonica*	0.43	13.97%	29.05	29.25	High		Y	r	
Linear Plasticity	Mn	1	6,197,843	1.49E-08	*Indica*	0.26	15.31%	5.95	6.45	Low			d,i,p	
Linear Plasticity	Mn	3	5,797,006	9.93E-13	*Japonica*	0.18	17.03%	5.55	5.99	High		Y	d,r	
Linear Plasticity	Mn	3	6,719,733	7.80E-10	Whole Panel	0.17	7.54%	6.57	6.77	High			i	*MTP8.1*
Linear Plasticity	Mn	6	6,637,497	3.40E-11	Whole Panel	0.05	4.19%	6.64	7.11	Low				
Linear Plasticity	Mn	7	8,874,894	7.11E-14	Whole Panel	0.19	7.54%	8.62	9.12	Low			d,i	*NRAMP5*
Linear Plasticity	Mn	10	18,352,030	2.30E-09	Whole Panel	0.16	7.54%	18.28	18.48	High			b,k	
Linear Plasticity	Mn	11	3,351,208	6.49E-11	Whole Panel	0.10	1.37%	3.29	3.49	High				
Linear Plasticity	Mn	11	19,445,868	4.57E-10	*Japonica*	0.30	14.24%	19.43	19.92	High			r	
Linear Plasticity	Mn	11	21,424,624	2.69E-16	Whole Panel	0.05	1.96%	21.32	21.52	High			k,r	
Linear Plasticity	Mn	11	25,881,927	9.08E-09	Whole Panel	0.40	7.54%	25.59	25.88	Low			k,o	
Linear Plasticity	Mn	12	21,171,270	7.28E-09	*Japonica*	0.30	1.41%	20.92	21.42	Low				
Linear Plasticity	Mn	12	24,803,594	1.45E-09	Whole Panel	0.47	7.54%	24.55	25.05	Low			r	
Linear Plasticity	Na	7	1,187,203	5.29E-08	*Japonica*	0.17	11.02%	1.11	1.31	High				
Linear Plasticity	Na	8	144,296	1.78E-08	Whole Panel	0.09	15.83%	0.04	0.24	Low				
Linear Plasticity	Ni	1	33,555,927	6.46E-18	Whole Panel	0.14	1.69%	33.46	33.66	High	Y			
Linear Plasticity	Ni	1	33,555,927	5.02E-16	*Japonica*	0.29	1.53%	33.46	33.66	High	Y			
Linear Plasticity	Ni	1	36,798,732	8.36E-11	Whole Panel	0.06	12.13%	36.49	36.94	High				
Linear Plasticity	Ni	4	32,456,653	7.43E-09	*Japonica*	0.09	0.42%	32.21	32.71	Low				
Linear Plasticity	Ni	5	27,954,217	3.66E-10	Whole Panel	0.35	13.15%	27.87	28.07	High				
Linear Plasticity	Ni	6	3,019,092	5.48E-09	Whole Panel	0.07	2.01%	2.92	3.12	High				
Linear Plasticity	Ni	7	2,931,127	4.57E-09	*Japonica*	0.25	1.69%	2.77	2.97	High				
Linear Plasticity	Ni	8	15,976,418	1.11E-10	Whole Panel	0.39	15.34%	15.73	16.23	High				
Linear Plasticity	Ni	10	8,856,540	5.20E-10	Whole Panel	0.09	11.18%	8.76	8.96	High				
Linear Plasticity	Ni	11	3,574,206	1.27E-08	*Indica*	0.34	15.99%	3.45	3.65	High				
Linear Plasticity	Ni	11	20,808,842	9.07E-09	*Japonica*	0.07	1.69%	20.64	21.10	High				
Linear Plasticity	Ni	11	28,829,649	6.62E-09	Whole Panel	0.43	2.41%	28.73	28.93	High				
Linear Plasticity	Ni	12	1,254,782	4.49E-10	Whole Panel	0.11	2.61%	0.93	1.33	Low				
Linear Plasticity	P	1	27,797,186	4.12E-08	*Japonica*	0.05	16.60%	27.70	27.90	Low			b	
Linear Plasticity	P	5	20,758,645	7.46E-11	Whole Panel	0.45	16.69%	20.51	21.01	High			k	
Linear Plasticity	P	7	6,889,146	6.57E-12	Whole Panel	0.30	6.61%	6.64	7.14	High				

Phenotypic Measures	Elements	Chr	Pos	P.value (GWAS)	Panel	MAF	PVE (Phenotypic measures)	Significant Region Start(Mb)	Significant Region End(Mb)	Allele Effect (Nip)	Overlap (Between Panels)	Overlap (Between Measures)	Reference	Genes
Linear Plasticity	P	8	673,949	2.20E-08	*Indica*	0.40	18.90%	0.56	0.76	High				
Linear Plasticity	P	11	10,286,298	4.88E-11	Whole Panel	0.10	14.01%	10.19	10.39	Low				
Linear Plasticity	P	11	28,350,406	8.16E-10	Whole Panel	0.47	0.76%	28.10	28.60	Low		Y		
Linear Plasticity	S	8	10,533,717	8.20E-09	Whole Panel	0.48	4.50%	10.28	10.78	High				
Linear Plasticity	S	10	8,436,666	8.96E-12	*Japonica*	0.09	12.15%	8.34	8.54	High				
Linear Plasticity	S	10	10,417,578	8.54E-09	*Japonica*	0.36	13.52%	10.40	10.76	Low				
Linear Plasticity	S	11	5,853,198	1.92E-11	Whole Panel	0.49	4.50%	5.60	6.10	High				
Linear Plasticity	S	11	26,344,588	1.07E-08	*Indica*	0.43	12.56%	26.09	26.59	Low	Y			
Linear Plasticity	S	11	26,344,588	8.82E-11	Whole Panel	0.24	4.50%	26.09	26.59	Low	Y			
Linear Plasticity	Se	4	15,899,991	4.58E-09	Whole Panel	0.08	15.82%	15.65	16.15	High				
Linear Plasticity	Zn	1	29,339,007	1.47E-10	Whole Panel	0.06	3.24%	29.25	29.45	High				
Linear Plasticity	Zn	1	32,235,498	3.29E-08	*Japonica*	0.13	0.00%	31.99	32.49	Low			o,p	
Linear Plasticity	Zn	2	35,271,642	1.10E-08	*Japonica*	0.08	18.51%	35.17	35.37	High			m	
Linear Plasticity	Zn	3	6,391,925	1.47E-10	*Indica*	0.28	2.39%	6.14	6.64	Low				
Linear Plasticity	Zn	4	29,338,989	2.61E-09	*Indica*	0.08	4.35%	29.09	29.59	Low			e,j,r	
Linear Plasticity	Zn	5	28,279,181	9.19E-09	*Indica*	0.13	4.35%	28.18	28.38	High			o	
Linear Plasticity	Zn	6	10,428,696	6.26E-12	*Indica*	0.07	4.35%	10.10	10.59	Low			n,p	
Linear Plasticity	Zn	6	14,156,952	2.33E-13	Whole Panel	0.13	11.74%	13.91	14.41	High				
Linear Plasticity	Zn	7	1,186,758	3.61E-09	Whole Panel	0.08	11.52%	1.11	1.31	High				
Linear Plasticity	Zn	8	2,795,270	3.06E-12	*Japonica*	0.47	19.30%	2.72	2.92	Low	Y	Y	b	
Linear Plasticity	Zn	8	2,802,062	4.77E-11	Whole Panel	0.21	14.29%	2.70	2.90	Low	Y	Y		
Linear Plasticity	Zn	8	16,367,622	2.30E-09	*Indica*	0.07	4.35%	16.27	16.47	Low				
Linear Plasticity	Zn	9	7,938,546	5.72E-11	*Indica*	0.43	4.35%	7.69	8.19	High			q	
Linear Plasticity	Zn	9	18,056,014	3.25E-09	Whole Panel	0.12	2.81%	17.81	18.31	Low			e,m	
Linear Plasticity	Zn	10	9,561,158	1.18E-13	Whole Panel	0.36	12.15%	9.31	9.81	Low			m,r	
Linear Plasticity	Zn	12	15,229,642	1.58E-09	*Indica*	0.17	4.35%	15.13	15.33	High				
Non-linear Plasticity	As	1	3,409,528	1.04E-09	*Indica*	0.05	6.31%	3.31	3.51	High				
Non-linear Plasticity	As	2	32,166,813	1.02E-10	Whole Panel	0.44	17.78%	31.92	32.42	High				
Non-linear Plasticity	As	4	1,508,827	3.58E-08	*Japonica*	0.10	20.62%	1.41	1.61	Low				
Non-linear Plasticity	As	5	27,009,168	2.24E-09	*Indica*	0.33	6.31%	26.91	27.11	Low				
Non-linear Plasticity	As	9	12,571,156	1.44E-13	*Indica*	0.05	6.31%	12.33	12.58	High				
Non-linear Plasticity	As	10	9,742,336	1.59E-10	*Japonica*	0.17	20.62%	9.64	9.84	Low				

Phenotypic Measures	Elements	Chr	Pos	P.value (GWAS)	Panel	MAF	PVE (Phenotypic measures)	Significant Region Start(Mb)	Significant Region End(Mb)	Allele Effect (Nip)	Overlap (Between Panels)	Overlap (Between Measures)	Reference	Genes
Non-linear Plasticity	As	12	2,502,754	1.57E-08	*Indica*	0.11	6.31%	2.40	2.60	High			m	
Non-linear Plasticity	Cd	3	35,788,257	7.72E-11	Whole Panel	0.09	18.49%	35.61	35.81	Low			e,q	
Non-linear Plasticity	Cd	4	1,727,453	9.12E-09	Whole Panel	0.05	0.78%	1.63	1.83	High			q	
Non-linear Plasticity	Cd	7	18,453,163	6.32E-12	Whole Panel	0.20	1.54%	18.35	18.55	Low			q	
Non-linear Plasticity	Cd	8	20,825,168	1.36E-09	Whole Panel	0.10	17.53%	20.75	20.95	Low				
Non-linear Plasticity	Cd	9	14,446,563	1.50E-11	Whole Panel	0.06	13.98%	14.20	14.70	High				
Non-linear Plasticity	Cr	2	15,068,277	1.75E-20	*Japonica*	0.10	11.06%	14.97	15.17	Low				
Non-linear Plasticity	Cr	8	3,776,609	4.37E-08	*Japonica*	0.06	6.76%	3.68	3.88	Low				
Non-linear Plasticity	Cr	10	10,100,239	7.98E-10	*Japonica*	0.07	11.06%	9.85	10.35	Low				
Non-linear Plasticity	Cr	11	28,140,687	8.95E-10	*Japonica*	0.06	11.06%	27.99	28.43	Low				
Non-linear Plasticity	Cu	6	9,395,827	1.75E-08	Whole Panel	0.37	4.64%	9.15	9.65	High			k	
Non-linear Plasticity	Fe	1	35,512,030	7.27E-11	*Japonica*	0.08	0.28%	35.41	35.61	Low			i,l	
Non-linear Plasticity	Fe	10	13,745,390	9.40E-09	Whole Panel	0.44	2.42%	13.50	14.00	Low			c	
Non-linear Plasticity	Mg	3	34,050,852	5.52E-10	Whole Panel	0.10	8.44%	33.95	34.15	Low				
Non-linear Plasticity	Mg	7	29,177,987	7.14E-11	Whole Panel	0.33	18.70%	28.93	29.43	Low		Y	r	
Non-linear Plasticity	Mg	8	6,816,038	1.09E-11	Whole Panel	0.22	12.30%	6.57	7.07	Low				
Non-linear Plasticity	Mg	11	27,222,144	1.56E-13	Whole Panel	0.07	15.22%	27.22	27.65	Low				
Non-linear Plasticity	Mn	1	23,617,899	1.08E-08	Whole Panel	0.32	3.56%	23.37	23.87	High			b	
Non-linear Plasticity	Mn	1	35,512,030	7.09E-11	*Japonica*	0.08	3.53%	35.41	35.61	Low				
Non-linear Plasticity	Mn	2	22,432,713	7.09E-09	*Japonica*	0.26	3.53%	22.30	22.57	Low				
Non-linear Plasticity	Mn	3	5,824,894	6.72E-10	Whole Panel	0.09	9.99%	5.57	6.07	High		Y	d,r	
Non-linear Plasticity	Mn	5	9,474,966	9.58E-09	*Japonica*	0.07	3.53%	9.37	9.57	Low				
Non-linear Plasticity	Mn	10	4,402,667	3.99E-09	Whole Panel	0.09	9.99%	4.30	4.50	Low				
Non-linear Plasticity	Ni	1	9,677,594	1.81E-10	Whole Panel	0.17	6.50%	9.58	9.78	High				
Non-linear Plasticity	Ni	1	23,612,824	7.08E-11	*Japonica*	0.18	12.08%	23.45	23.65	High				
Non-linear Plasticity	Ni	1	31,532,723	9.24E-10	Whole Panel	0.11	6.50%	31.06	31.53	Low				
Non-linear Plasticity	Ni	2	31,732,068	5.96E-09	Whole Panel	0.13	1.55%	31.48	31.98	High				
Non-linear Plasticity	Ni	4	1,792,324	8.16E-11	Whole Panel	0.31	0.29%	1.54	2.04	Low			k	
Non-linear Plasticity	Ni	6	10,393,703	1.17E-08	Whole Panel	0.15	1.47%	10.14	10.64	Low				
Non-linear Plasticity	Ni	6	15,433,153	2.37E-10	Whole Panel	0.19	11.80%	15.33	15.53	High	Y			
Non-linear Plasticity	Ni	6	15,433,153	3.28E-08	*Japonica*	0.42	6.50%	15.33	15.53	High	Y			
Non-linear Plasticity	Ni	7	20,715,732	4.93E-09	Whole Panel	0.26	5.47%	20.61	20.81	High				

Phenotypic Measures	Elements	Chr	Pos	P.value (GWAS)	Panel	MAF	PVE (Phenotypic measures)	Significant Region Start(Mb)	Significant Region End(Mb)	Allele Effect (Nip)	Overlap (Between Panels)	Overlap (Between Measures)	Reference	Genes
Non-linear Plasticity	Ni	8	2,372,677	1.05E-12	Whole Panel	0.05	4.27%	2.37	2.86	Low				
Non-linear Plasticity	Ni	10	148,287	3.76E-09	*Japonica*	0.20	2.12%	-0.10	0.40	Low				
Non-linear Plasticity	Ni	10	5,974,487	5.29E-09	Whole Panel	0.08	1.32%	5.87	6.07	High				
Non-linear Plasticity	Ni	11	13,985,661	6.18E-10	Whole Panel	0.24	6.03%	13.74	14.24	Low				
Non-linear Plasticity	Ni	12	27,085,898	3.60E-10	Whole Panel	0.11	6.50%	26.95	27.18	High				
Non-linear Plasticity	Pb	4	34,431,554	1.19E-08	Whole Panel	0.48	16.63%	34.18	34.68	High				
Non-linear Plasticity	Pb	7	1,482,945	3.15E-08	*Japonica*	0.07	14.96%	1.38	1.58	Low				
Non-linear Plasticity	Pb	12	6,759,531	5.60E-09	*Japonica*	0.06	7.69%	6.51	7.01	Low				
Non-linear Plasticity	Pb	12	26,966,856	2.39E-08	*Japonica*	0.14	14.96%	26.72	27.22	High				
Non-linear Plasticity	S	11	19,023,378	1.05E-08	Whole Panel	0.33	4.57%	18.77	19.27	High				
Non-linear Plasticity	Se	1	35,512,030	1.23E-09	*Japonica*	0.08	1.69%	35.41	35.61	Low				
Non-linear Plasticity	Se	4	14,599,445	7.99E-15	*Japonica*	0.08	1.69%	14.50	14.70	Low				
Non-linear Plasticity	Se	4	28,351,497	1.09E-08	*Japonica*	0.08	1.69%	28.25	28.45	Low				
Non-linear Plasticity	Se	5	21,167,548	3.25E-08	*Japonica*	0.13	1.69%	21.07	21.27	Low				
Non-linear Plasticity	Se	6	31,051,366	3.01E-09	*Japonica*	0.05	1.69%	30.95	31.15	Low				
Non-linear Plasticity	Se	7	27,008,817	4.19E-09	*Indica*	0.21	3.89%	26.78	27.03	Low			k	
Non-linear Plasticity	Se	8	5,371,023	3.06E-09	*Japonica*	0.13	0.50%	5.12	5.62	Low				
Non-linear Plasticity	Se	8	11,541,758	5.22E-10	Whole Panel	0.40	16.25%	11.29	11.79	High	Y			
Non-linear Plasticity	Se	8	11,541,758	2.31E-10	*Indica*	0.07	7.12%	11.44	11.64	High	Y			
Non-linear Plasticity	Se	8	13,431,441	4.07E-09	Whole Panel	0.05	11.19%	13.18	13.68	Low				
Non-linear Plasticity	Se	9	4,689,995	1.96E-09	Whole Panel	0.16	3.69%	4.44	4.94	High				
Non-linear Plasticity	Se	11	19,242,926	6.45E-11	*Japonica*	0.24	1.69%	18.99	19.33	High				
Non-linear Plasticity	Se	11	27,829,933	1.37E-08	Whole Panel	0.13	11.19%	27.58	28.08	Low			q	
Non-linear Plasticity	Zn	1	25,243,883	2.56E-10	Whole Panel	0.13	8.13%	25.11	25.31	Low				
Non-linear Plasticity	Zn	1	30,912,861	8.83E-09	*Indica*	0.46	13.79%	30.66	31.16	Low				
Non-linear Plasticity	Zn	3	14,148,910	2.00E-13	Whole Panel	0.43	4.20%	13.90	14.40	Low				
Non-linear Plasticity	Zn	4	9,743,667	7.28E-09	Whole Panel	0.09	9.82%	9.49	9.99	Low				
Non-linear Plasticity	Zn	6	23,710,865	8.94E-10	Whole Panel	0.10	1.54%	23.54	23.85	High			o,q	
Non-linear Plasticity	Zn	7	24,402,410	8.05E-13	Whole Panel	0.08	3.23%	24.15	24.65	Low			e,i,r	
Non-linear Plasticity	Zn	8	2,422,420	1.08E-09	Whole Panel	0.11	19.32%	2.42	2.92	Low		Y	b	
Non-linear Plasticity	Zn	8	21,935,326	2.17E-15	Whole Panel	0.05	1.92%	21.69	22.19	Low			d	
Non-linear Plasticity	Zn	10	2,147,156	2.37E-10	Whole Panel	0.07	7.28%	2.13	2.36	Low			r	

a: Abe T, Taguchi-Shiobara F, Kojima Y, Ebitani T, Kuramata M, Yamamoto T, Yano M, Ishikawa S (2011) Detection of a QTL for accumulating Cd in rice that enables efficient Cd phytoextraction from soil. Breeding Science 61:43-51

b: Descalsota-Empleo GI, Amparado A, Inabangan-Asilo MA, Tesoro F, Stangoulis J, Reinke R, Swamy BPM (2019) Genetic mapping of QTL for agronomic traits and grain mineral elements in rice. The Crop Journal 7:560-572

c: Descalsota GIL, Swamy BPM, Zaw H, Inabangan-Asilo MA, Amparado A, Mauleon R, Chadha-Mohanty P, Arocena EC, Raghavan C, Leung H, Hernandez JE, Lalusin AB, Mendioro MS, Diaz MGQ, Reinke R (2018) Genome-Wide Association Mapping in a Rice MAGIC Plus Population Detects QTLs and Genes Useful for

d: Garcia-Oliveira AL, Tan L, Fu Y, Sun C (2009) Genetic identification of quantitative trait loci for contents of mineral nutrients in rice grain. Journal of integrative plant biology 51:84-92

e: Huang Y, Sun C, Min J, Chen Y, Tong C, Bao J (2015) Association Mapping of Quantitative Trait Loci for Mineral Element Contents in Whole Grain Rice (Oryza sativa L.). Journal of Agricultural and Food Chemistry 63:10885-10892

f: Huang Y, Tong C, Xu F, Chen Y, Zhang C, Bao J (2016) Variation in mineral elements in grains of 20 brown rice accessions in two environments. Food chemistry 192:873-878

g: Ishikawa S, Abe T, Kuramata M, Yamaguchi M, Ando T, Yamamoto T, Yano M (2010) A major quantitative trait locus for increasing cadmium-specific concentration in rice grain is located on the short arm of Somosome 7. Journal of Experimental Botany 61:923-934

h: Kashiwagi T, Shindoh K, Hirotsu N, Ishimaru K (2009) Evidence for separate translocation pathways in determining cadmium accumulation in grain and aerial plant parts in rice. BMC Plant Biology 9:8

i: Lu K, Li L, Zheng X, Zhang Z, Mou T, Hu Z (2008) Quantitative trait loci controlling Cu, Ca, Zn, Mn and Fe content in rice grains. Journal of Genetics 87:305-310

j: Nawaz Z, Kakar KU, Li XB, Li S, Zhang B, Shou HX, Shu QY (2015) Genome-wide Association Mapping of Quantitative Trait Loci (QTLs) for Contents of Eight

k: Norton GJ, Deacon CM, Xiong LZ, Huang SY, Meharg AA, Price AH (2010) Genetic mapping of the rice ionome in leaves and grain: identification of QTLs for 17

l: Norton GJ, Douglas A, Lahner B, Yakubova E, Guerinot ML, Pinson SR, Tarpley L, Eizenga GC, McGrath SP, Zhao FJ, Islam MR, Islam S, Duan G, Zhu Y, Salt DE,

m: Norton GJ, Duan G-L, Lei M, Zhu Y-G, Meharg AA, Price AH (2012) Identification of quantitative trait loci for rice grain element composition on an arsenic impacted

n: Swamy BPM, Descalsota GIL, Nha CT, Amparado A, Inabangan-Asilo MA, Manito C, Tesoro F, Reinke R (2018) Identification of genomic regions associated with

o: Yang M, Lu K, Zhao FJ, Xie W, Ramakrishna P, Wang G, Du Q, Liang L, Sun C, Zhao H, Zhang Z, Liu Z, Tian J, Huang XY, Wang W, Dong H, Hu J, Ming L, Xing Y,

p: Yu YH, Shao YF, Liu J, Fan YY, Sun CX, Cao ZY, Zhuang JY (2015) Mapping of Quantitative Trait Loci for Contents of Macro- and Microelements in Milled Rice

q: Zhang GM, Zheng TQ, Chen Z, Wang YL, Wang Y, Shi YM, Wang CC, Zhang LY, Ma JT, Deng LW, Li W, Xu TT, Liang CZ, Xu JL, Li ZK (2018) Joint Exploration of

r: Zhang M, Pinson SR, Tarpley L, Huang XY, Lahner B, Yakubova E, Baxter I, Guerinot ML, Salt DE (2014) Mapping and validation of quantitative trait loci associated

Table S6 The results of whole-genome regression in each environment

Elements	Type	2014Field-1	2014Field-2	2015Field-1	2015Field-2	2016Field-1	2016Field-2	2016Field-3	2017Field-1	Mean
As	mSAL	NA[a]	NA	NA	NA	NA	NA	NA	NA	NaN[b]
As	lSAL	NA	NA	NA	NA	NA	NA	NA	NA	NaN
As	nlSAL	NA	NA	NA	0.95%	1.07%	1.09%	2.42%	2.99%	1.70%
Ca	mSAL	0.67%	0.33%	0.28%	0.25%	0.46%	2.38%	0.27%	NA	0.66%
Ca	lSAL	0.98%	1.04%	1.68%	0.42%	1.33%	0.58%	1.82%	NA	1.12%
Ca	nlSAL	NA	NA	NA	NA	NA	NA	NA	NA	NaN
Cd	mSAL	12.36%	1.13%	11.11%	1.64%	22.60%	4.78%	7.98%	1.24%	7.86%
Cd	lSAL	26.79%	31.57%	27.67%	2.03%	16.60%	33.25%	2.16%	5.48%	18.19%
Cd	nlSAL	0.93%	0.91%	0.40%	0.32%	1.64%	0.38%	0.30%	0.37%	0.65%
Cr	mSAL	NA	NA	NA	0.99%	0.23%	8.15%	1.09%	0.26%	2.14%
Cr	lSAL	NA	NA	NA	1.75%	1.11%	18.82%	1.06%	0.61%	4.67%
Cr	nlSAL	NA	NA	NA	0.48%	0.30%	6.43%	0.45%	0.29%	1.59%
Cu	mSAL	3.61%	20.71%	21.40%	1.92%	2.48%	13.89%	22.93%	2.19%	11.14%
Cu	lSAL	1.38%	2.15%	2.26%	14.38%	7.13%	2.79%	4.74%	0.61%	4.43%
Cu	nlSAL	NA	NA	NA	NA	NA	NA	NA	NA	NaN
Fe	mSAL	0.69%	3.29%	11.82%	11.13%	8.93%	10.24%	8.03%	11.82%	8.24%
Fe	lSAL	0.78%	4.01%	0.73%	1.07%	0.26%	0.31%	5.30%	0.30%	1.59%
Fe	nlSAL	0.14%	0.19%	0.18%	0.15%	0.14%	0.17%	0.16%	0.16%	0.16%
K	mSAL	NA	NA	NA	15.65%	13.19%	9.72%	17.26%	NA	13.95%
K	lSAL	NA	NA	NA	1.65%	2.74%	21.24%	4.68%	NA	7.58%
K	nlSAL	NA	NA	NA	NA	NA	NA	NA	NA	NaN
Mg	mSAL	24.31%	22.23%	30.44%	39.27%	32.51%	36.04%	39.88%	NA	32.10%
Mg	lSAL	0.62%	0.40%	0.34%	2.21%	0.69%	0.33%	1.21%	NA	0.83%
Mg	nlSAL	0.35%	0.28%	0.20%	0.26%	0.22%	0.21%	0.20%	NA	0.24%
Mn	mSAL	10.84%	10.74%	15.52%	3.38%	3.80%	6.61%	3.73%	22.10%	9.59%
Mn	lSAL	1.67%	2.16%	1.90%	34.72%	23.62%	23.92%	26.69%	5.03%	14.96%
Mn	nlSAL	0.39%	0.39%	0.34%	0.61%	0.62%	0.38%	0.40%	0.70%	0.48%
Na	mSAL	NA	NA	NA	NA	NA	NA	NA	NA	NaN
Na	lSAL	NA	NA	NA	2.23%	0.31%	0.58%	0.42%	NA	0.88%
Na	nlSAL	NA	NA	NA	NA	NA	NA	NA	NA	NaN
Ni	mSAL	0.60%	13.83%	0.51%	1.93%	0.40%	7.54%	1.54%	0.45%	3.35%
Ni	lSAL	39.39%	9.04%	26.95%	0.95%	12.56%	8.58%	0.81%	3.27%	12.69%
Ni	nlSAL	3.61%	0.91%	2.74%	0.66%	1.43%	3.88%	0.75%	0.76%	1.84%
P	mSAL	NA	NA	NA	15.52%	25.29%	18.91%	23.45%	NA	20.79%
P	lSAL	NA	NA	NA	8.71%	0.93%	2.77%	3.17%	NA	3.89%
P	nlSAL	NA	NA	NA	NA	NA	NA	NA	NA	NaN
Pb	mSAL	NA	NA	NA	NA	NA	NA	NA	NA	NaN
Pb	lSAL	NA	NA	NA	NA	NA	NA	NA	NA	NaN
Pb	nlSAL	0.79%	0.68%	2.19%	0.77%	0.80%	0.58%	1.19%	0.67%	0.96%
S	mSAL	NA	NA	NA	49.86%	38.92%	42.02%	35.56%	NA	41.59%
S	lSAL	NA	NA	NA	0.89%	1.73%	3.11%	1.09%	NA	1.70%
S	nlSAL	NA	NA	NA	NA	NA	NA	NA	NA	NaN
Se	mSAL	NA	NA	NA	NA	NA	NA	NA	NA	NaN
Se	lSAL	NA	NA	NA	NA	NA	NA	NA	NA	NaN

Elements	Type	2014Field-1	2014Field-2	2015Field-1	2015Field-2	2016Field-1	2016Field-2	2016Field-3	2017Field-1	Mean
Se	nlSAL	NA	NA	NA	3.08%	6.45%	3.33%	2.89%	2.76%	3.70%
Zn	mSAL	1.13%	1.21%	0.65%	16.85%	1.03%	5.10%	8.66%	16.17%	6.35%
Zn	lSAL	50.44%	56.07%	40.94%	12.39%	35.00%	37.63%	30.23%	2.19%	33.11%
Zn	nlSAL	1.76%	0.62%	0.89%	4.45%	0.76%	0.63%	1.53%	3.61%	1.78%

a: The proportion of phenotypic variance estimated in the whole-genome regression of each type of SAL, NA denotes the proportion of phenotypic variance was not estimated for insufficient quantity (less than 2).
b: The mean value was not estimated for insufficient quantity.

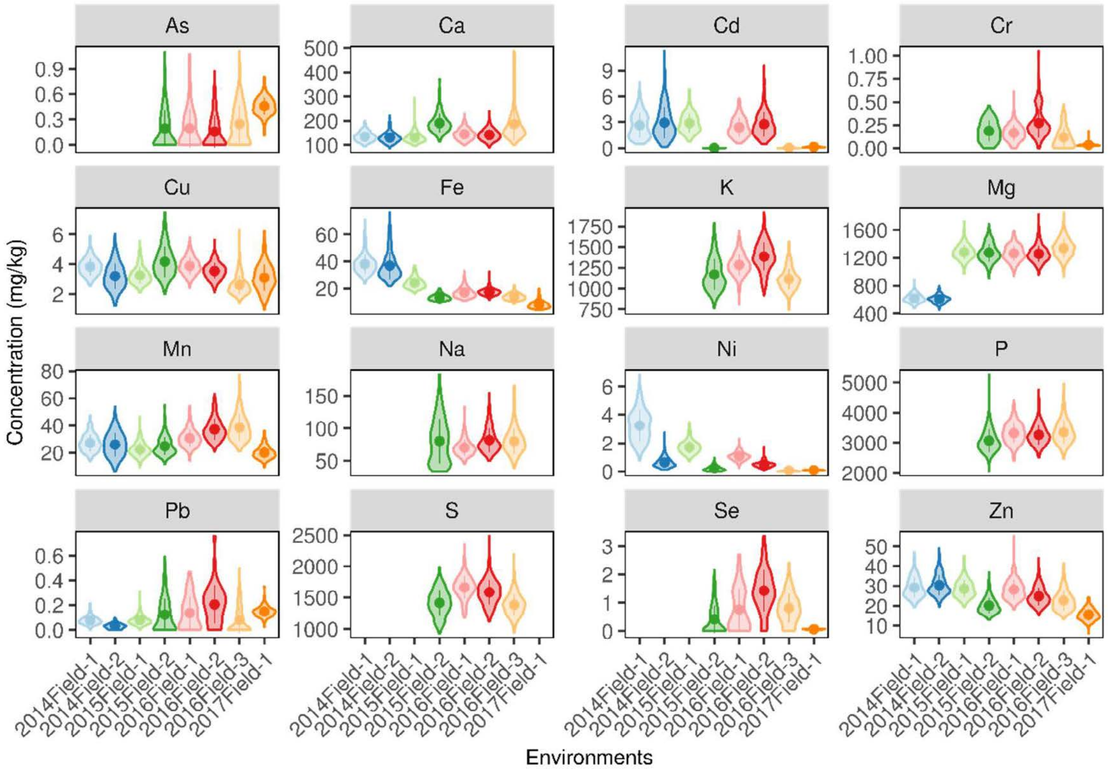

Fig. S 1 Violin plots for elemental concentrations among different environments. The dot and line range in each violin denotes the mean value and standard deviation respectively.

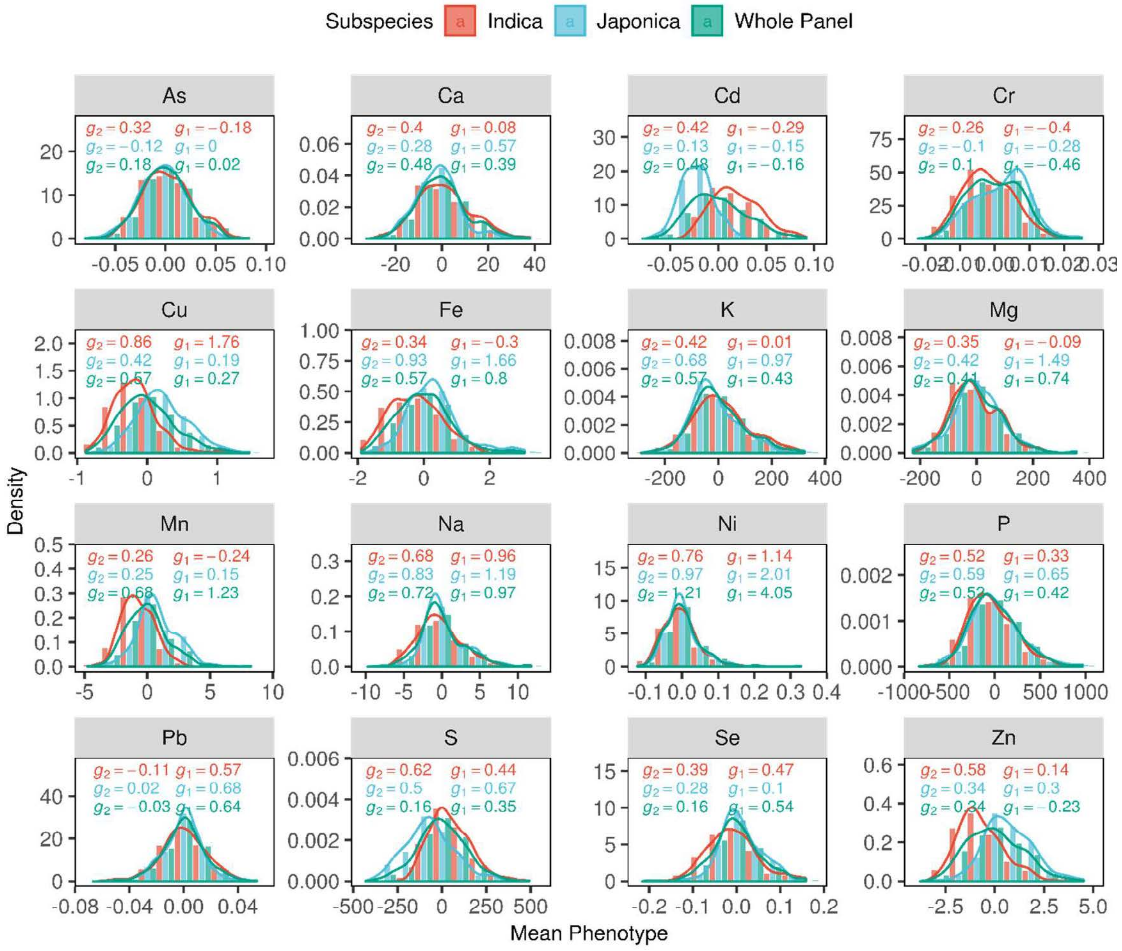

Fig. S 2 Histogram and kernel density of each element's mean phenotype in the whole panel and two subspecies. The skewness (g_1) and kurtosis (g_2) were labeled on each plot.

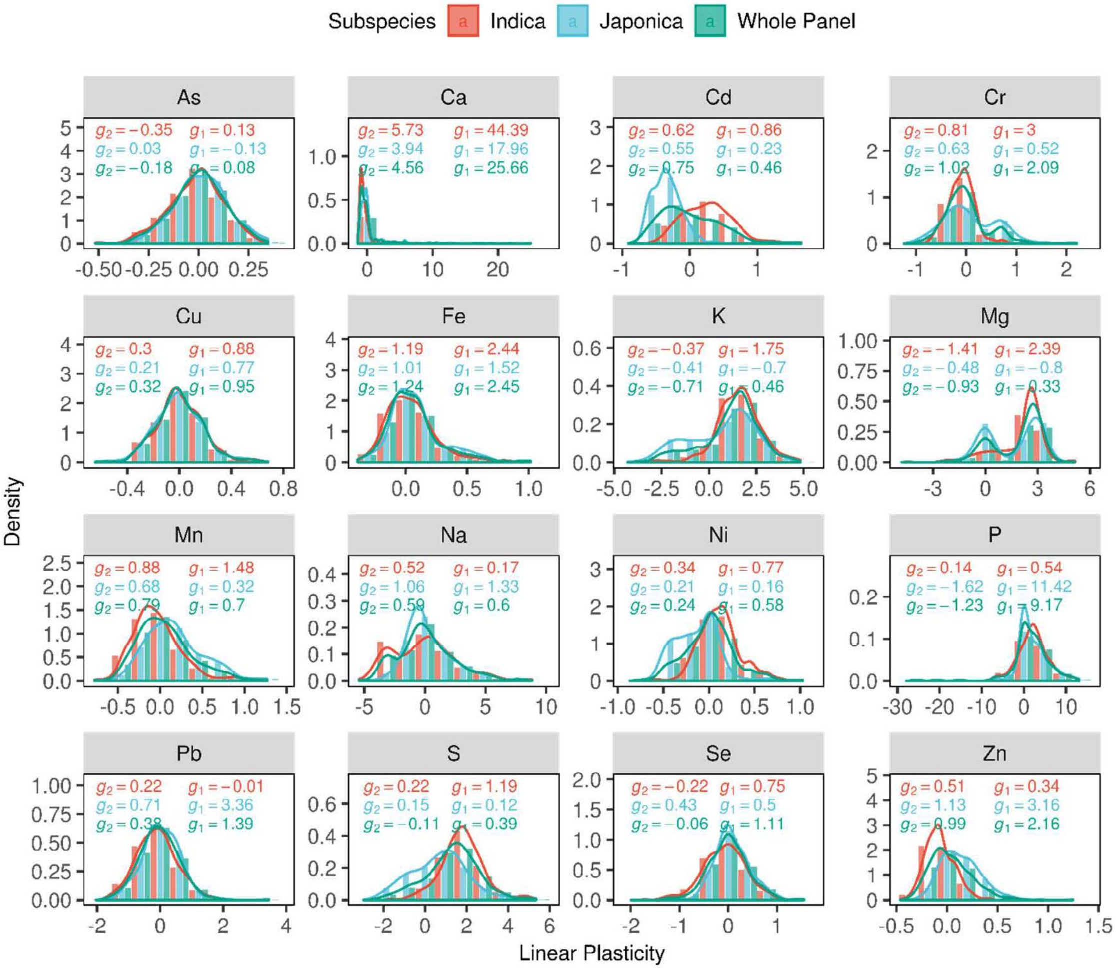

Fig. S 3 Histogram and kernel density of each element's linear plasticity in the whole panel and two subspecies. The skewness (g_1) and kurtosis (g_2) were labeled on each plot.

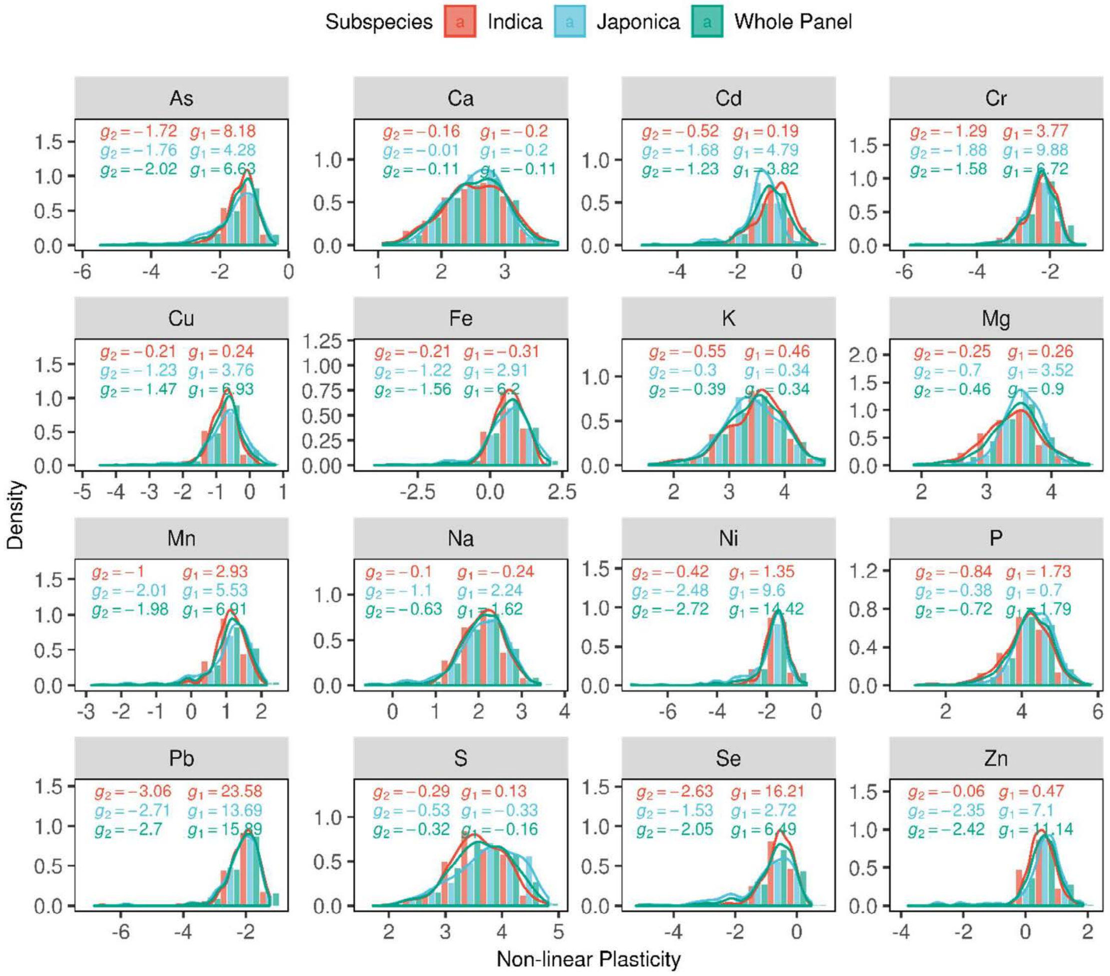

Fig. S 4 Histogram and kernel density of each element's non-linear plasticity in the whole panel and two subspecies. The skewness (g_1) and kurtosis (g_2) were labeled on each plot.

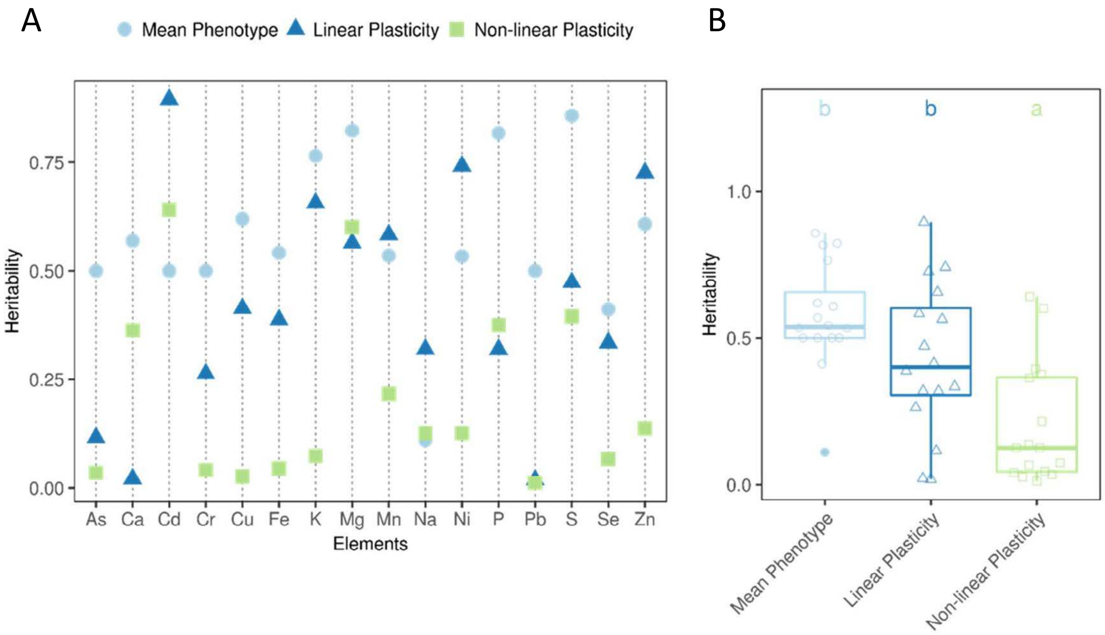

Fig. S 5 Marker-based heritability (h^2) of three phenotypic measures for each element. (A) The value of marker-based heritability. (B) The Boxplot of heritability of three phenotypic measures. The letter above each box indicates the multi-comparison results (Kruskal-Wallis, $P < 0.05$).

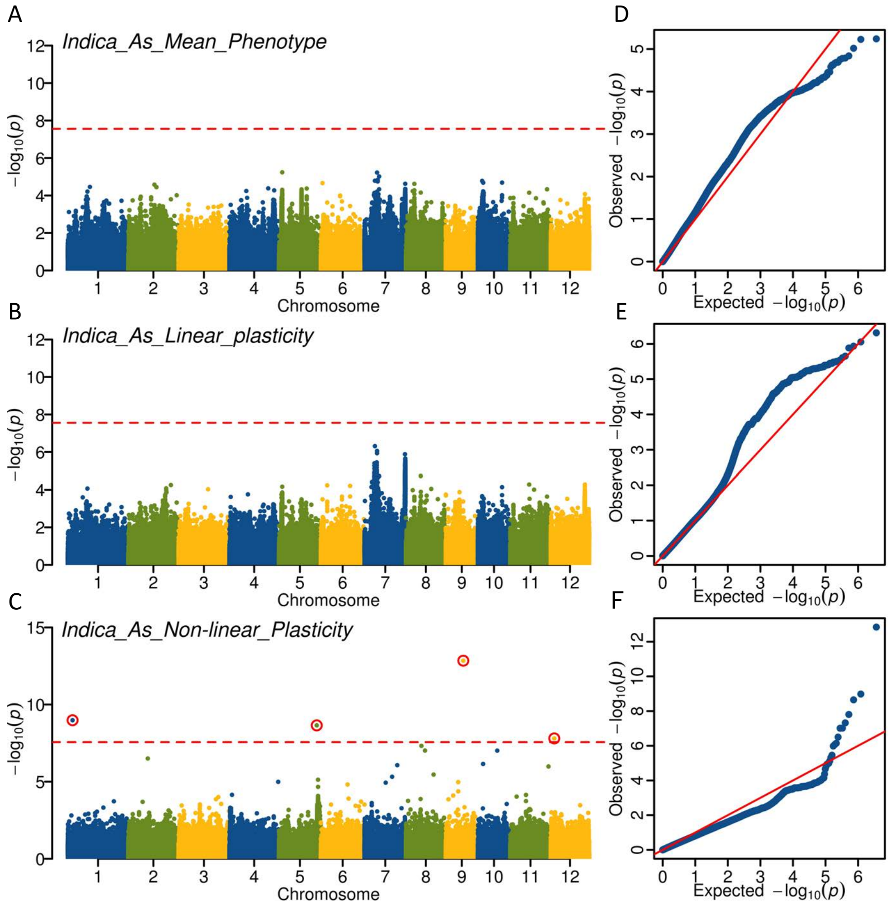

Fig. S 6 GWAS results of three phenotypic measures of As in the *Indica* subspecies. (A-C) Manhattan plots of (A) the mean phenotype, (B) the linear plasticity, and (C) the non-linear plasticity, the horizontal dashed line indicates the threshold at 2.7e-8. The significantly associated loci (SALs) were labeled with red circles. (D-F) Quantile-quantile plots of the mean phenotype (D), the linear plasticity (E), and the non-linear plasticity (F).

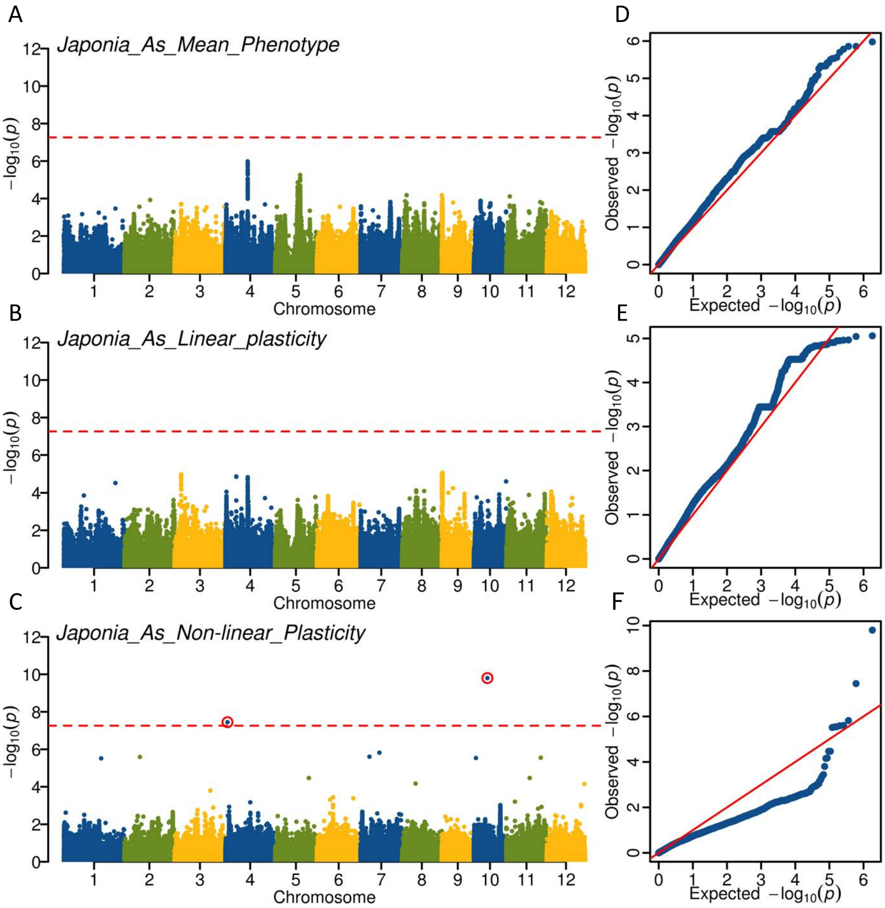

Fig. S 7 GWAS results of three phenotypic measures of As in the *Japonica* subspecies. (A-C) Manhattan plots of (A) the mean phenotype, (B) the linear plasticity, and (C) the non-linear plasticity, the horizontal dashed line indicates the threshold at 5.5e-8. The significantly associated loci (SALs) were labeled with red circles. (D-F) Quantile-quantile plots of the mean phenotype (D), the linear plasticity (E), and the non-linear plasticity (F).

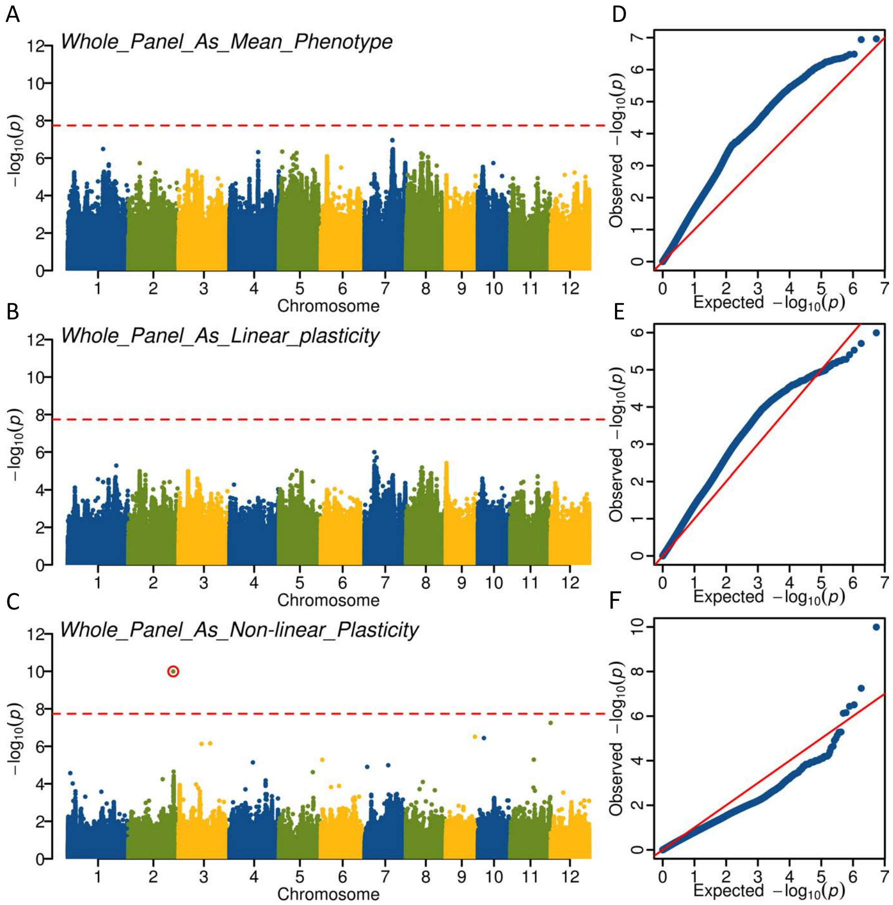

Fig. S 8 GWAS results of three phenotypic measures of As in the whole panel. (A-C) Manhattan plots of (A) the mean phenotype, (B) the linear plasticity, and (C) the non-linear plasticity, the horizontal dashed line indicates the threshold at 1.8e-8. The significantly associated loci (SALs) were labeled with red circles. (D-F) Quantile-quantile plots of the mean phenotype (D), the linear plasticity (E), and the non-linear plasticity (F).

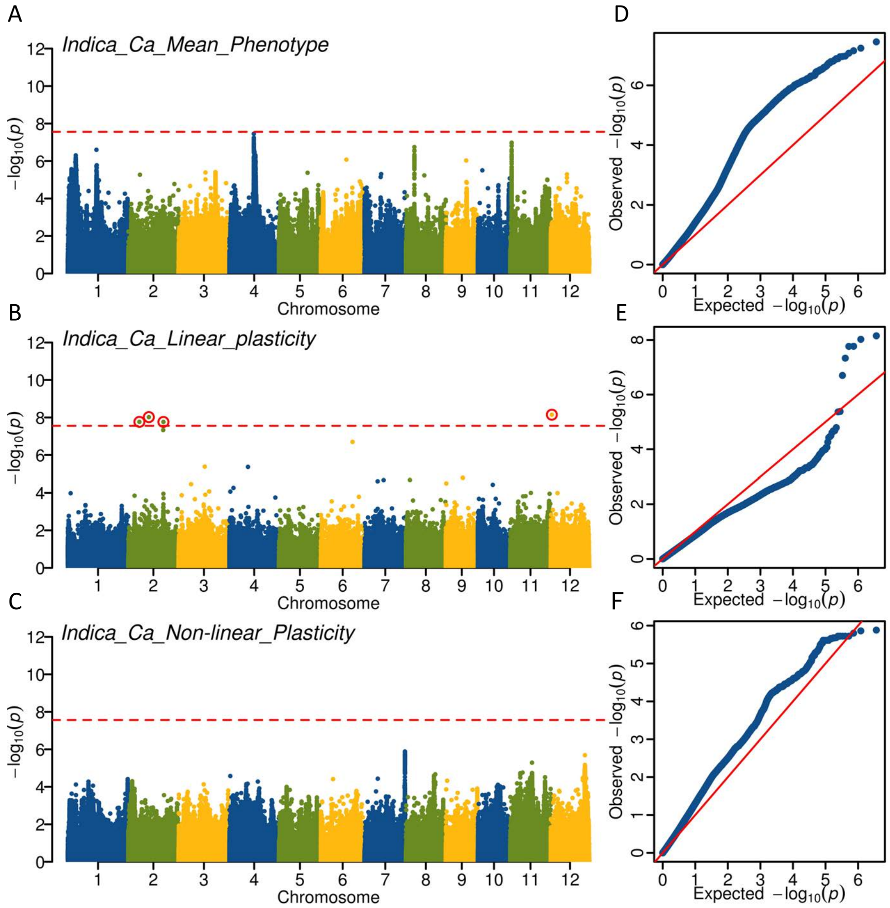

Fig. S 9 GWAS results of three phenotypic measures of Ca in the *Indica* subspecies. (A-C) Manhattan plots of (A) the mean phenotype, (B) the linear plasticity, and (C) the non-linear plasticity, the horizontal dashed line indicates the threshold at 2.7e-8. The significantly associated loci (SALs) were labeled with red circles. (D-F) Quantile-quantile plots of the mean phenotype (D), the linear plasticity (E), and the non-linear plasticity (F).

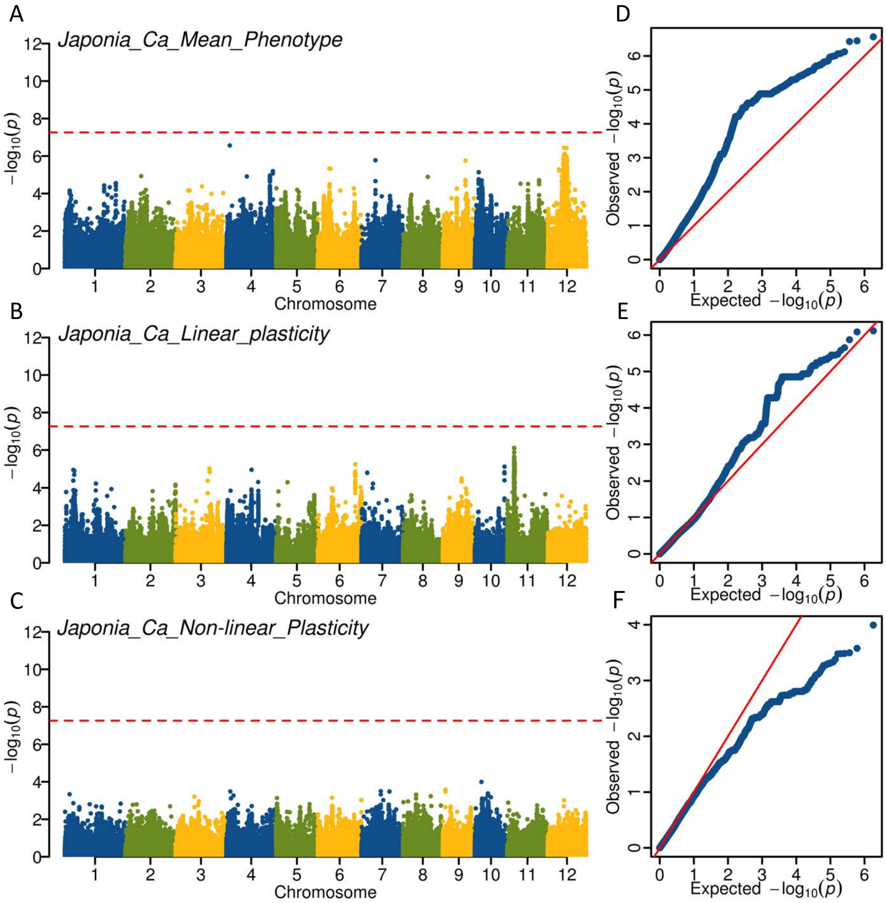

Fig. S 10 GWAS results of three phenotypic measures of Ca in the *Japonica* subspecies. (A-C) Manhattan plots of (A) the mean phenotype, (B) the linear plasticity, and (C) the non-linear plasticity, the horizontal dashed line indicates the threshold at 5.5e-8. The significantly associated loci (SALs) were labeled with red circles. (D-F) Quantile-quantile plots of the mean phenotype (D), the linear plasticity (E), and the non-linear plasticity (F).

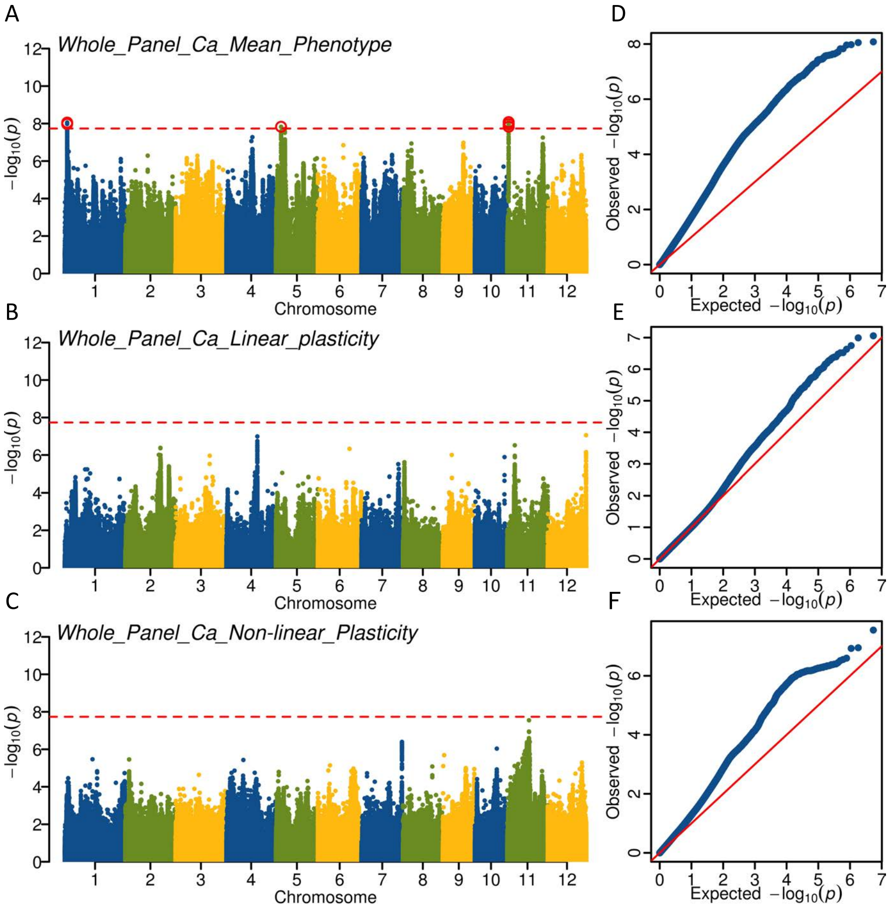

Fig. S 11 GWAS results of three phenotypic measures of Ca in the whole panel. (A-C) Manhattan plots of (A) the mean phenotype, (B) the linear plasticity, and (C) the non-linear plasticity, the horizontal dashed line indicates the threshold at 1.8e-8. The significantly associated loci (SALs) were labeled with red circles. (D-F) Quantile-quantile plots of the mean phenotype (D), the linear plasticity (E), and the non-linear plasticity (F).

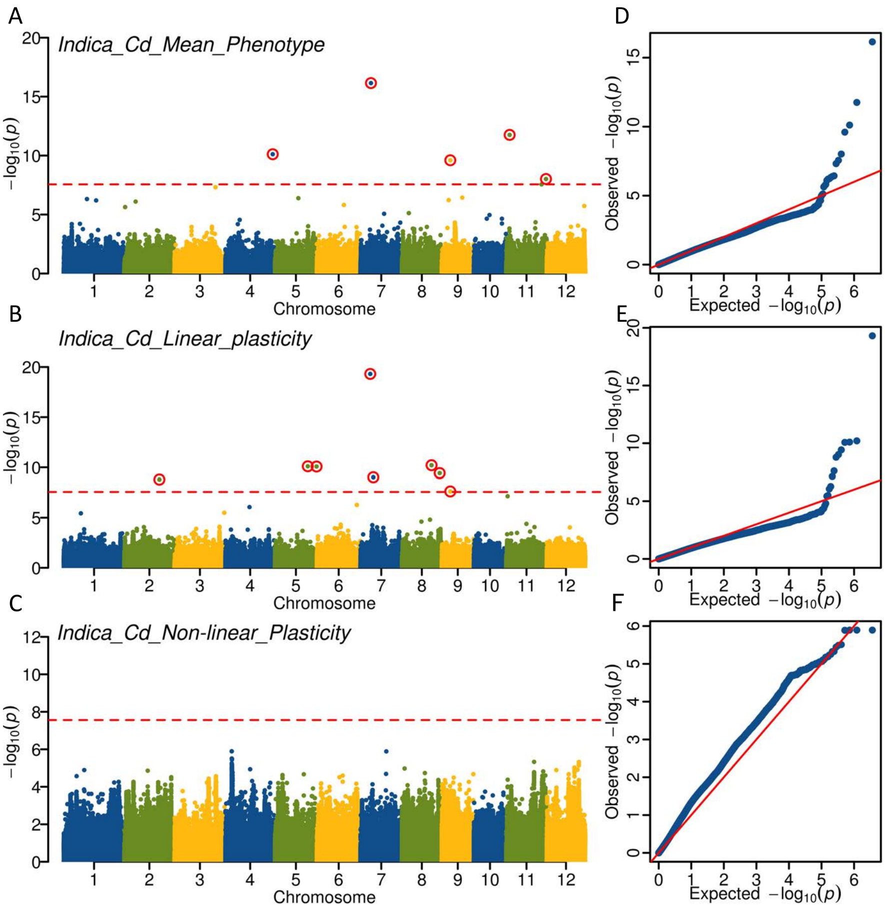

Fig. S 12 GWAS results of three phenotypic measures of Cd in the *Indica* subspecies. (A-C) Manhattan plots of (A) the mean phenotype, (B) the linear plasticity, and (C) the non-linear plasticity, the horizontal dashed line indicates the threshold at 2.7e-8. The significantly associated loci (SALs) were labeled with red circles. (D-F) Quantile-quantile plots of the mean phenotype (D), the linear plasticity (E), and the non-linear plasticity (F).

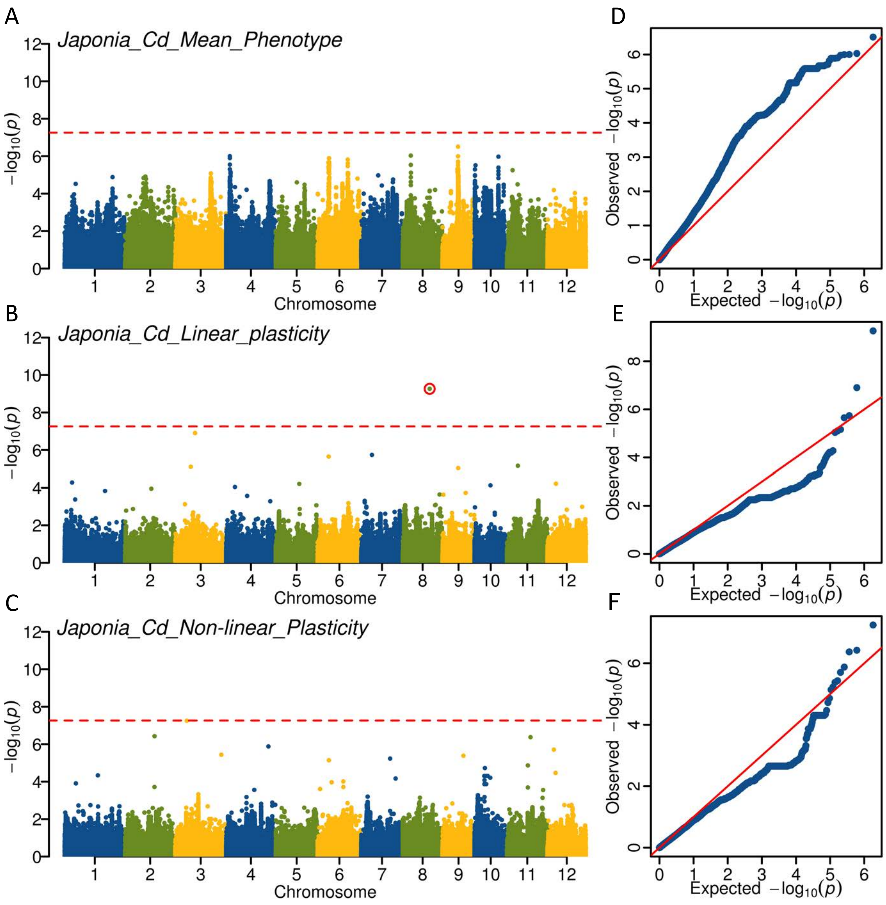

Fig. S 13 GWAS results of three phenotypic measures of Cd in the *Japonica* subspecies. (A-C) Manhattan plots of (A) the mean phenotype, (B) the linear plasticity, and (C) the non-linear plasticity, the horizontal dashed line indicates the threshold at 5.5e-8. The significantly associated loci (SALs) were labeled with red circles. (D-F) Quantile-quantile plots of the mean phenotype (D), the linear plasticity (E), and the non-linear plasticity (F).

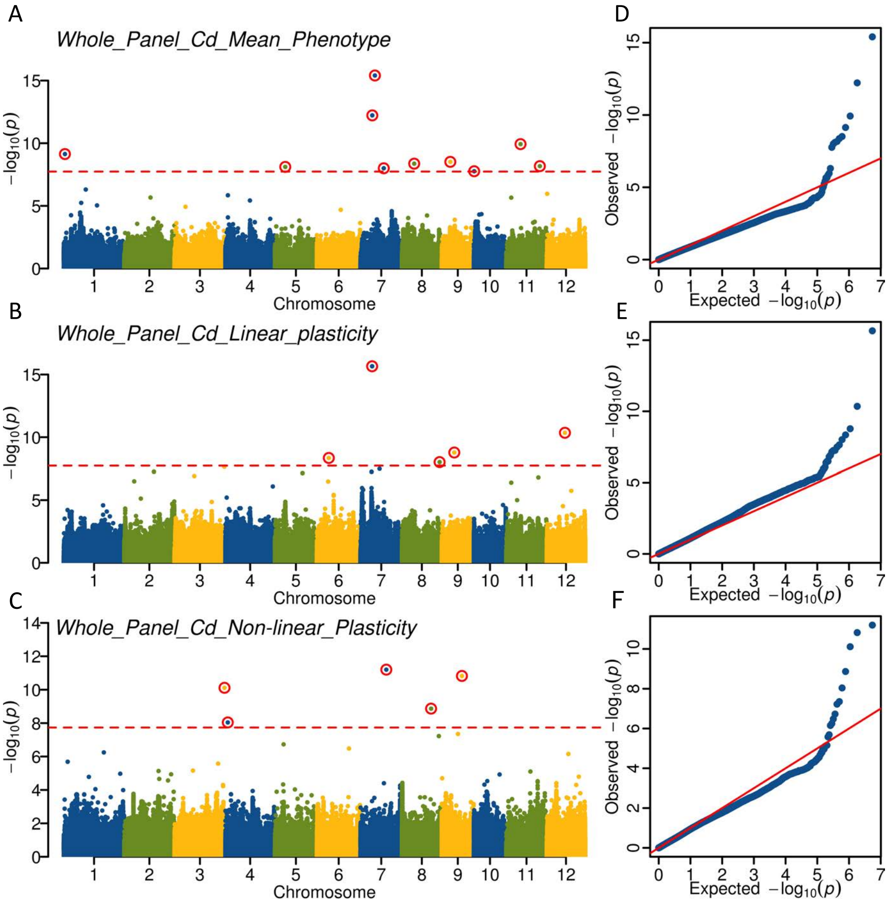

Fig. S 14 GWAS results of three phenotypic measures of Cd in the whole panel. (A-C) Manhattan plots of (A) the mean phenotype, (B) the linear plasticity, and (C) the non-linear plasticity, the horizontal dashed line indicates the threshold at 1.8e-8. The significantly associated loci (SALs) were labeled with red circles. (D-F) Quantile-quantile plots of the mean phenotype (D), the linear plasticity (E), and the non-linear plasticity (F).

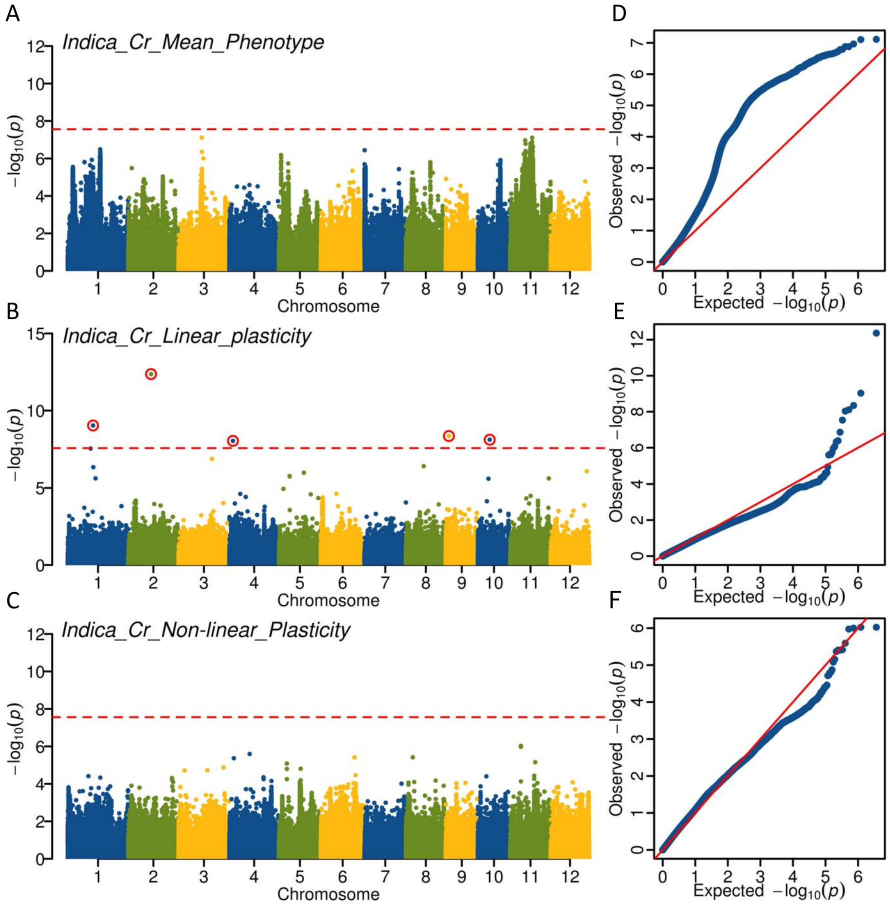

Fig. S 15 GWAS results of three phenotypic measures of Cr in the *Indica* subspecies. (A-C) Manhattan plots of (A) the mean phenotype, (B) the linear plasticity, and (C) the non-linear plasticity, the horizontal dashed line indicates the threshold at 2.7e-8. The significantly associated loci (SALs) were labeled with red circles. (D-F) Quantile-quantile plots of the mean phenotype (D), the linear plasticity (E), and the non-linear plasticity (F).

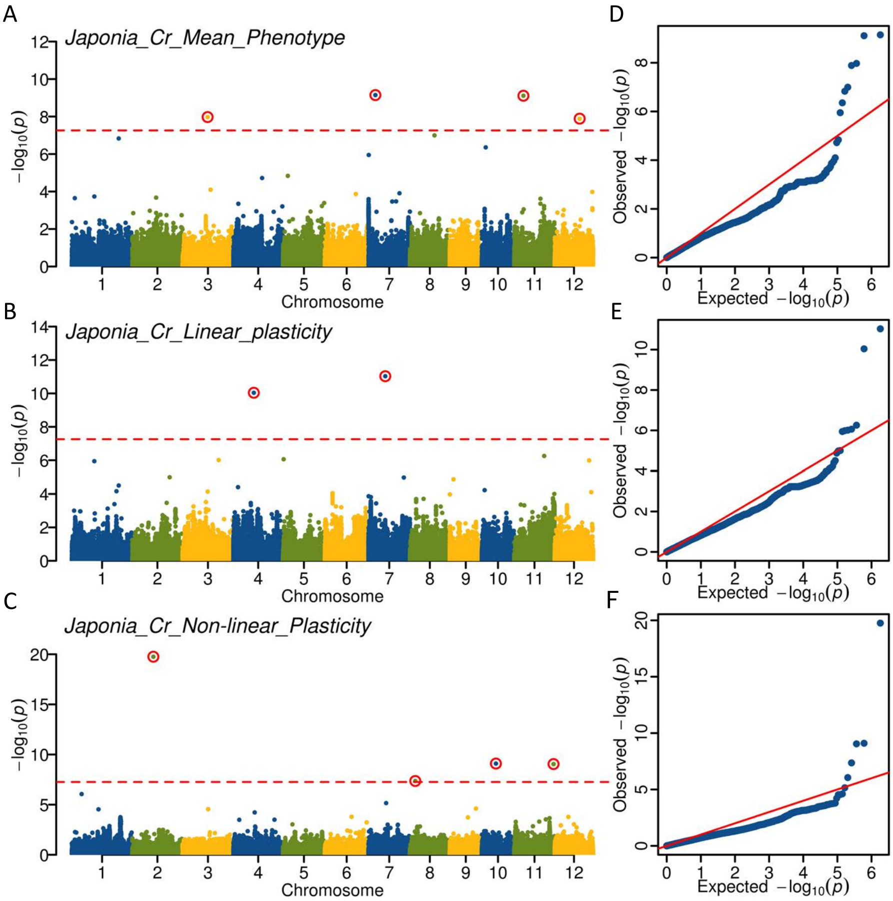

Fig. S 16 GWAS results of three phenotypic measures of Cr in the *Japonica* subspecies. (A-C) Manhattan plots of (A) the mean phenotype, (B) the linear plasticity, and (C) the non-linear plasticity, the horizontal dashed line indicates the threshold at 5.5e-8. The significantly associated loci (SALs) were labeled with red circles. (D-F) Quantile-quantile plots of the mean phenotype (D), the linear plasticity (E), and the non-linear plasticity (F).

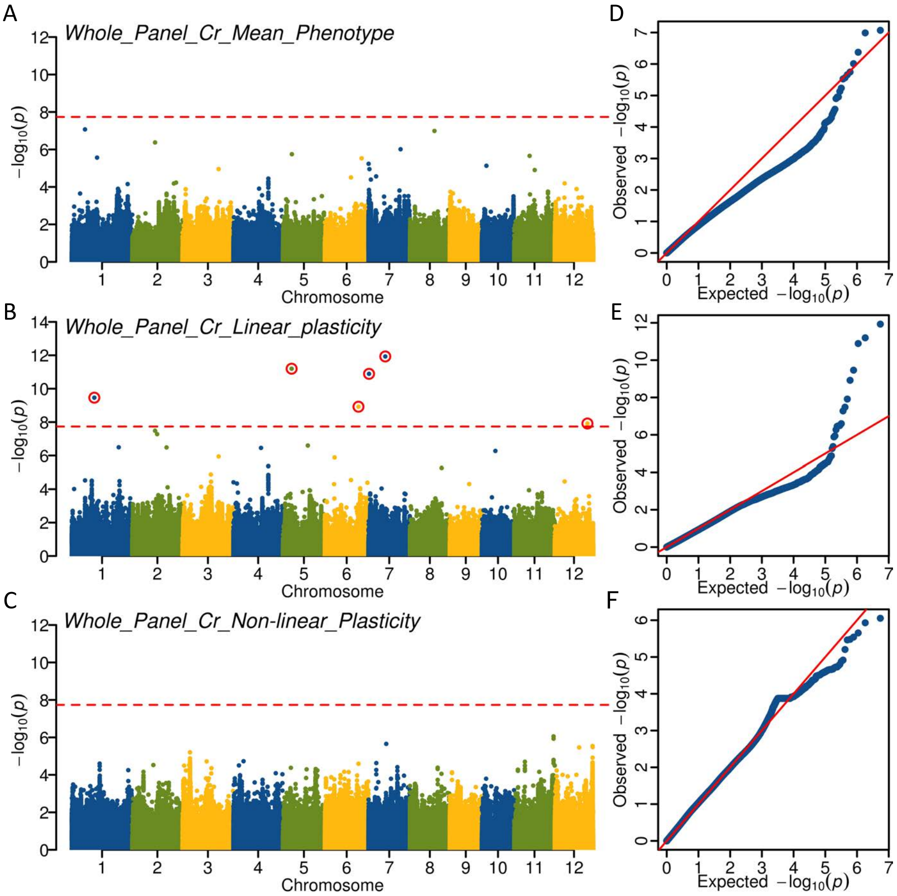

Fig. S 17 GWAS results of three phenotypic measures of Cr in the whole panel. (A-C) Manhattan plots of (A) the mean phenotype, (B) the linear plasticity, and (C) the non-linear plasticity, the horizontal dashed line indicates the threshold at 1.8e-8. The significantly associated loci (SALs) were labeled with red circles. (D-F) Quantile-quantile plots of the mean phenotype (D), the linear plasticity (E), and the non-linear plasticity (F).

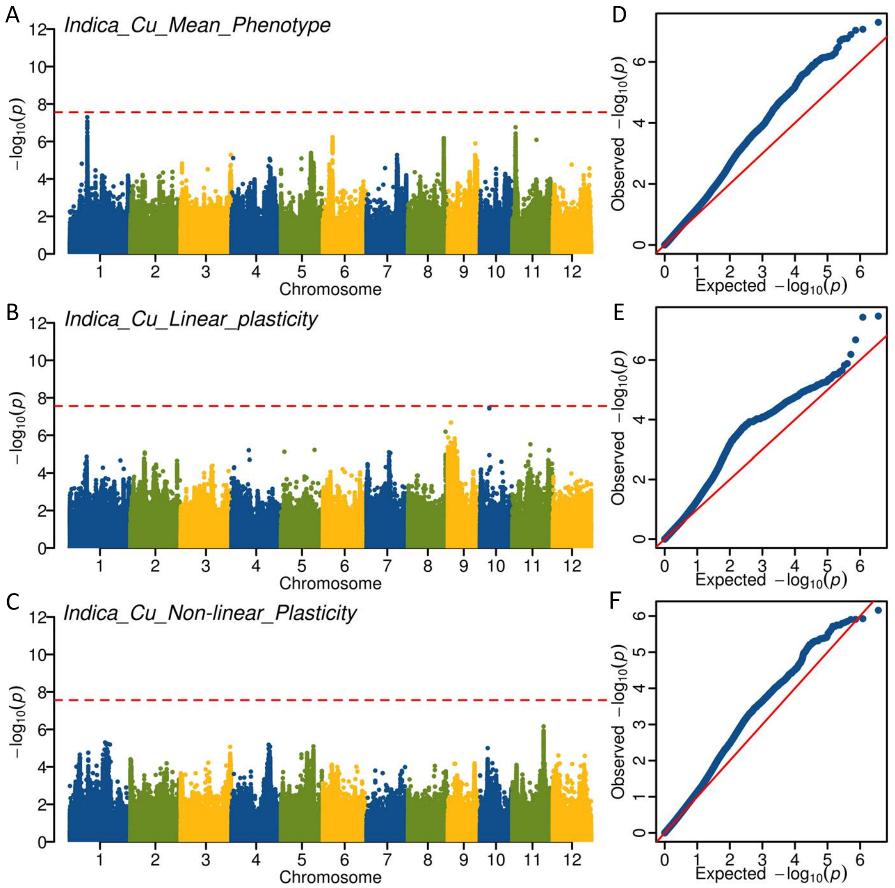

Fig. S 18 GWAS results of three phenotypic measures of Cu in the *Indica* subspecies. (A-C) Manhattan plots of (A) the mean phenotype, (B) the linear plasticity, and (C) the non-linear plasticity, the horizontal dashed line indicates the threshold at 2.7e-8. The significantly associated loci (SALs) were labeled with red circles. (D-F) Quantile-quantile plots of the mean phenotype (D), the linear plasticity (E), and the non-linear plasticity (F).

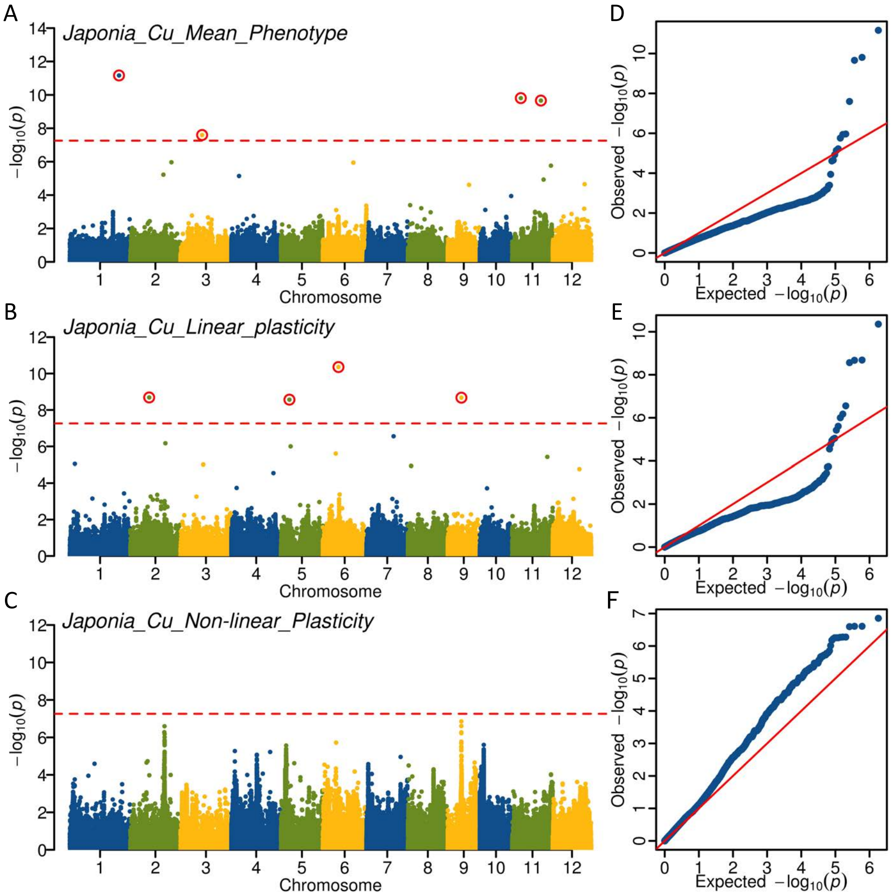

Fig. S 19 GWAS results of three phenotypic measures of Cu in the *Japonica* subspecies. (A-C) Manhattan plots of (A) the mean phenotype, (B) the linear plasticity, and (C) the non-linear plasticity, the horizontal dashed line indicates the threshold at 5.5e-8. The significantly associated loci (SALs) were labeled with red circles. (D-F) Quantile-quantile plots of the mean phenotype (D), the linear plasticity (E), and the non-linear plasticity (F).

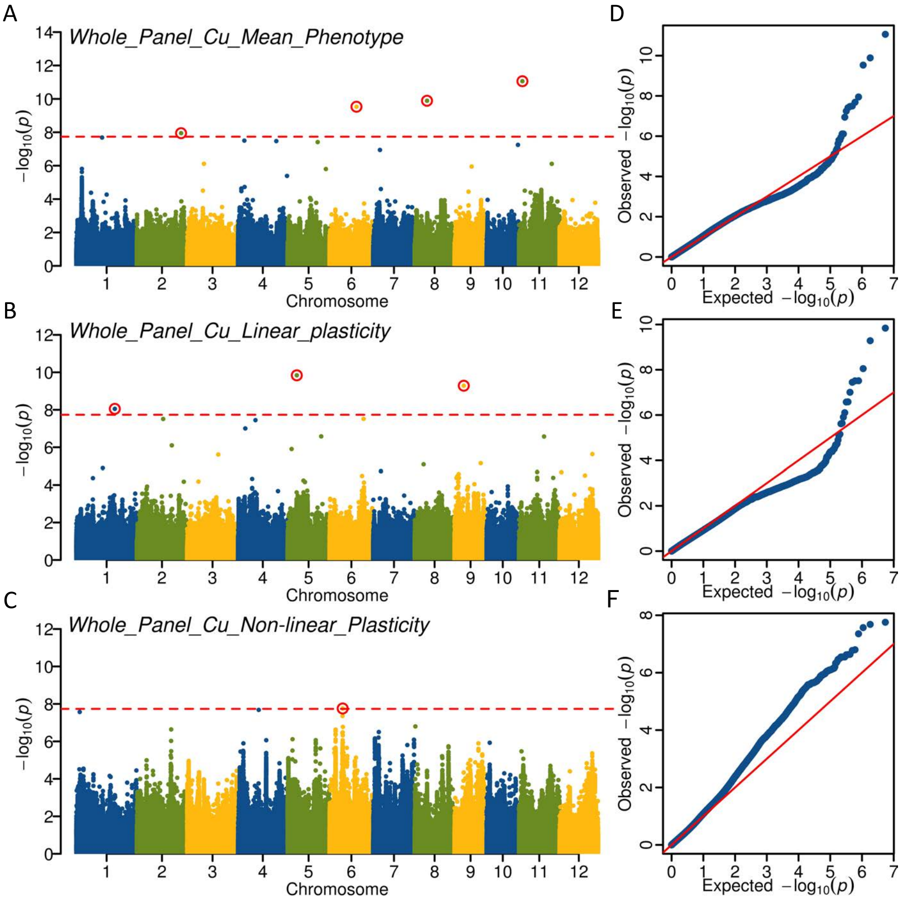

Fig. S 20 GWAS results of three phenotypic measures of Cu in the whole panel. (A-C) Manhattan plots of (A) the mean phenotype, (B) the linear plasticity, and (C) the non-linear plasticity, the horizontal dashed line indicates the threshold at 1.8e-8. The significantly associated loci (SALs) were labeled with red circles. (D-F) Quantile-quantile plots of the mean phenotype (D), the linear plasticity (E), and the non-linear plasticity (F).

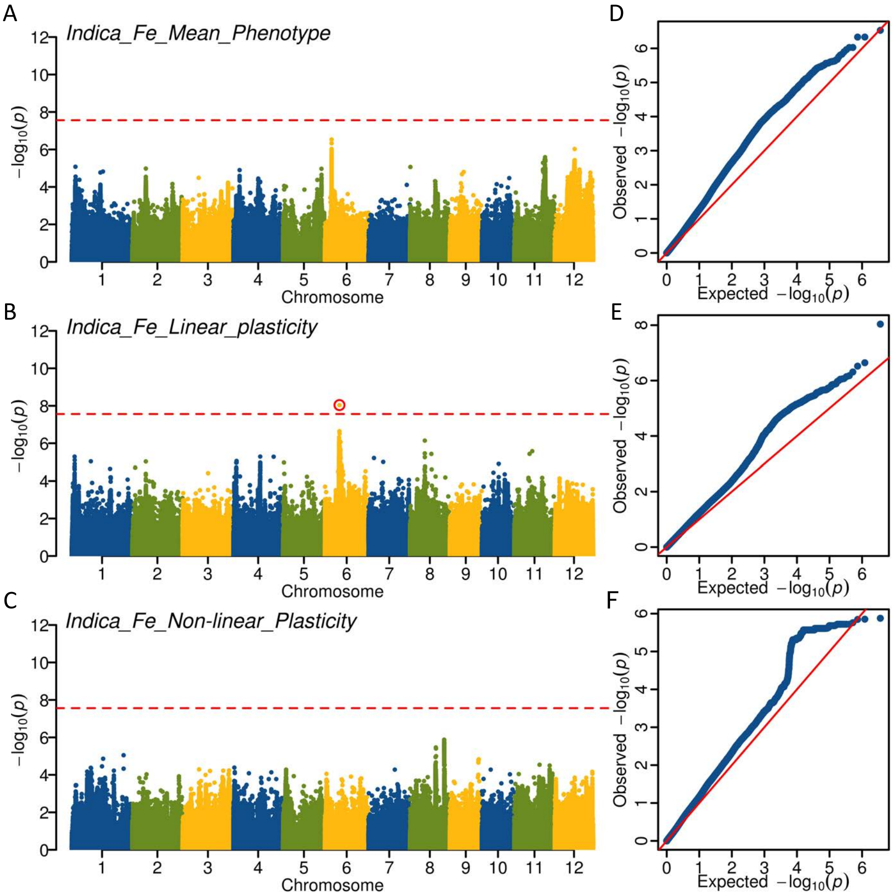

Fig. S 21 GWAS results of three phenotypic measures of Fe in the *Indica* subspecies. (A-C) Manhattan plots of (A) the mean phenotype, (B) the linear plasticity, and (C) the non-linear plasticity, the horizontal dashed line indicates the threshold at 2.7e-8. The significantly associated loci (SALs) were labeled with red circles. (D-F) Quantile-quantile plots of the mean phenotype (D), the linear plasticity (E), and the non-linear plasticity (F).

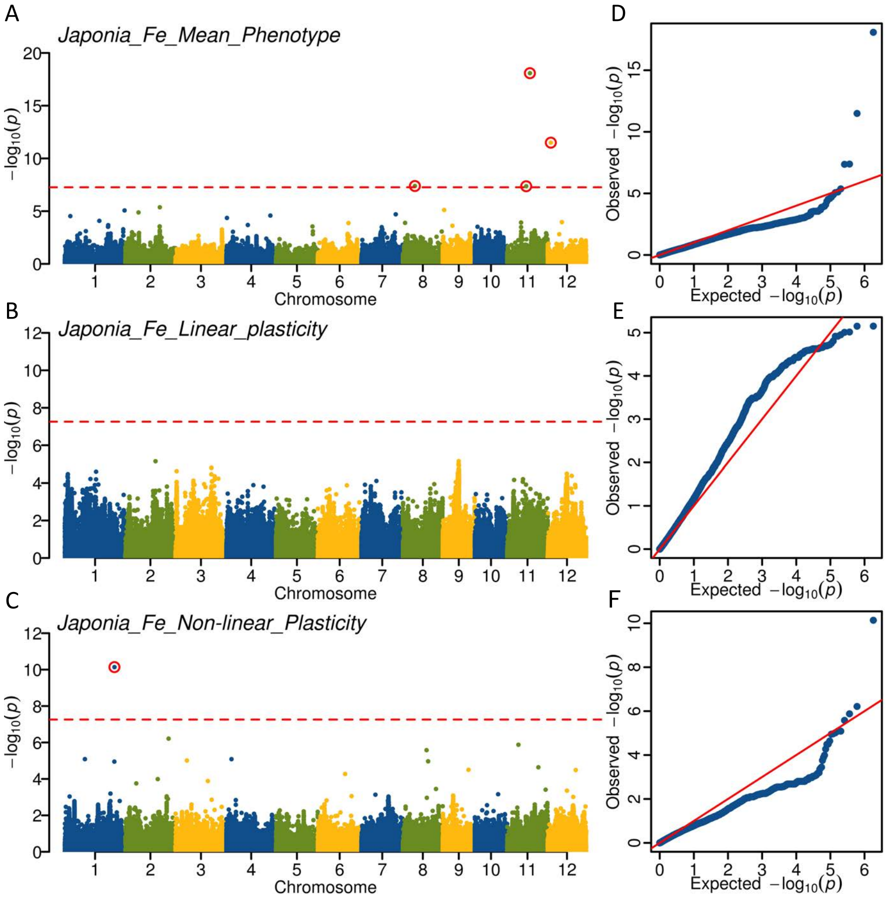

Fig. S 22 GWAS results of three phenotypic measures of Fe in the *Japonica* subspecies. (A-C) Manhattan plots of (A) the mean phenotype, (B) the linear plasticity, and (C) the non-linear plasticity, the horizontal dashed line indicates the threshold at 5.5e-8. The significantly associated loci (SALs) were labeled with red circles. (D-F) Quantile-quantile plots of the mean phenotype (D), the linear plasticity (E), and the non-linear plasticity (F).

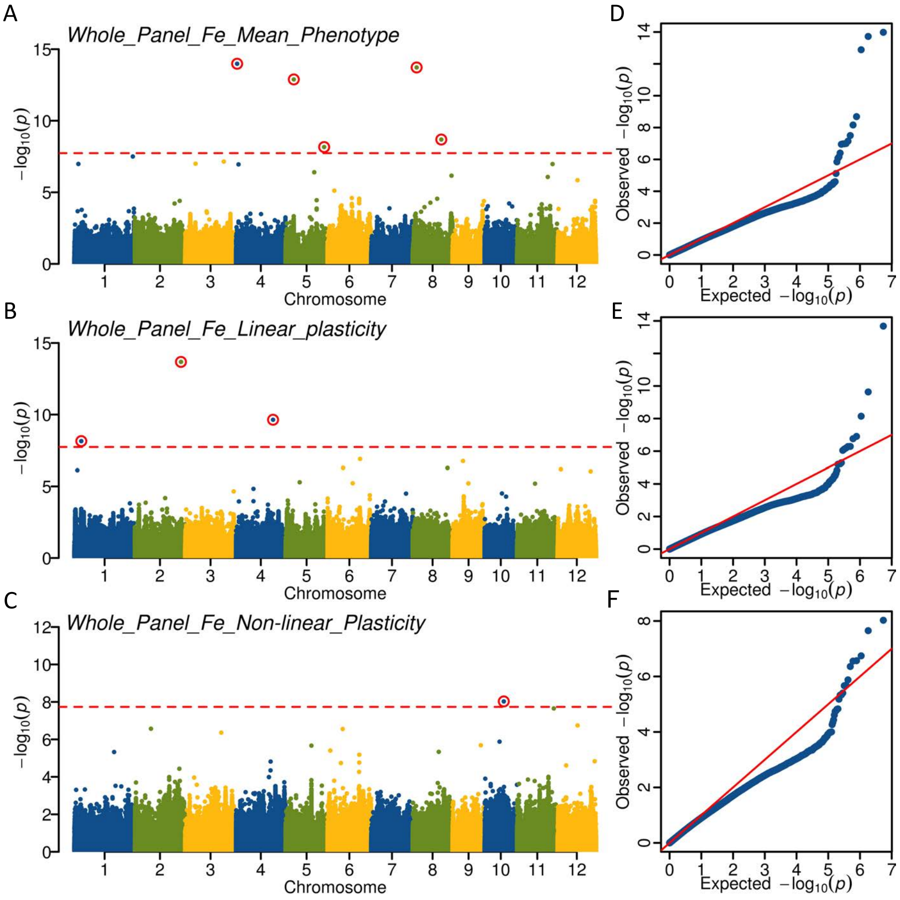

Fig. S 23 GWAS results of three phenotypic measures of Fe in the whole panel. (A-C) Manhattan plots of (A) the mean phenotype, (B) the linear plasticity, and (C) the non-linear plasticity, the horizontal dashed line indicates the threshold at 1.8e-8. The significantly associated loci (SALs) were labeled with red circles. (D-F) Quantile-quantile plots of the mean phenotype (D), the linear plasticity (E), and the non-linear plasticity (F).

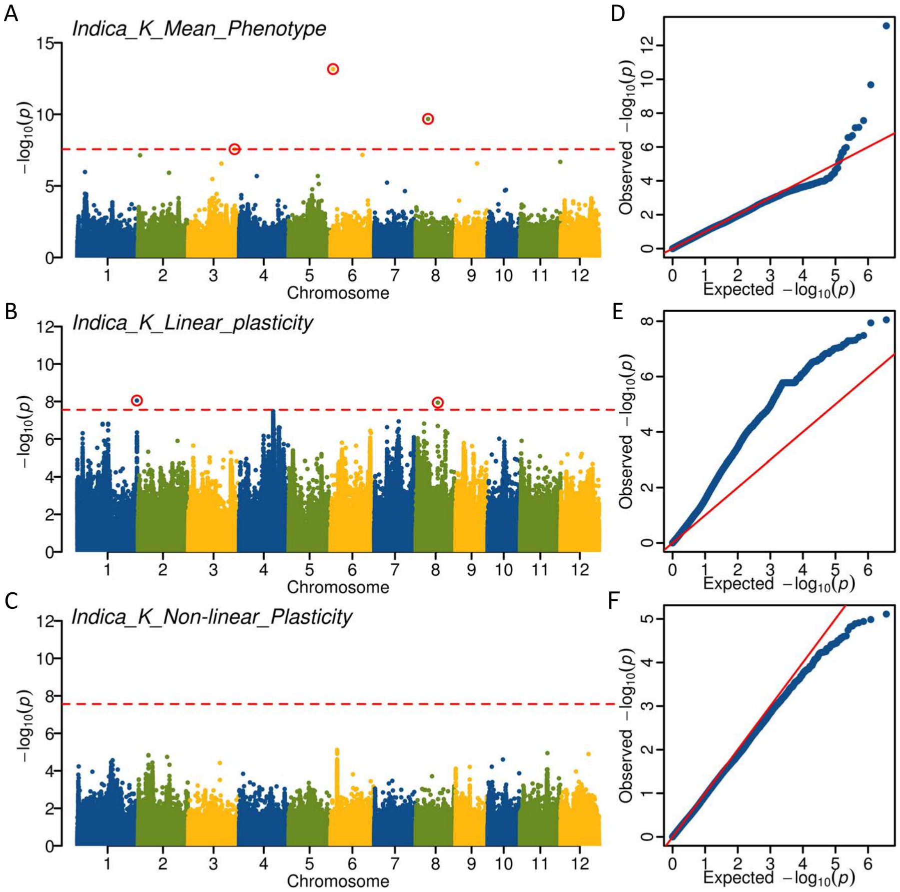

Fig. S 24 GWAS results of three phenotypic measures of K in the *Indica* subspecies. (A-C) Manhattan plots of (A) the mean phenotype, (B) the linear plasticity, and (C) the non-linear plasticity, the horizontal dashed line indicates the threshold at 2.7e-8. The significantly associated loci (SALs) were labeled with red circles. (D-F) Quantile-quantile plots of the mean phenotype (D), the linear plasticity (E), and the non-linear plasticity (F).

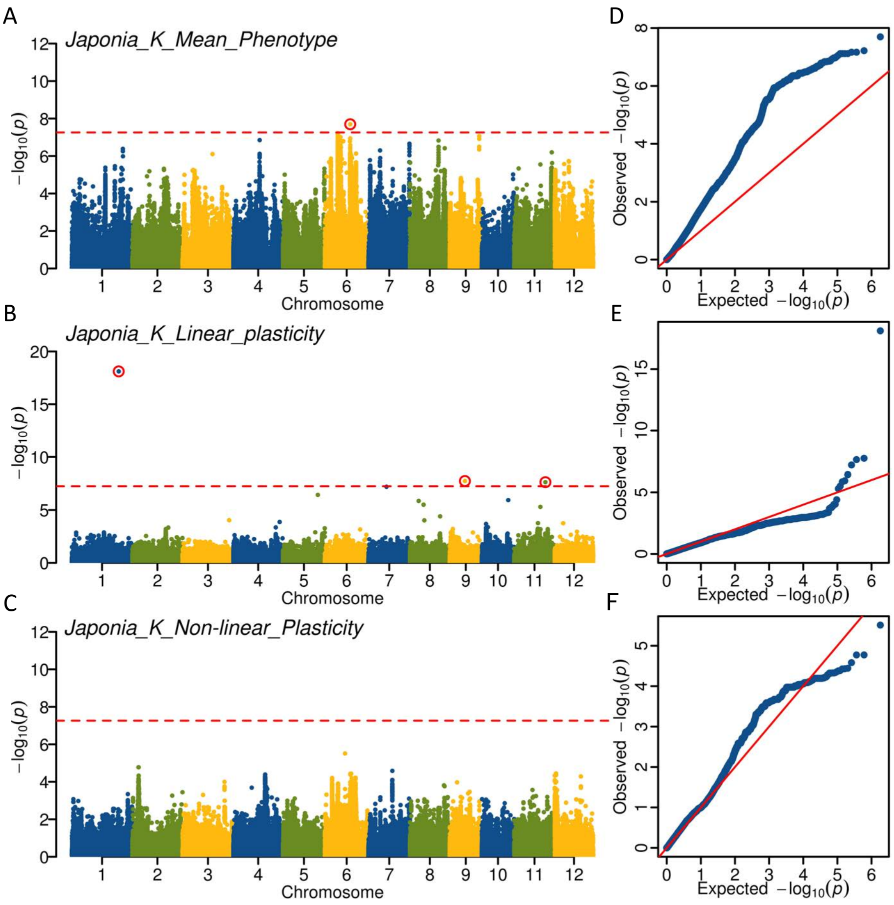

Fig. S 25 GWAS results of three phenotypic measures of K in the *Japonica* subspecies. (A-C) Manhattan plots of (A) the mean phenotype, (B) the linear plasticity, and (C) the non-linear plasticity, the horizontal dashed line indicates the threshold at 5.5e-8. The significantly associated loci (SALs) were labeled with red circles. (D-F) Quantile-quantile plots of the mean phenotype (D), the linear plasticity (E), and the non-linear plasticity (F).

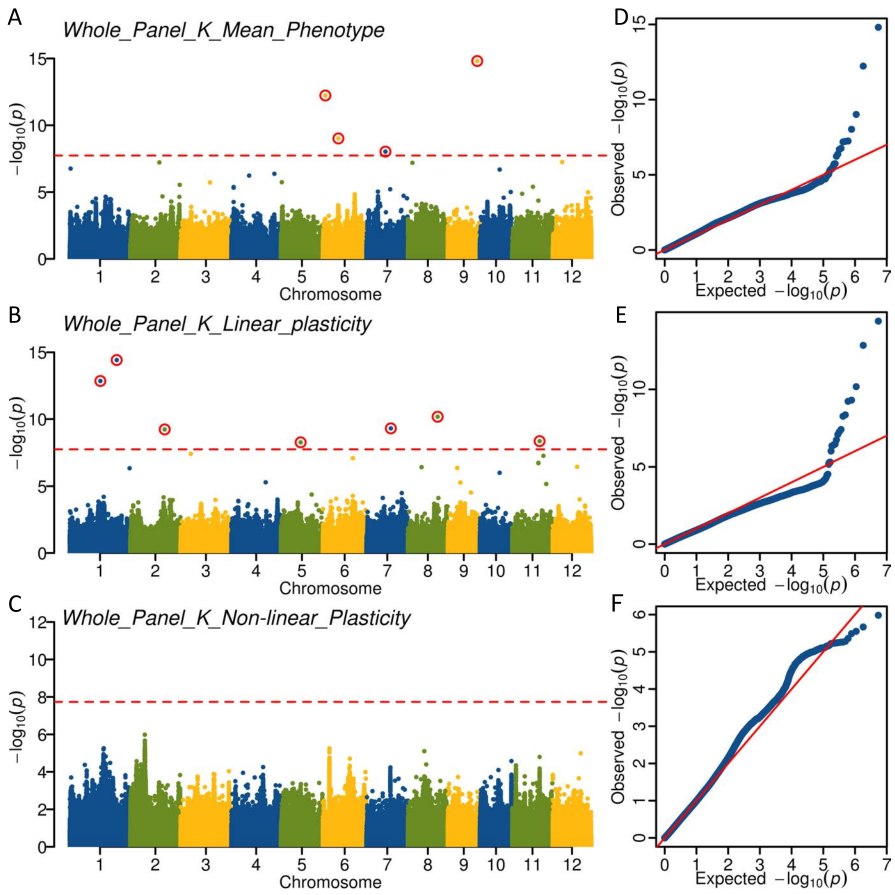

Fig. S 26 GWAS results of three phenotypic measures of K in the whole panel. (A-C) Manhattan plots of (A) the mean phenotype, (B) the linear plasticity, and (C) the non-linear plasticity, the horizontal dashed line indicates the threshold at 1.8e-8. The significantly associated loci (SALs) were labeled with red circles. (D-F) Quantile-quantile plots of the mean phenotype (D), the linear plasticity (E), and the non-linear plasticity (F).

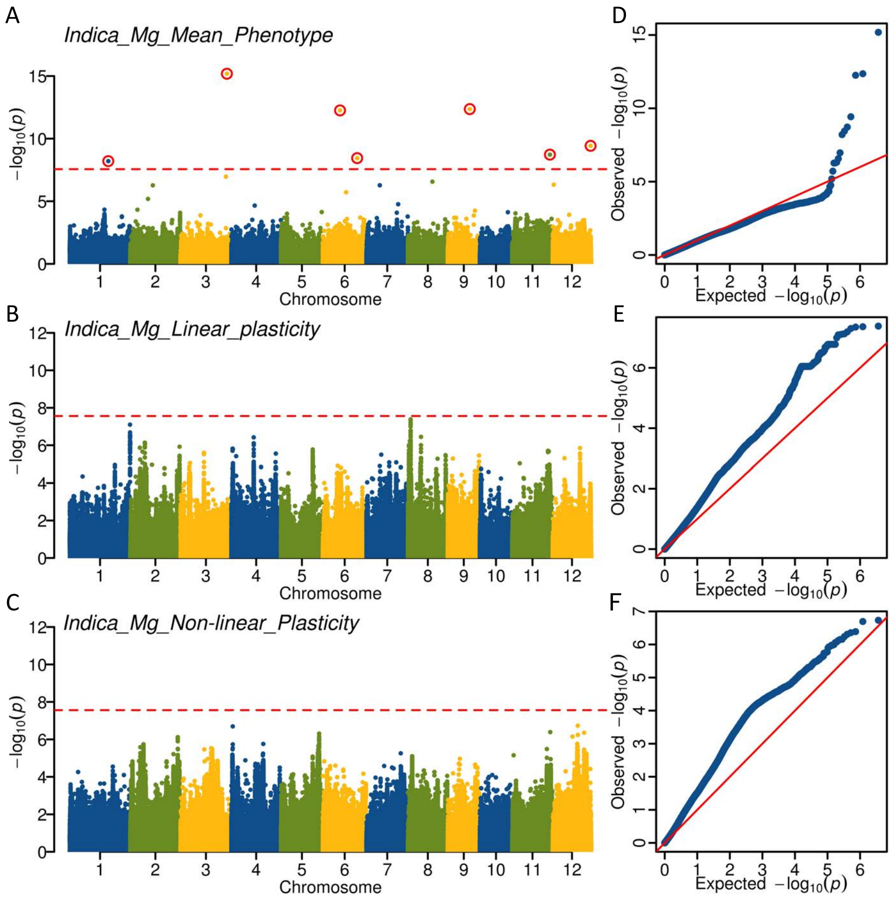

Fig. S 27 GWAS results of three phenotypic measures of Mg in the *Indica* subspecies. (A-C) Manhattan plots of (A) the mean phenotype, (B) the linear plasticity, and (C) the non-linear plasticity, the horizontal dashed line indicates the threshold at 2.7e-8. The significantly associated loci (SALs) were labeled with red circles. (D-F) Quantile-quantile plots of the mean phenotype (D), the linear plasticity (E), and the non-linear plasticity (F).

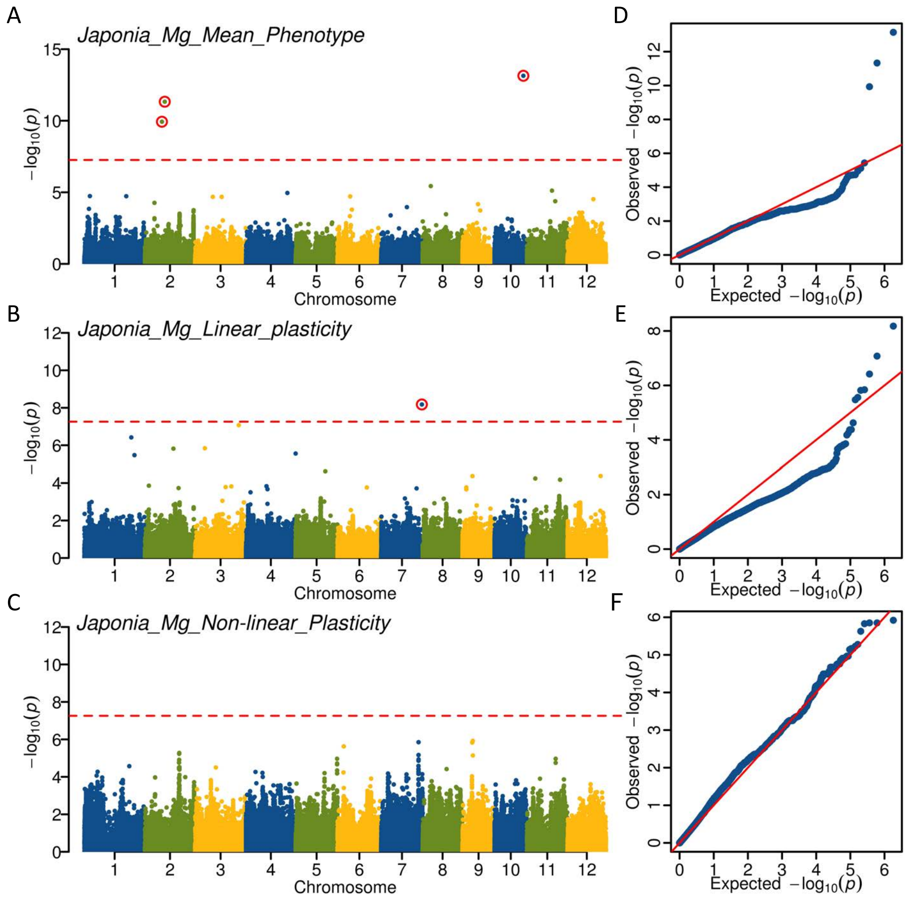

Fig. S 28 GWAS results of three phenotypic measures of Mg in the *Japonica* subspecies. (A-C) Manhattan plots of (A) the mean phenotype, (B) the linear plasticity, and (C) the non-linear plasticity, the horizontal dashed line indicates the threshold at 5.5e-8. The significantly associated loci (SALs) were labeled with red circles. (D-F) Quantile-quantile plots of the mean phenotype (D), the linear plasticity (E), and the non-linear plasticity (F).

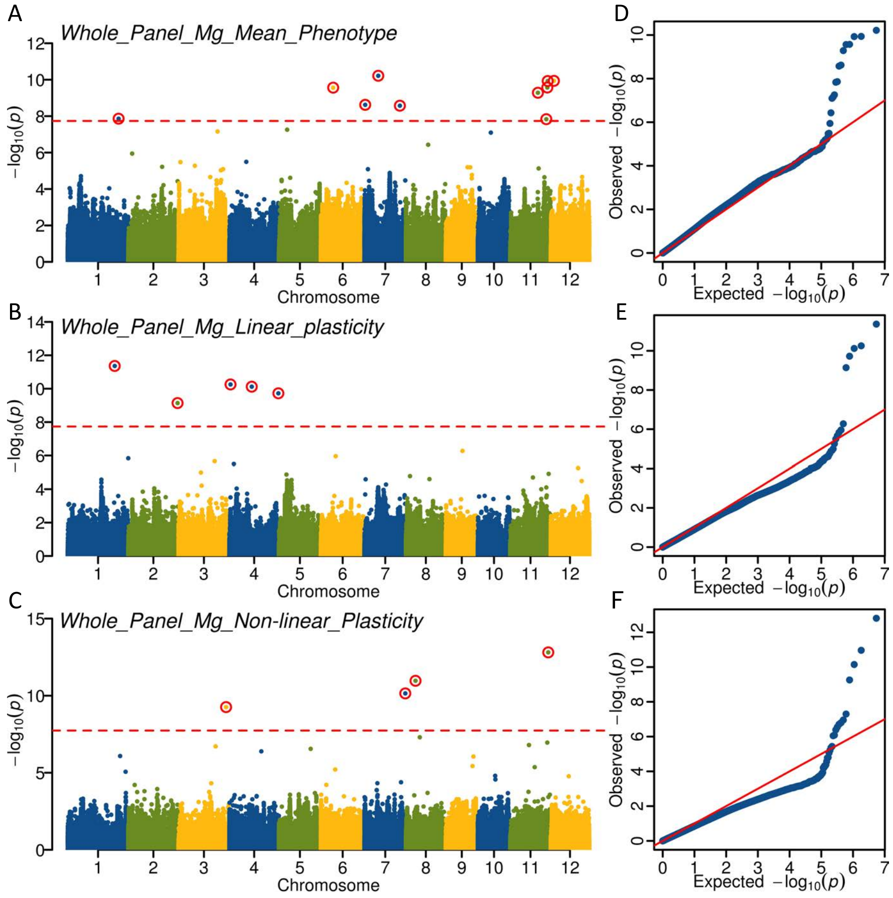

Fig. S 29 GWAS results of three phenotypic measures of Mg in the whole panel. (A-C) Manhattan plots of (A) the mean phenotype, (B) the linear plasticity, and (C) the non-linear plasticity, the horizontal dashed line indicates the threshold at 1.8e-8. The significantly associated loci (SALs) were labeled with red circles. (D-F) Quantile-quantile plots of the mean phenotype (D), the linear plasticity (E), and the non-linear plasticity (F).

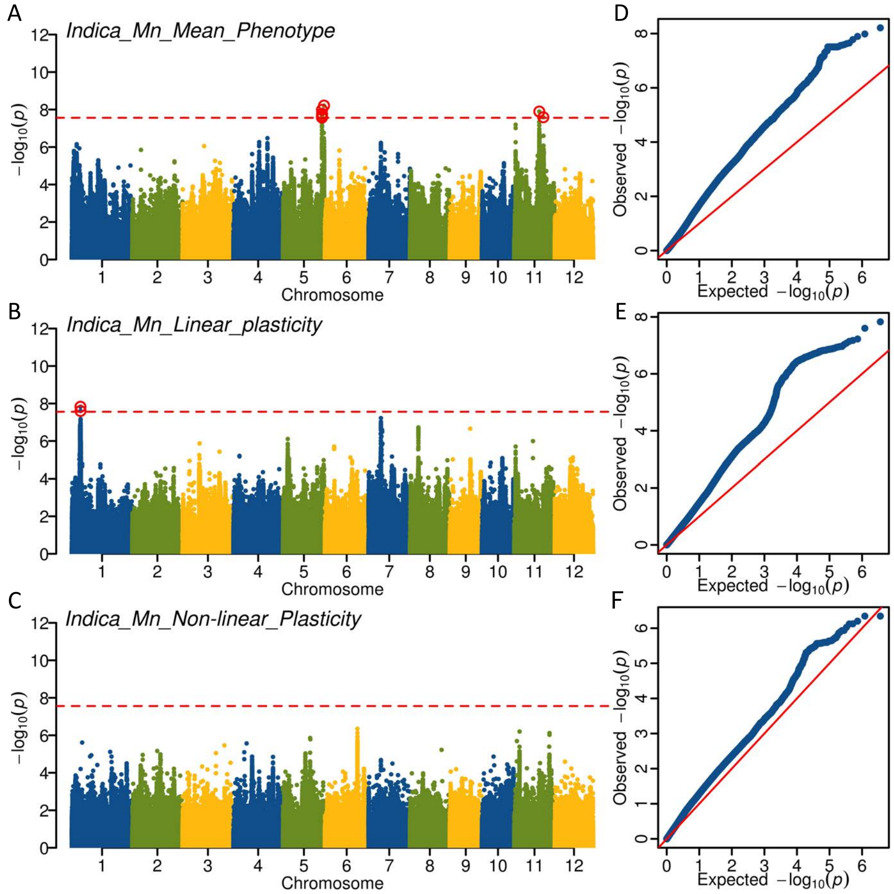

Fig. S 30 GWAS results of three phenotypic measures of Mn in the *Indica* subspecies. (A-C) Manhattan plots of (A) the mean phenotype, (B) the linear plasticity, and (C) the non-linear plasticity, the horizontal dashed line indicates the threshold at 2.7e-8. The significantly associated loci (SALs) were labeled with red circles. (D-F) Quantile-quantile plots of the mean phenotype (D), the linear plasticity (E), and the non-linear plasticity (F).

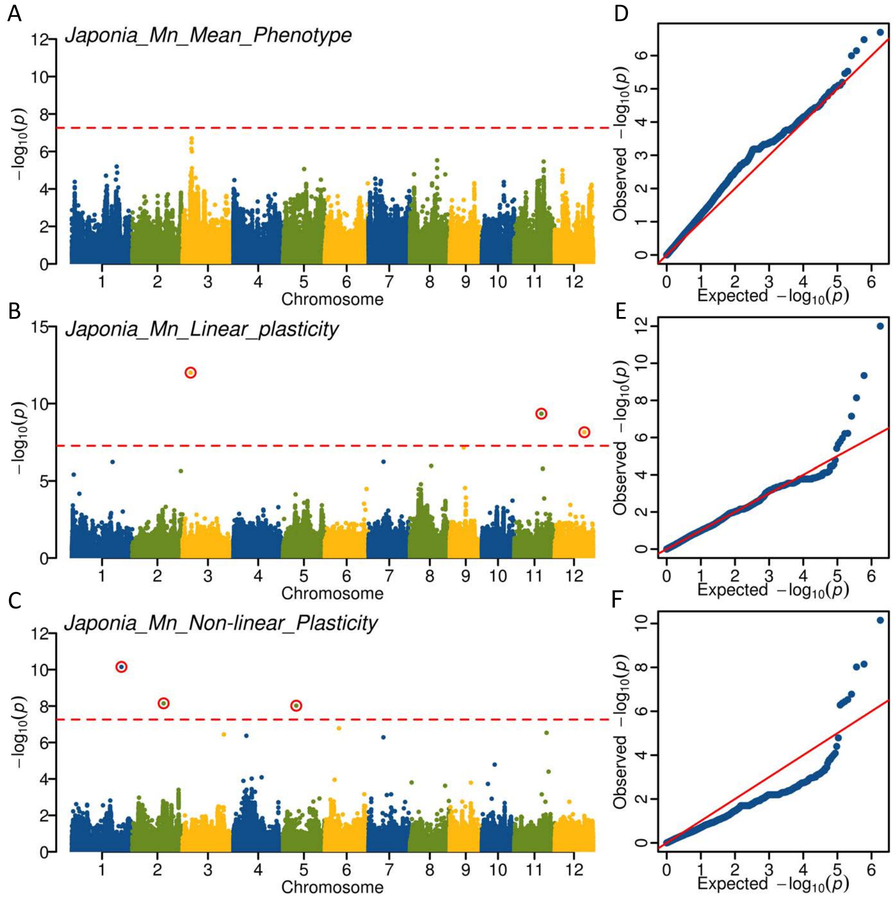

Fig. S 31 GWAS results of three phenotypic measures of Mn in the *Japonica* subspecies. (A-C) Manhattan plots of (A) the mean phenotype, (B) the linear plasticity, and (C) the non-linear plasticity, the horizontal dashed line indicates the threshold at 5.5e-8. The significantly associated loci (SALs) were labeled with red circles. (D-F) Quantile-quantile plots of the mean phenotype (D), the linear plasticity (E), and the non-linear plasticity (F).

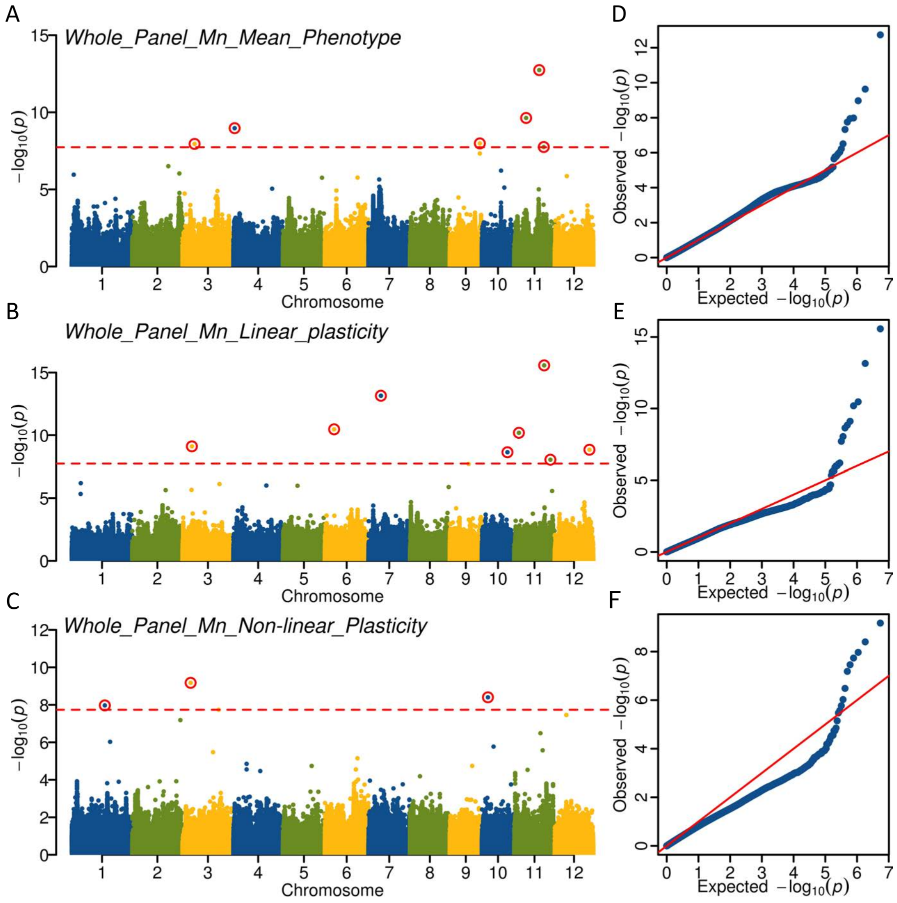

Fig. S 32 GWAS results of three phenotypic measures of Mn in the whole panel. (A-C) Manhattan plots of (A) the mean phenotype, (B) the linear plasticity, and (C) the non-linear plasticity, the horizontal dashed line indicates the threshold at 1.8e-8. The significantly associated loci (SALs) were labeled with red circles. (D-F) Quantile-quantile plots of the mean phenotype (D), the linear plasticity (E), and the non-linear plasticity (F).

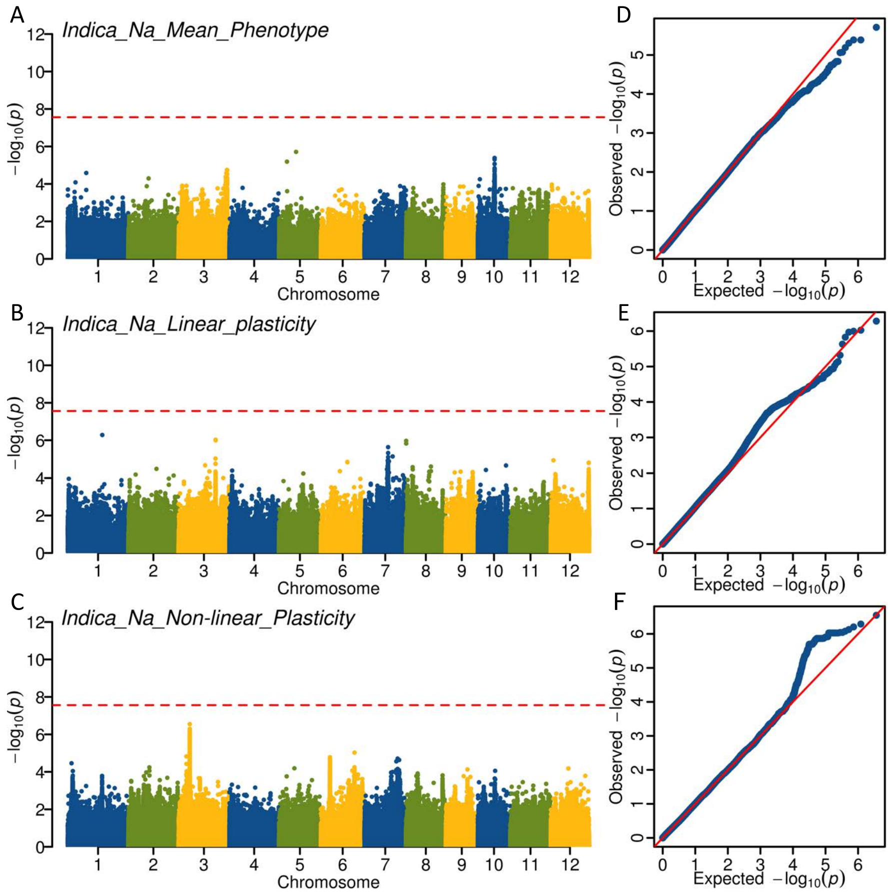

Fig. S 33 GWAS results of three phenotypic measures of Na in the *Indica* subspecies. (A-C) Manhattan plots of (A) the mean phenotype, (B) the linear plasticity, and (C) the non-linear plasticity, the horizontal dashed line indicates the threshold at 2.7e-8. The significantly associated loci (SALs) were labeled with red circles. (D-F) Quantile-quantile plots of the mean phenotype (D), the linear plasticity (E), and the non-linear plasticity (F).

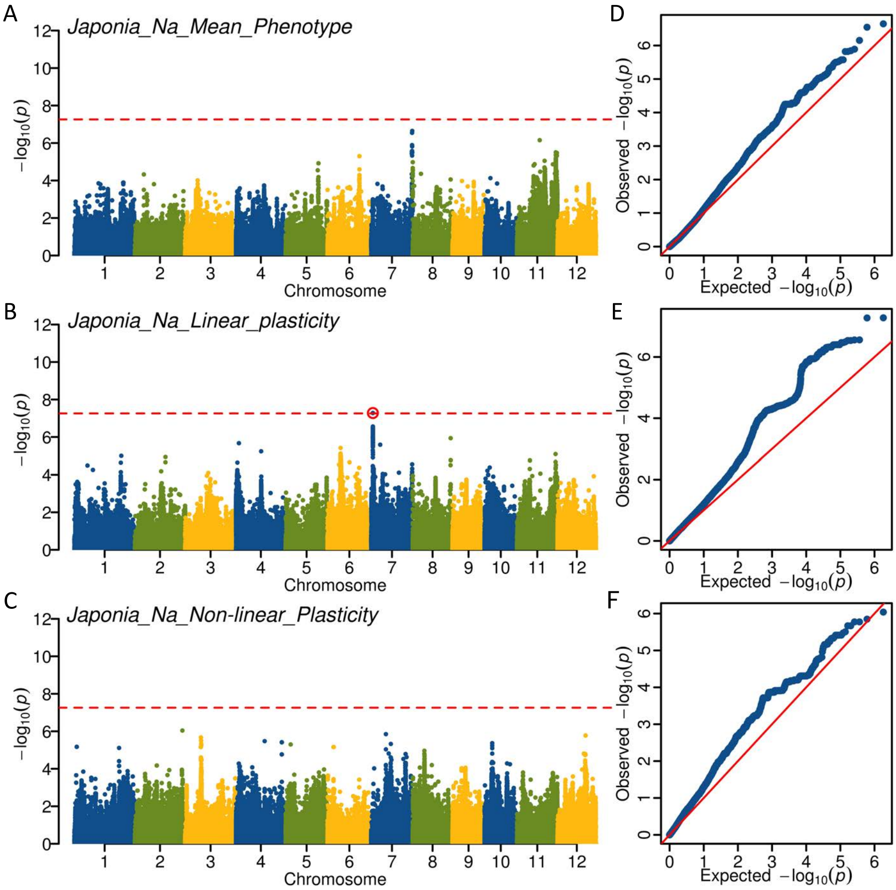

Fig. S 34 GWAS results of three phenotypic measures of Na in the *Japonica* subspecies. (A-C) Manhattan plots of (A) the mean phenotype, (B) the linear plasticity, and (C) the non-linear plasticity, the horizontal dashed line indicates the threshold at 5.5e-8. The significantly associated loci (SALs) were labeled with red circles. (D-F) Quantile-quantile plots of the mean phenotype (D), the linear plasticity (E), and the non-linear plasticity (F).

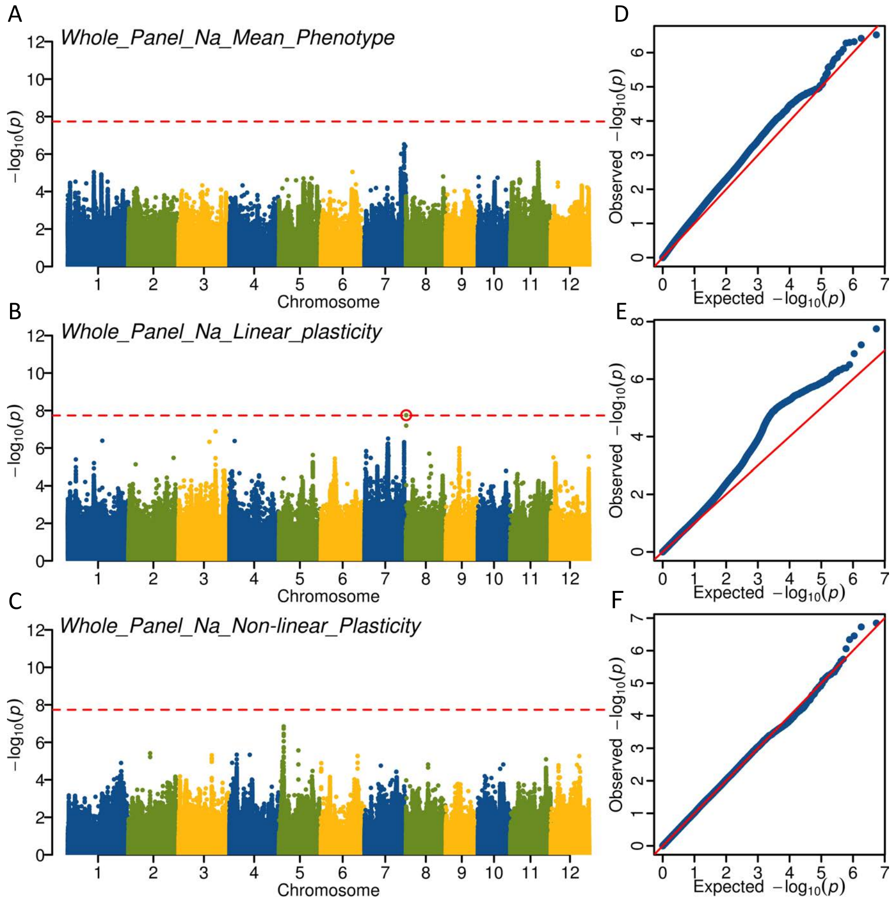

Fig. S 35 GWAS results of three phenotypic measures of Na in the whole panel. (A-C) Manhattan plots of (A) the mean phenotype, (B) the linear plasticity, and (C) the non-linear plasticity, the horizontal dashed line indicates the threshold at 1.8e-8. The significantly associated loci (SALs) were labeled with red circles. (D-F) Quantile-quantile plots of the mean phenotype (D), the linear plasticity (E), and the non-linear plasticity (F).

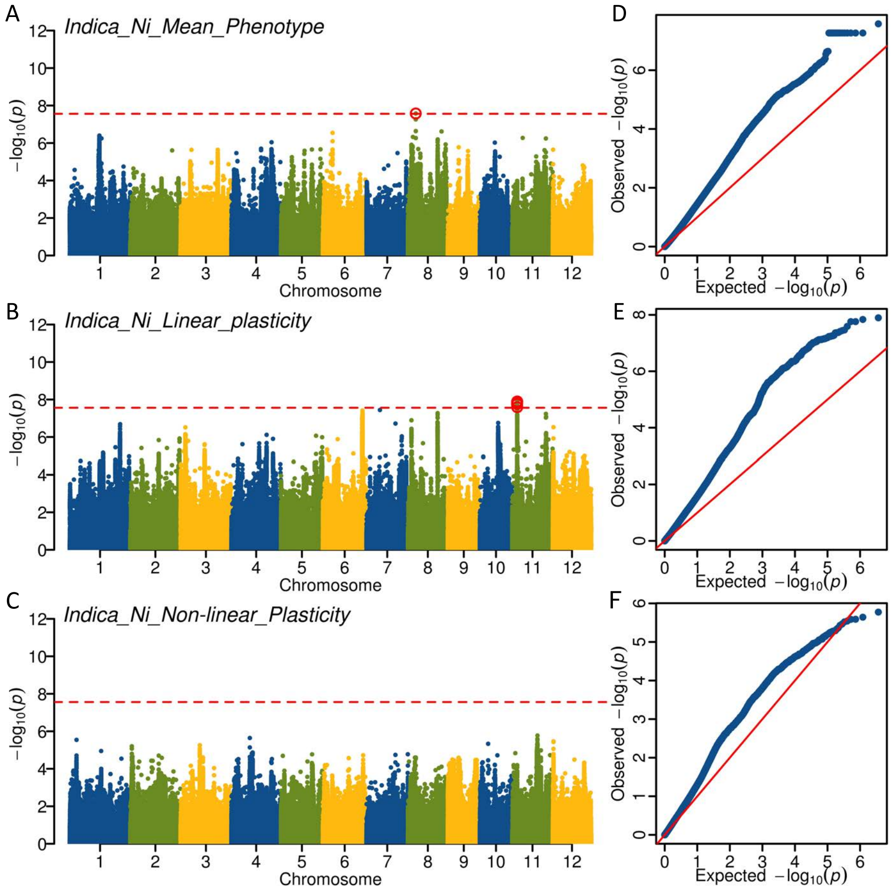

Fig. S 36 GWAS results of three phenotypic measures of Ni in the *Indica* subspecies. (A-C) Manhattan plots of (A) the mean phenotype, (B) the linear plasticity, and (C) the non-linear plasticity, the horizontal dashed line indicates the threshold at 2.7e-8. The significantly associated loci (SALs) were labeled with red circles. (D-F) Quantile-quantile plots of the mean phenotype (D), the linear plasticity (E), and the non-linear plasticity (F).

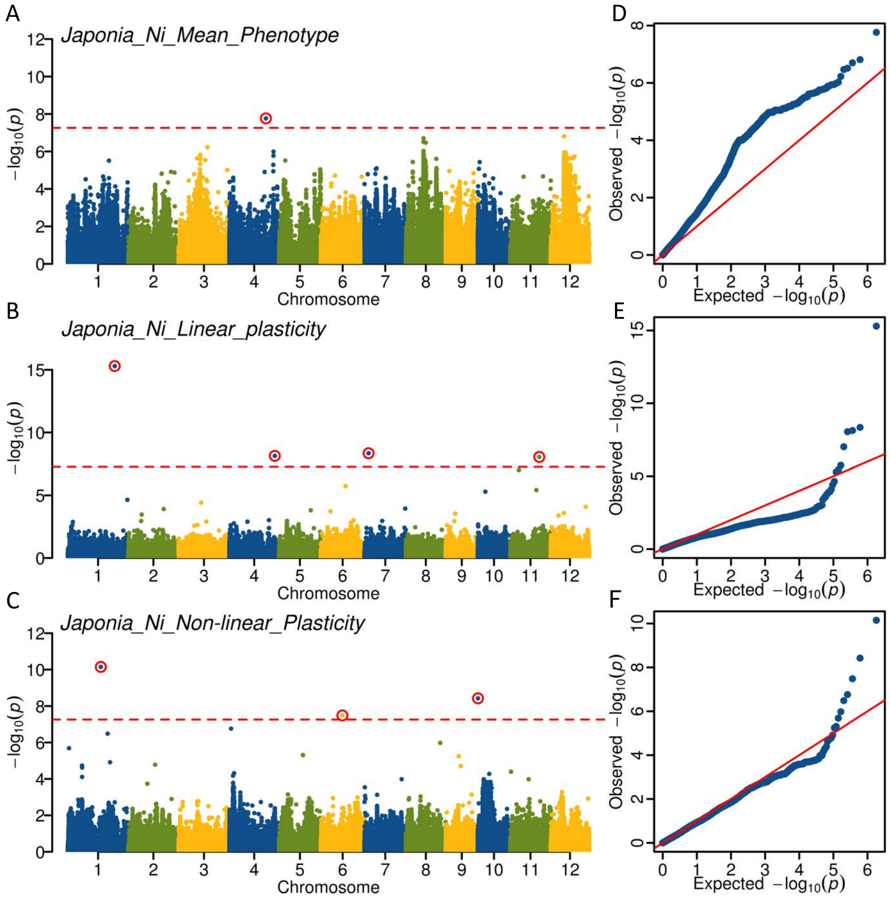

Fig. S 37 GWAS results of three phenotypic measures of Ni in the *Japonica* subspecies. (A-C) Manhattan plots of (A) the mean phenotype, (B) the linear plasticity, and (C) the non-linear plasticity, the horizontal dashed line indicates the threshold at 5.5e-8. The significantly associated loci (SALs) were labeled with red circles. (D-F) Quantile-quantile plots of the mean phenotype (D), the linear plasticity (E), and the non-linear plasticity (F).

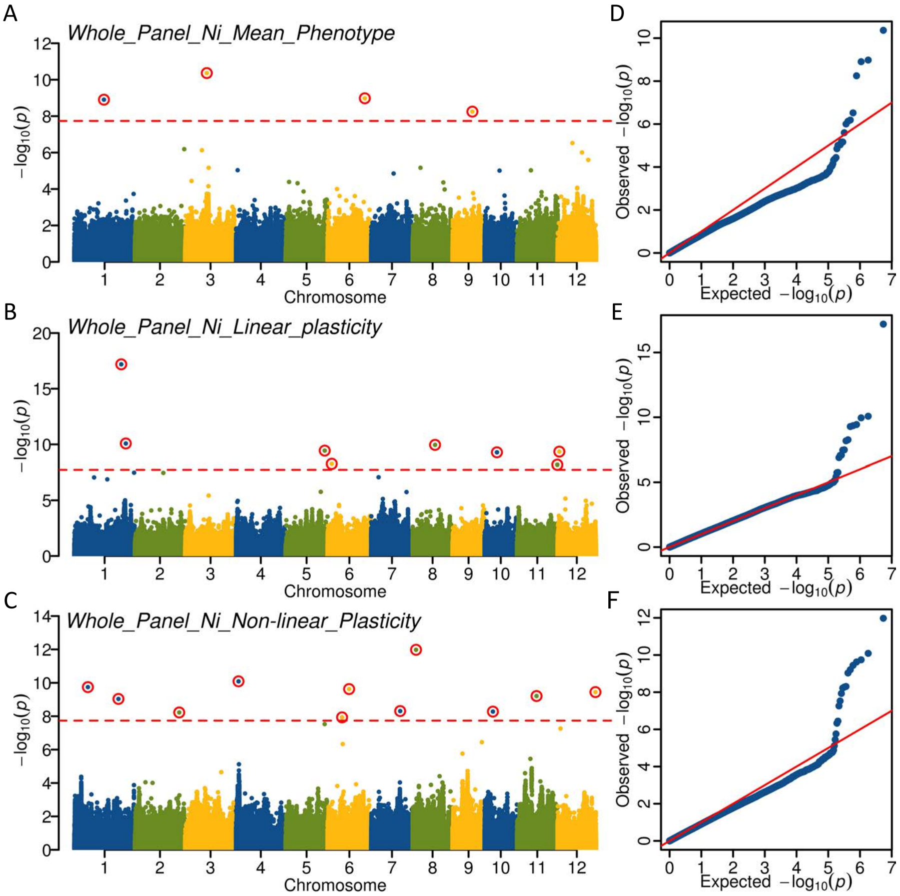

Fig. S 38 GWAS results of three phenotypic measures of Ni in the whole panel. (A-C) Manhattan plots of (A) the mean phenotype, (B) the linear plasticity, and (C) the non-linear plasticity, the horizontal dashed line indicates the threshold at 1.8e-8. The significantly associated loci (SALs) were labeled with red circles. (D-F) Quantile-quantile plots of the mean phenotype (D), the linear plasticity (E), and the non-linear plasticity (F).

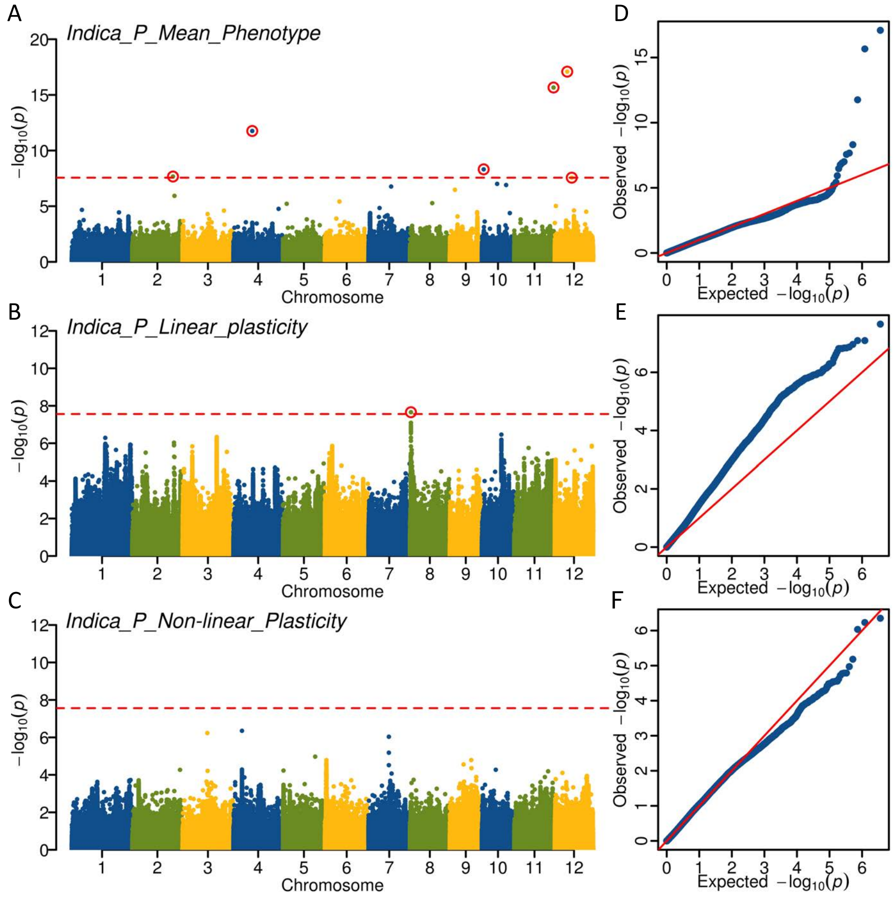

Fig. S 39 GWAS results of three phenotypic measures of P in the *Indica* subspecies. (A-C) Manhattan plots of (A) the mean phenotype, (B) the linear plasticity, and (C) the non-linear plasticity, the horizontal dashed line indicates the threshold at 2.7e-8. The significantly associated loci (SALs) were labeled with red circles. (D-F) Quantile-quantile plots of the mean phenotype (D), the linear plasticity (E), and the non-linear plasticity (F).

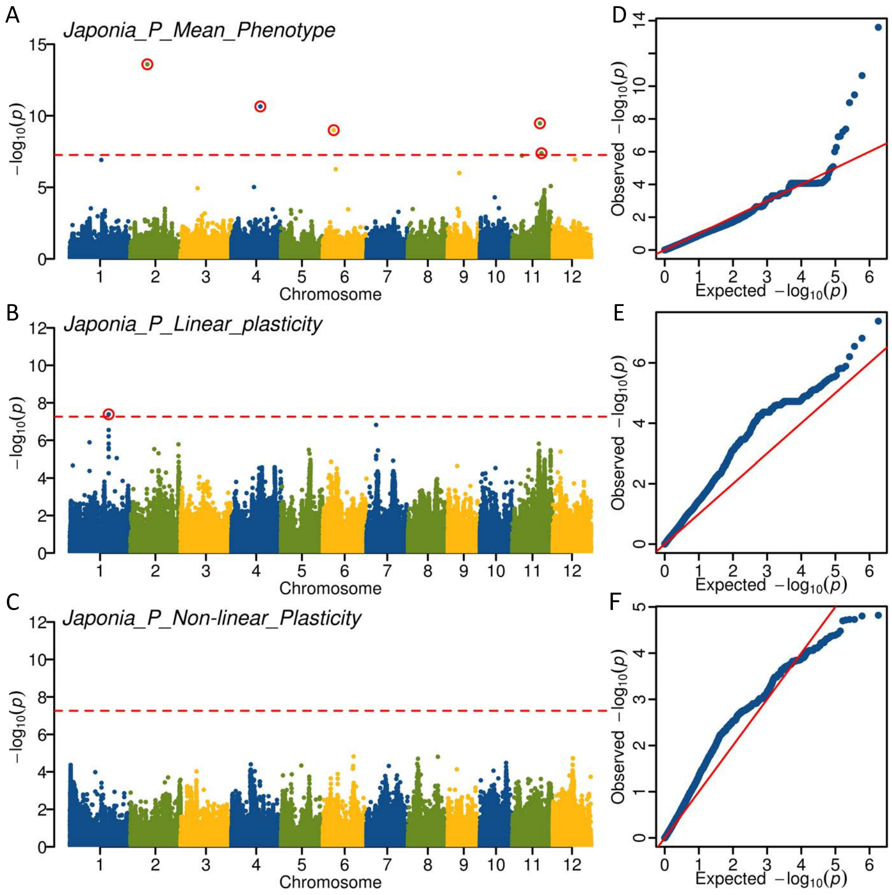

Fig. S 40 GWAS results of three phenotypic measures of P in the *Japonica* subspecies. (A-C) Manhattan plots of (A) the mean phenotype, (B) the linear plasticity, and (C) the non-linear plasticity, the horizontal dashed line indicates the threshold at 5.5e-8. The significantly associated loci (SALs) were labeled with red circles. (D-F) Quantile-quantile plots of the mean phenotype (D), the linear plasticity (E), and the non-linear plasticity (F).

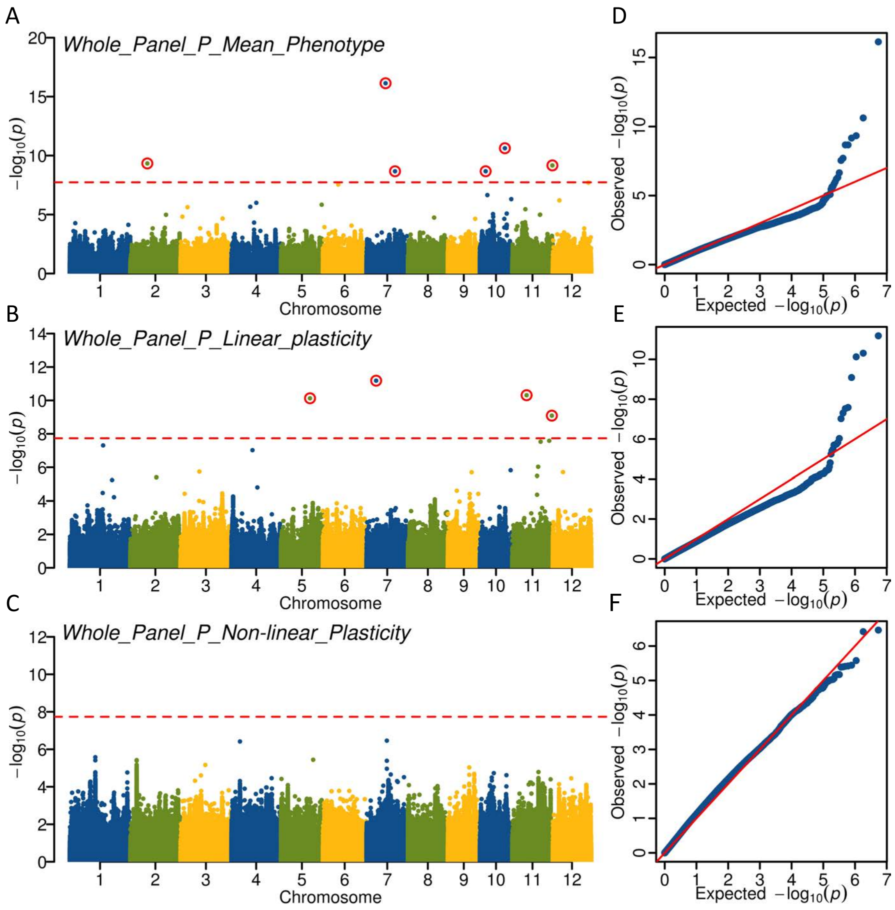

Fig. S 41 GWAS results of three phenotypic measures of P in the whole panel. (A-C) Manhattan plots of (A) the mean phenotype, (B) the linear plasticity, and (C) the non-linear plasticity, the horizontal dashed line indicates the threshold at 1.8e-8. The significantly associated loci (SALs) were labeled with red circles. (D-F) Quantile-quantile plots of the mean phenotype (D), the linear plasticity (E), and the non-linear plasticity (F).

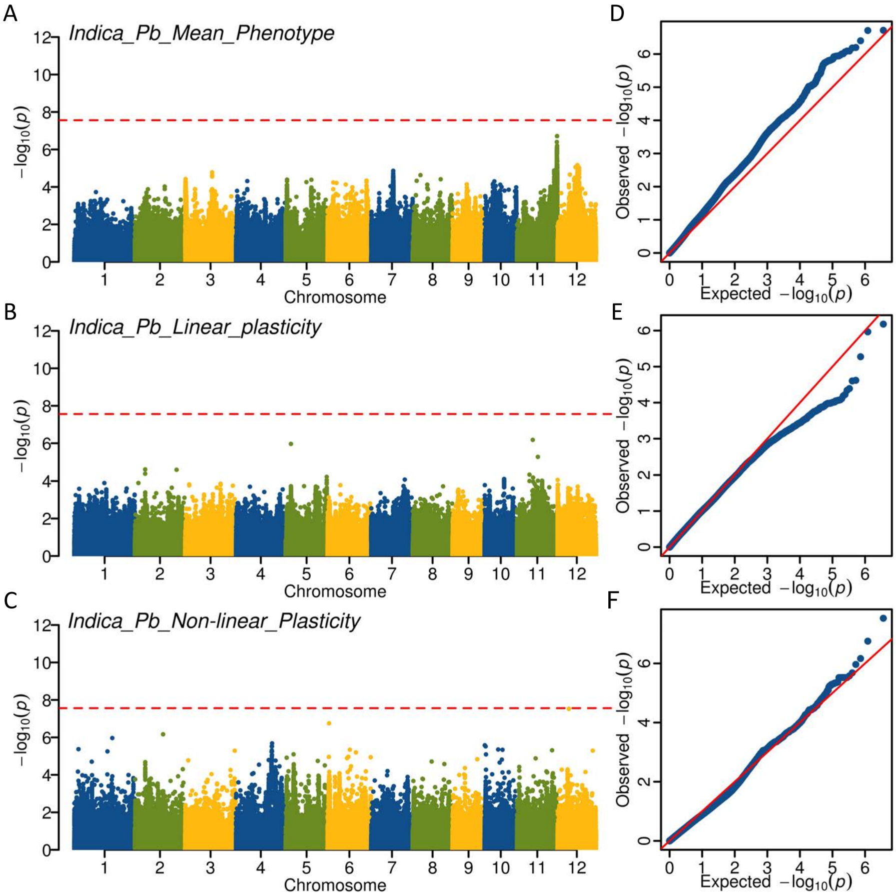

Fig. S 42 GWAS results of three phenotypic measures of Pb in the *Indica* subspecies. (A-C) Manhattan plots of (A) the mean phenotype, (B) the linear plasticity, and (C) the non-linear plasticity, the horizontal dashed line indicates the threshold at 2.7e-8. The significantly associated loci (SALs) were labeled with red circles. (D-F) Quantile-quantile plots of the mean phenotype (D), the linear plasticity (E), and the non-linear plasticity (F).

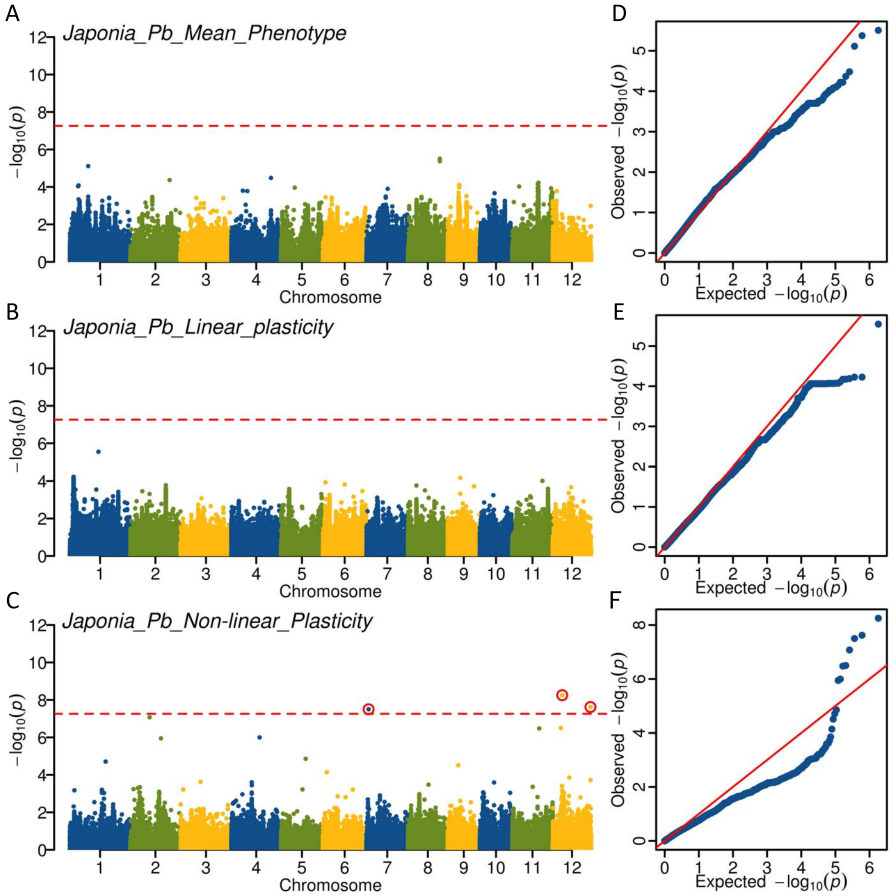

Fig. S 43 GWAS results of three phenotypic measures of Pb in the *Japonica* subspecies. (A-C) Manhattan plots of (A) the mean phenotype, (B) the linear plasticity, and (C) the non-linear plasticity, the horizontal dashed line indicates the threshold at 5.5e-8. The significantly associated loci (SALs) were labeled with red circles. (D-F) Quantile-quantile plots of the mean phenotype (D), the linear plasticity (E), and the non-linear plasticity (F).

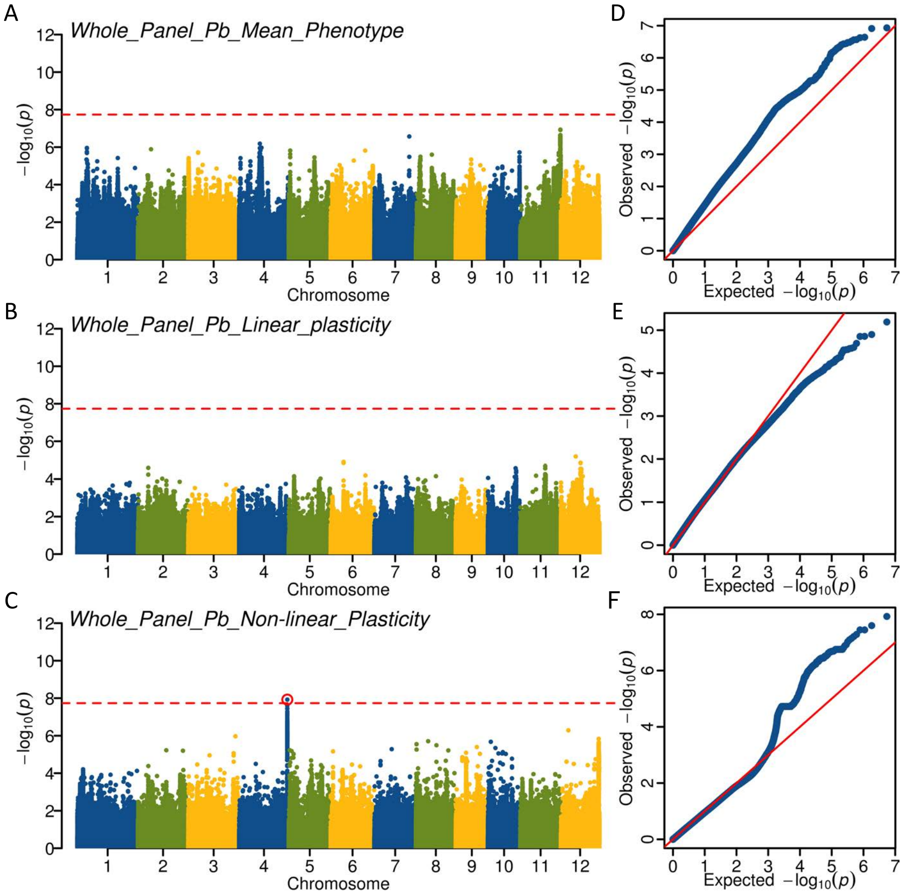

Fig. S 44 GWAS results of three phenotypic measures of Pb in the whole panel. (A-C) Manhattan plots of (A) the mean phenotype, (B) the linear plasticity, and (C) the non-linear plasticity, the horizontal dashed line indicates the threshold at 1.8e-8. The significantly associated loci (SALs) were labeled with red circles. (D-F) Quantile-quantile plots of the mean phenotype (D), the linear plasticity (E), and the non-linear plasticity (F).

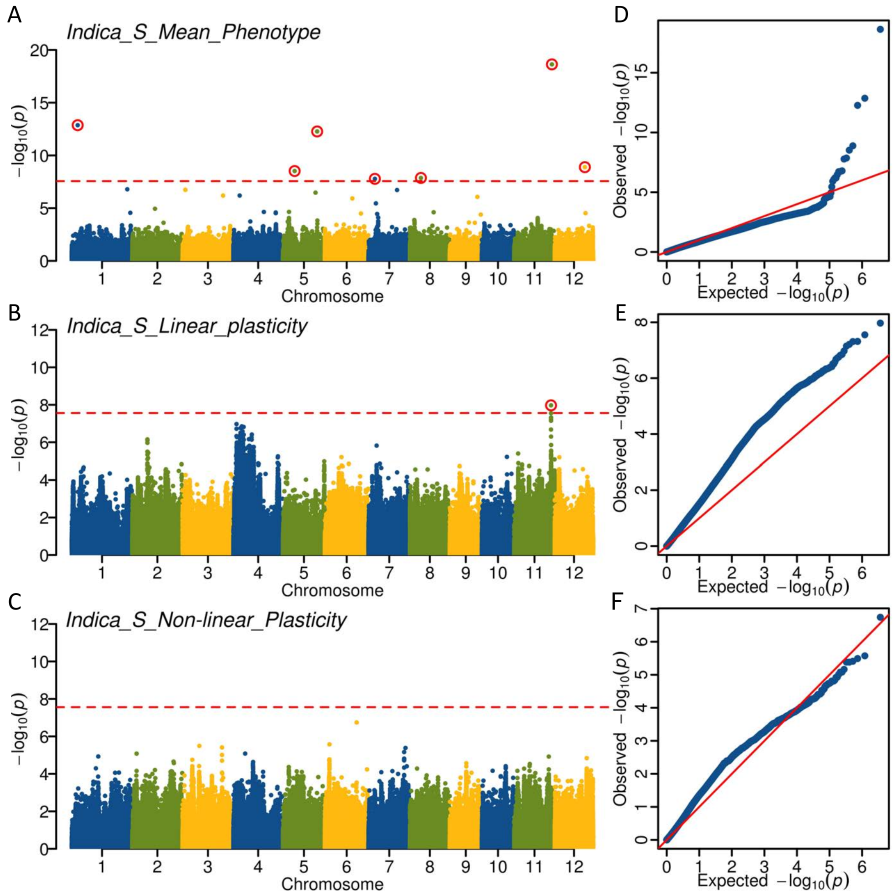

Fig. S 45 GWAS results of three phenotypic measures of S in the *Indica* subspecies. (A-C) Manhattan plots of (A) the mean phenotype, (B) the linear plasticity, and (C) the non-linear plasticity, the horizontal dashed line indicates the threshold at 2.7e-8. The significantly associated loci (SALs) were labeled with red circles. (D-F) Quantile-quantile plots of the mean phenotype (D), the linear plasticity (E), and the non-linear plasticity (F).

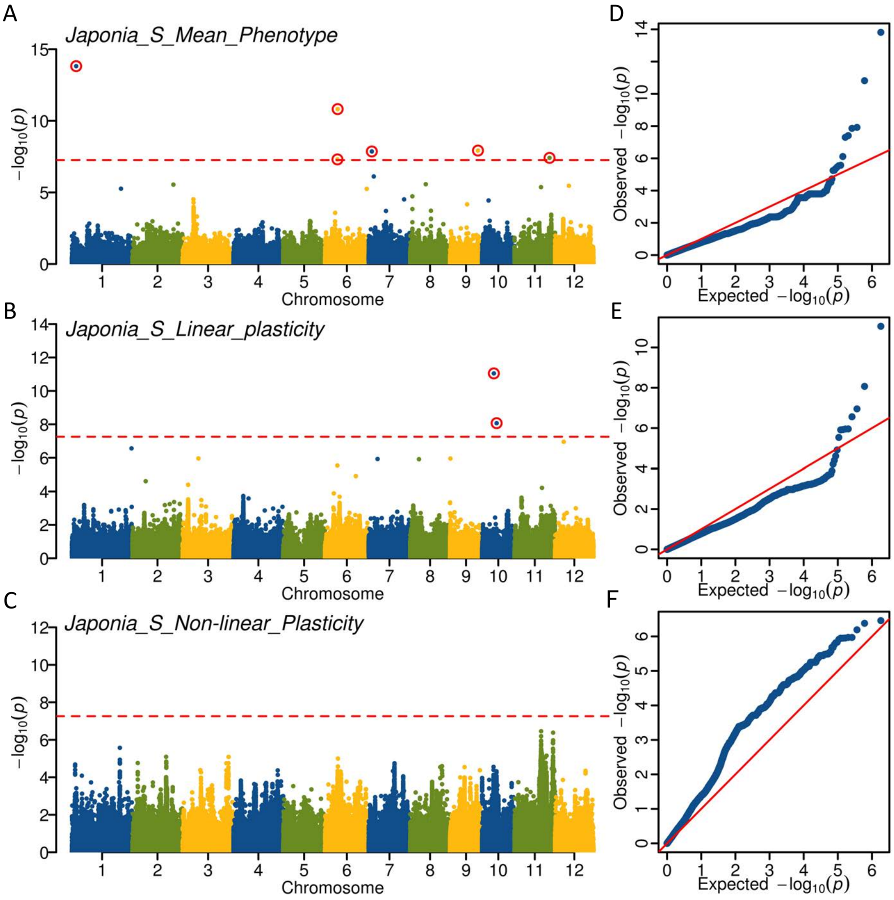

Fig. S 46 GWAS results of three phenotypic measures of S in the *Japonica* subspecies. (A-C) Manhattan plots of (A) the mean phenotype, (B) the linear plasticity, and (C) the non-linear plasticity, the horizontal dashed line indicates the threshold at 5.5e-8. The significantly associated loci (SALs) were labeled with red circles. (D-F) Quantile-quantile plots of the mean phenotype (D), the linear plasticity (E), and the non-linear plasticity (F).

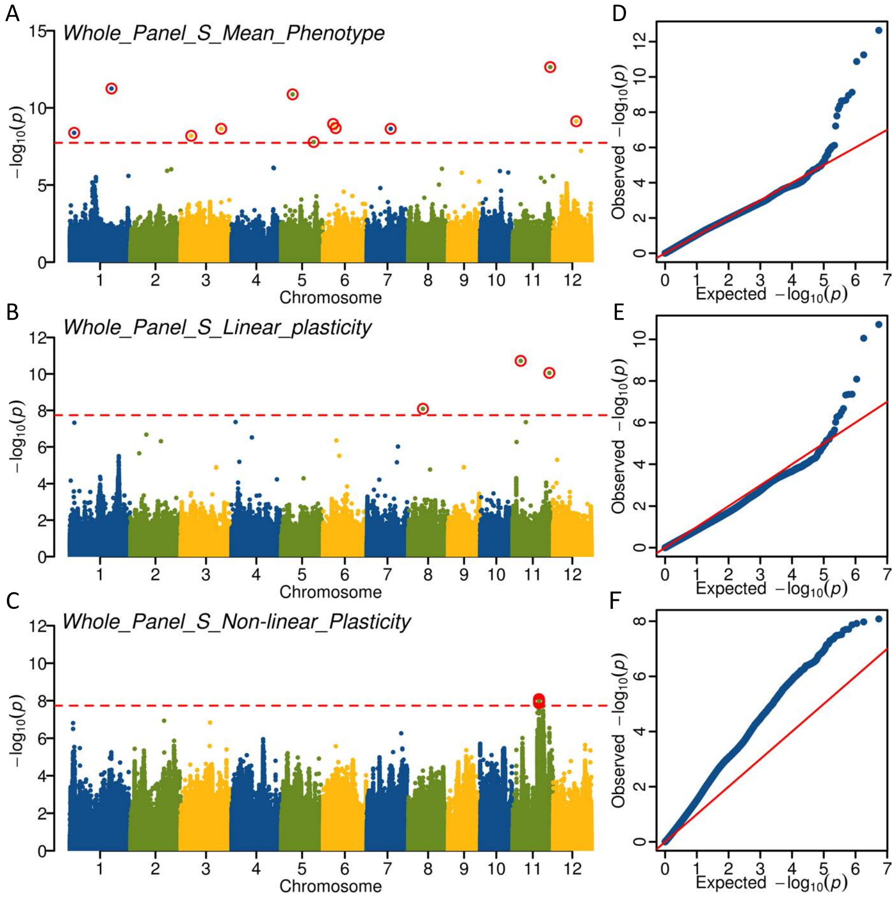

Fig. S 47 GWAS results of three phenotypic measures of S in the whole panel. (A-C) Manhattan plots of (A) the mean phenotype, (B) the linear plasticity, and (C) the non-linear plasticity, the horizontal dashed line indicates the threshold at 1.8e-8. The significantly associated loci (SALs) were labeled with red circles. (D-F) Quantile-quantile plots of the mean phenotype (D), the linear plasticity (E), and the non-linear plasticity (F).

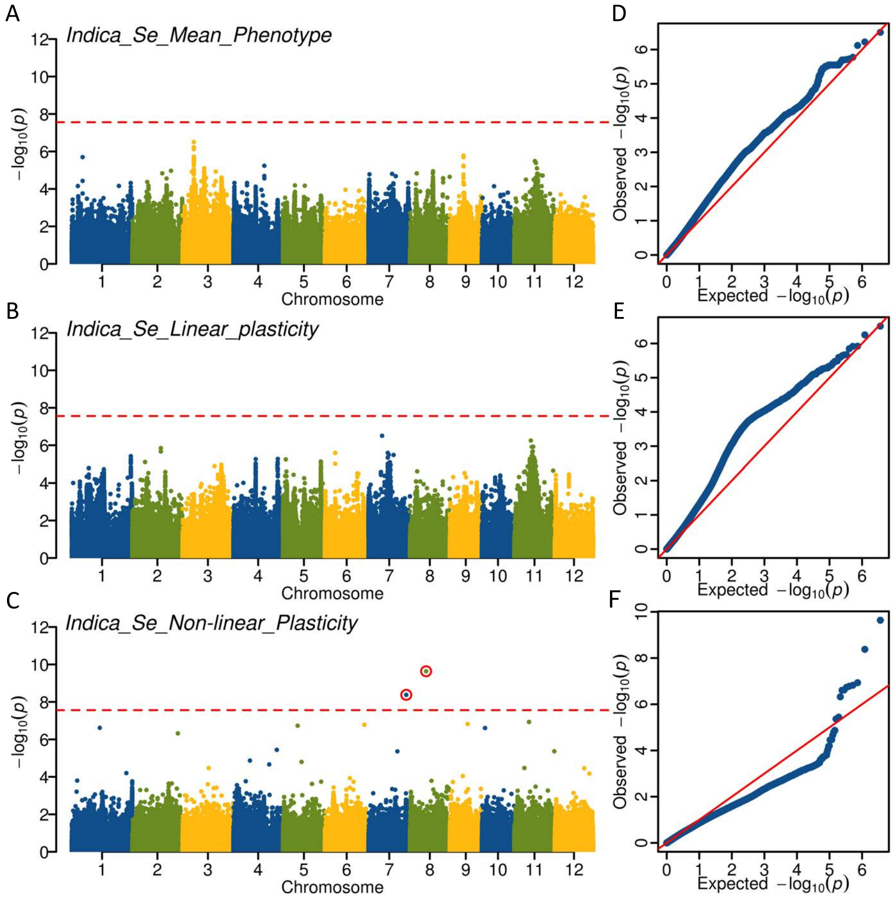

Fig. S 48 GWAS results of three phenotypic measures of Se in the *Indica* subspecies. (A-C) Manhattan plots of (A) the mean phenotype, (B) the linear plasticity, and (C) the non-linear plasticity, the horizontal dashed line indicates the threshold at 2.7e-8. The significantly associated loci (SALs) were labeled with red circles. (D-F) Quantile-quantile plots of the mean phenotype (D), the linear plasticity (E), and the non-linear plasticity (F).

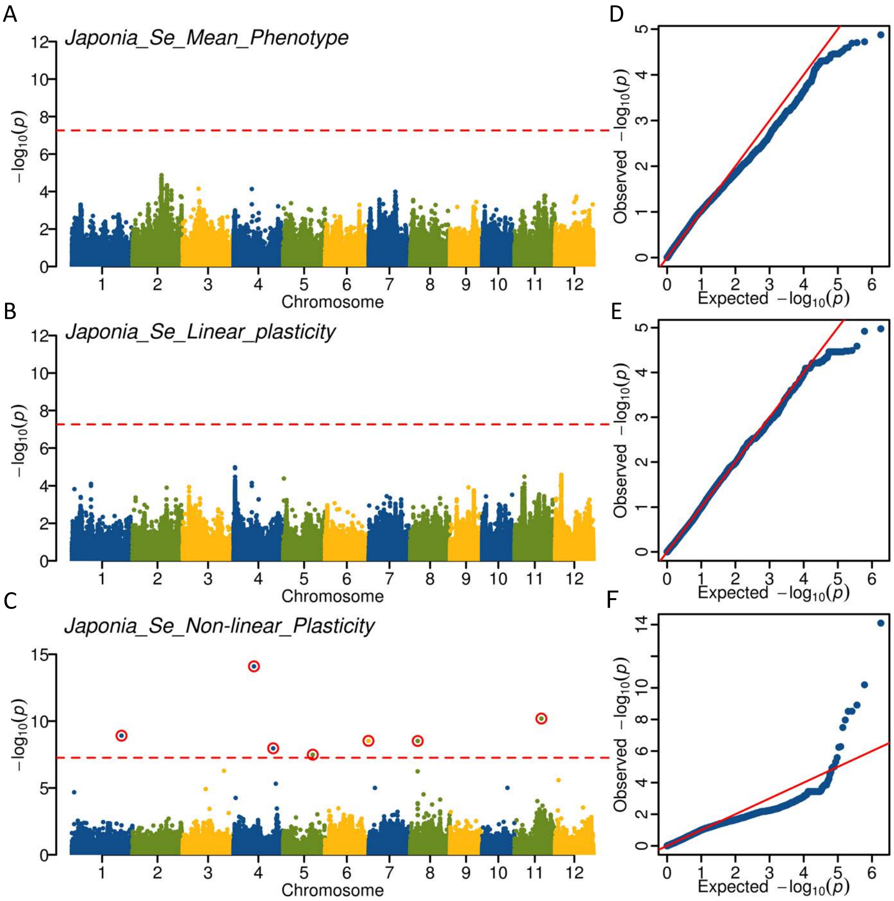

Fig. S 49 GWAS results of three phenotypic measures of Se in the *Japonica* subspecies. (A-C) Manhattan plots of (A) the mean phenotype, (B) the linear plasticity, and (C) the non-linear plasticity, the horizontal dashed line indicates the threshold at 5.5e-8. The significantly associated loci (SALs) were labeled with red circles. (D-F) Quantile-quantile plots of the mean phenotype (D), the linear plasticity (E), and the non-linear plasticity (F).

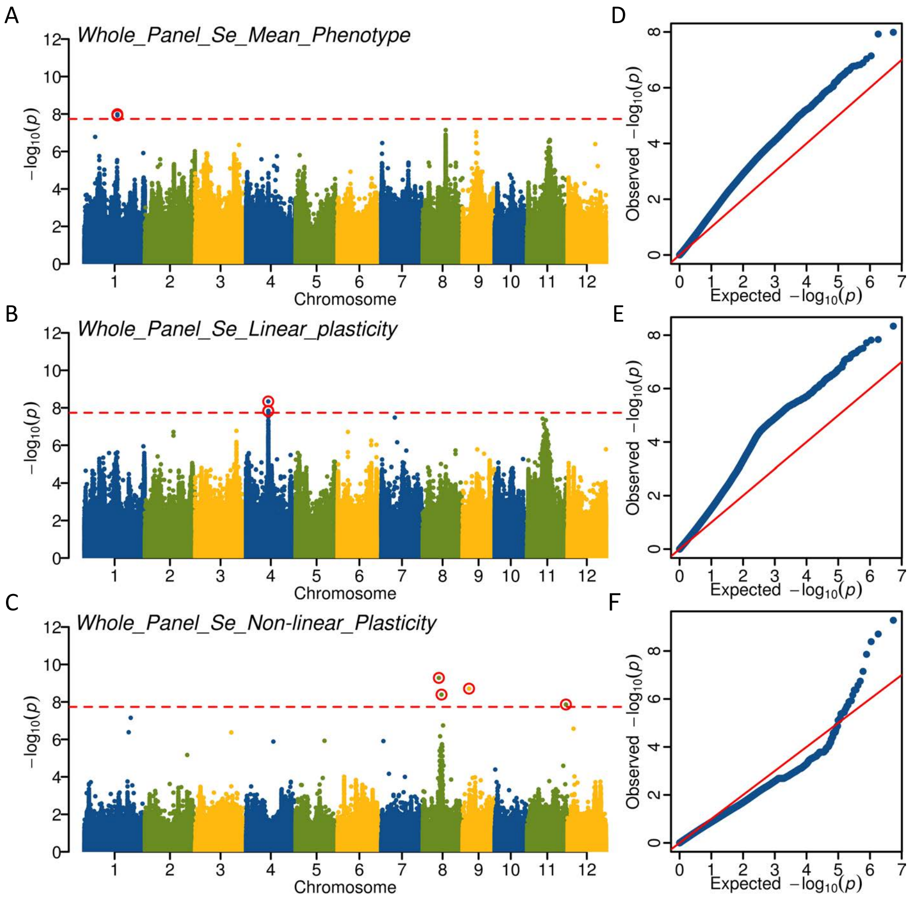

Fig. S 50 GWAS results of three phenotypic measures of Se in the whole panel. (A-C) Manhattan plots of (A) the mean phenotype, (B) the linear plasticity, and (C) the non-linear plasticity, the horizontal dashed line indicates the threshold at 1.8e-8. The significantly associated loci (SALs) were labeled with red circles. (D-F) Quantile-quantile plots of the mean phenotype (D), the linear plasticity (E), and the non-linear plasticity (F).

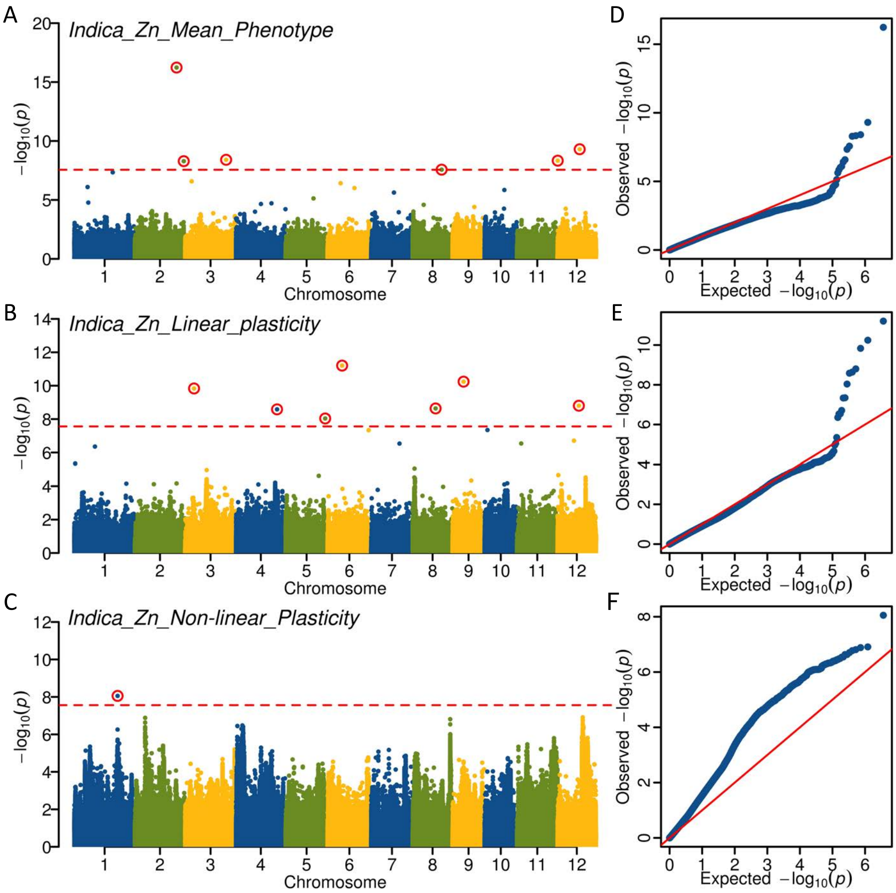

Fig. S 51 GWAS results of three phenotypic measures of Zn in the *Indica* subspecies. (A-C) Manhattan plots of (A) the mean phenotype, (B) the linear plasticity, and (C) the non-linear plasticity, the horizontal dashed line indicates the threshold at 2.7e-8. The significantly associated loci (SALs) were labeled with red circles. (D-F) Quantile-quantile plots of the mean phenotype (D), the linear plasticity (E), and the non-linear plasticity (F).

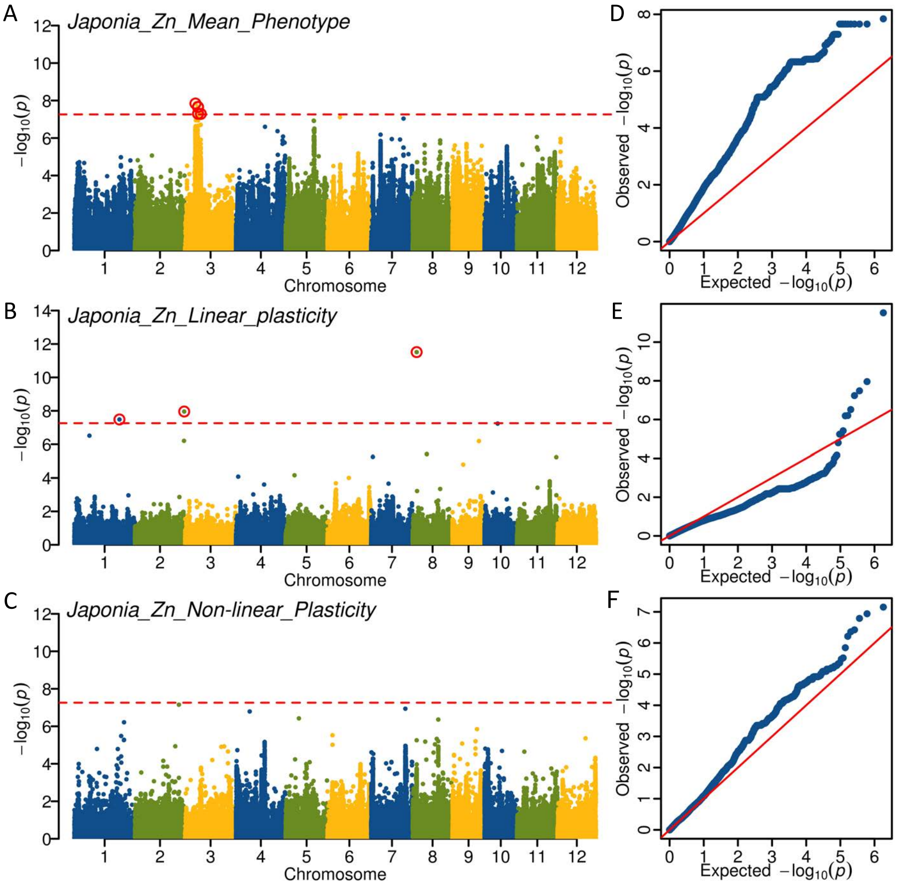

Fig. S 52 GWAS results of three phenotypic measures of Zn in the *Japonica* subspecies. (A-C) Manhattan plots of (A) the mean phenotype, (B) the linear plasticity, and (C) the non-linear plasticity, the horizontal dashed line indicates the threshold at 5.5e-8. The significantly associated loci (SALs) were labeled with red circles. (D-F) Quantile-quantile plots of the mean phenotype (D), the linear plasticity (E), and the non-linear plasticity (F).

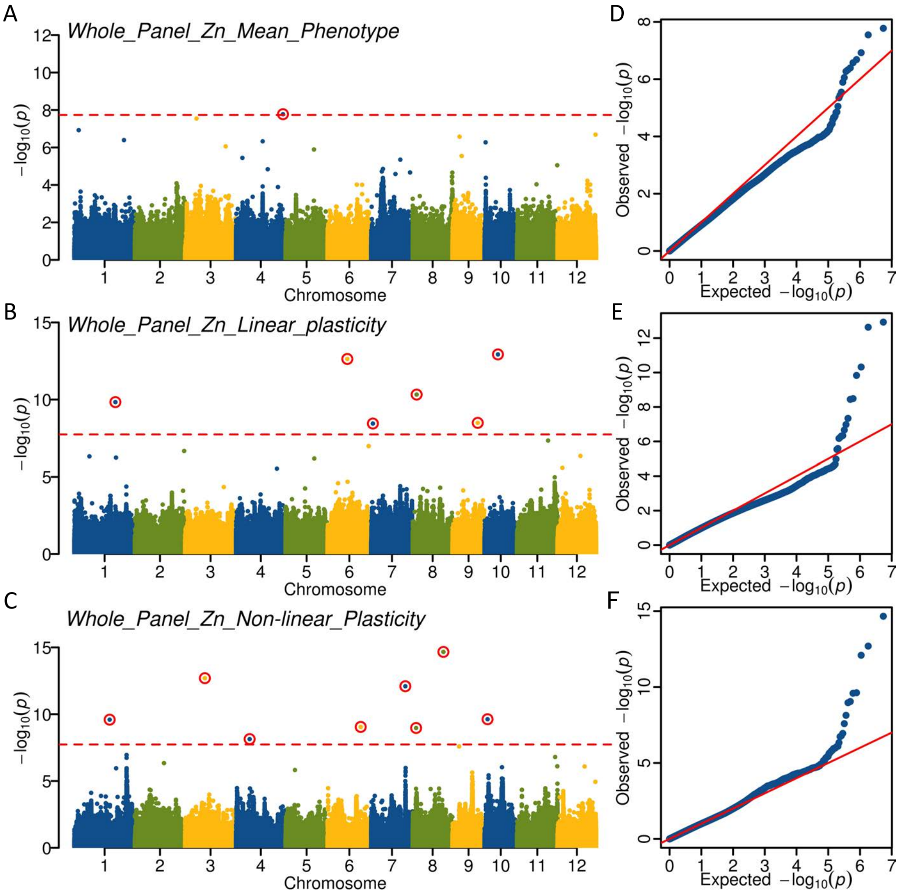

Fig. S 53 GWAS results of three phenotypic measures of Zn in the whole panel. (A-C) Manhattan plots of (A) the mean phenotype, (B) the linear plasticity, and (C) the non-linear plasticity, the horizontal dashed line indicates the threshold at 1.8e-8. The significantly associated loci (SALs) were labeled with red circles. (D-F) Quantile-quantile plots of the mean phenotype (D), the linear plasticity (E), and the non-linear plasticity (F).

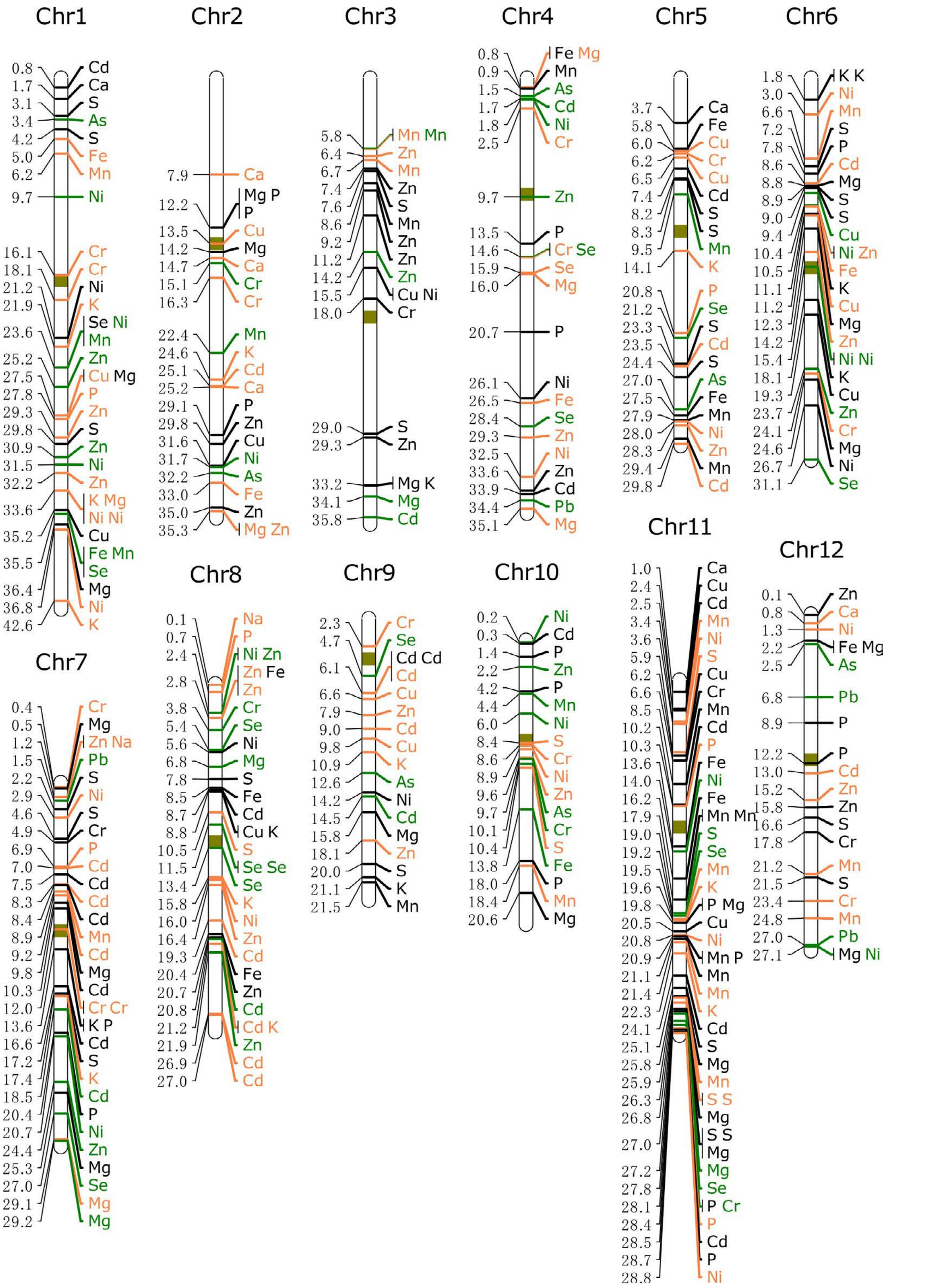

Fig. S 54 Distribution of all SALs identified in this study. Different colors were used to distinguish the mSAL (black), lSAL (green), and nlSAL (orange). The number located of the left side of each chromosome denote the physical position (Mb). The grey rectangular on each chromosome denote the centromere.

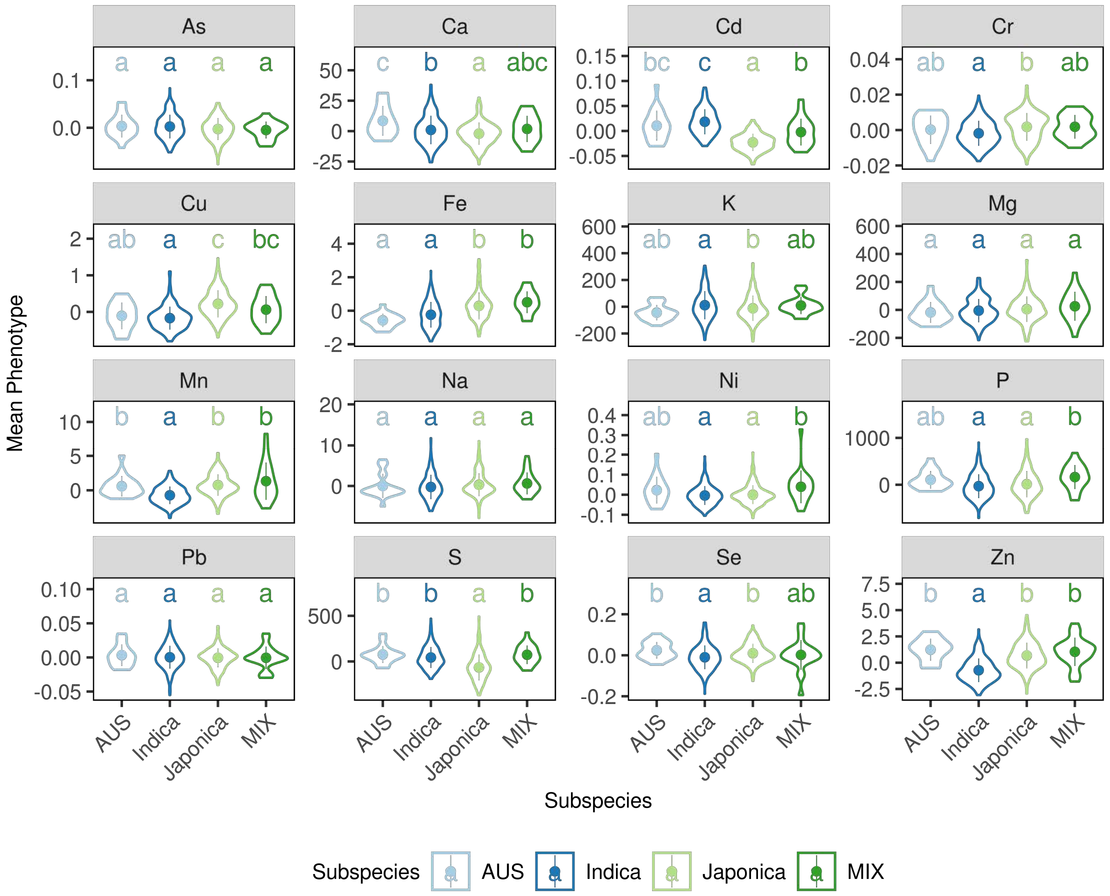

Fig. S 55 Violin plots for the mean phenotype value in each rice subspecies. The width of each violin denotes the kernel density, the point and line in each violin denotes the mean value and the standard deviation. The letters above each violin denote the significant differences between different subspecies (Kruskal-Wallis test, *P* < 0.05).

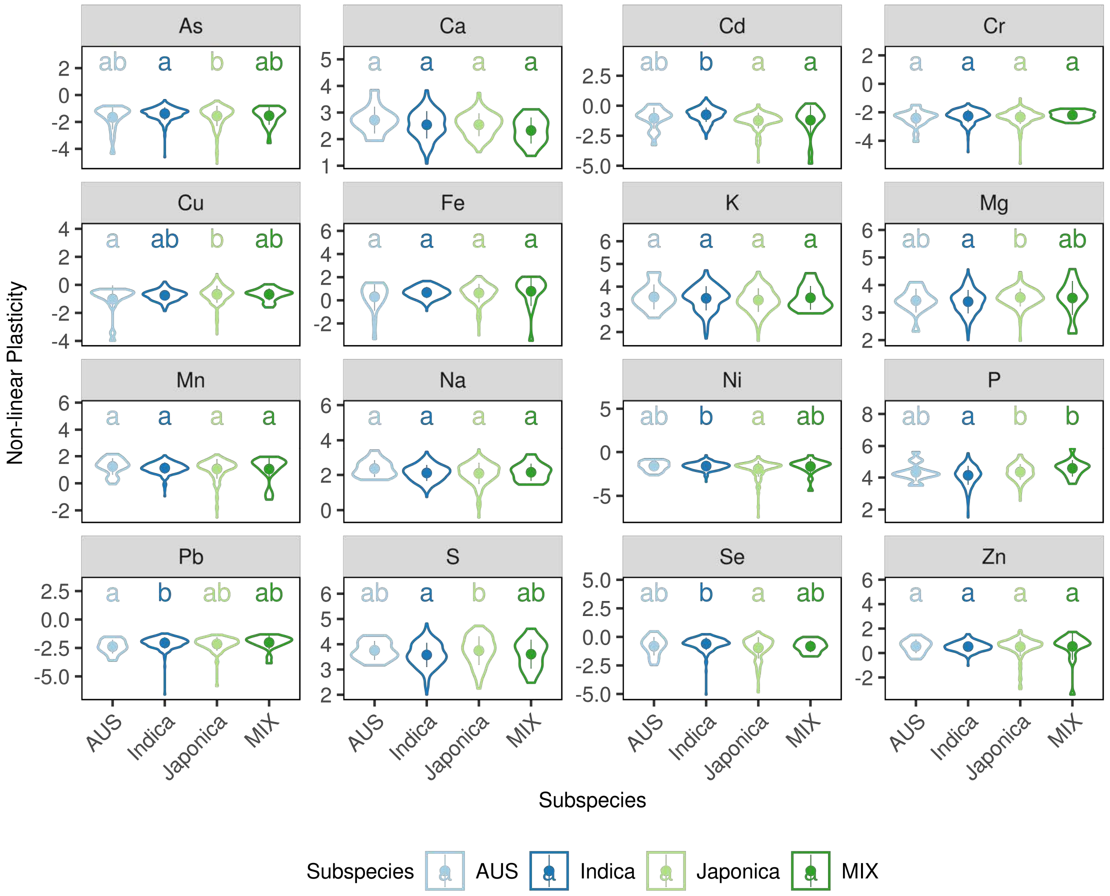

Fig. S 56 Violin plots for the non-linear plasticity in each rice subspecies. The width of each violin denotes the kernel density, the point and line in each violin denotes the mean value and the standard deviation. The letters above each violin denote the significant differences between different subspecies (Kruskal-Wallis test, *P* < 0.05).

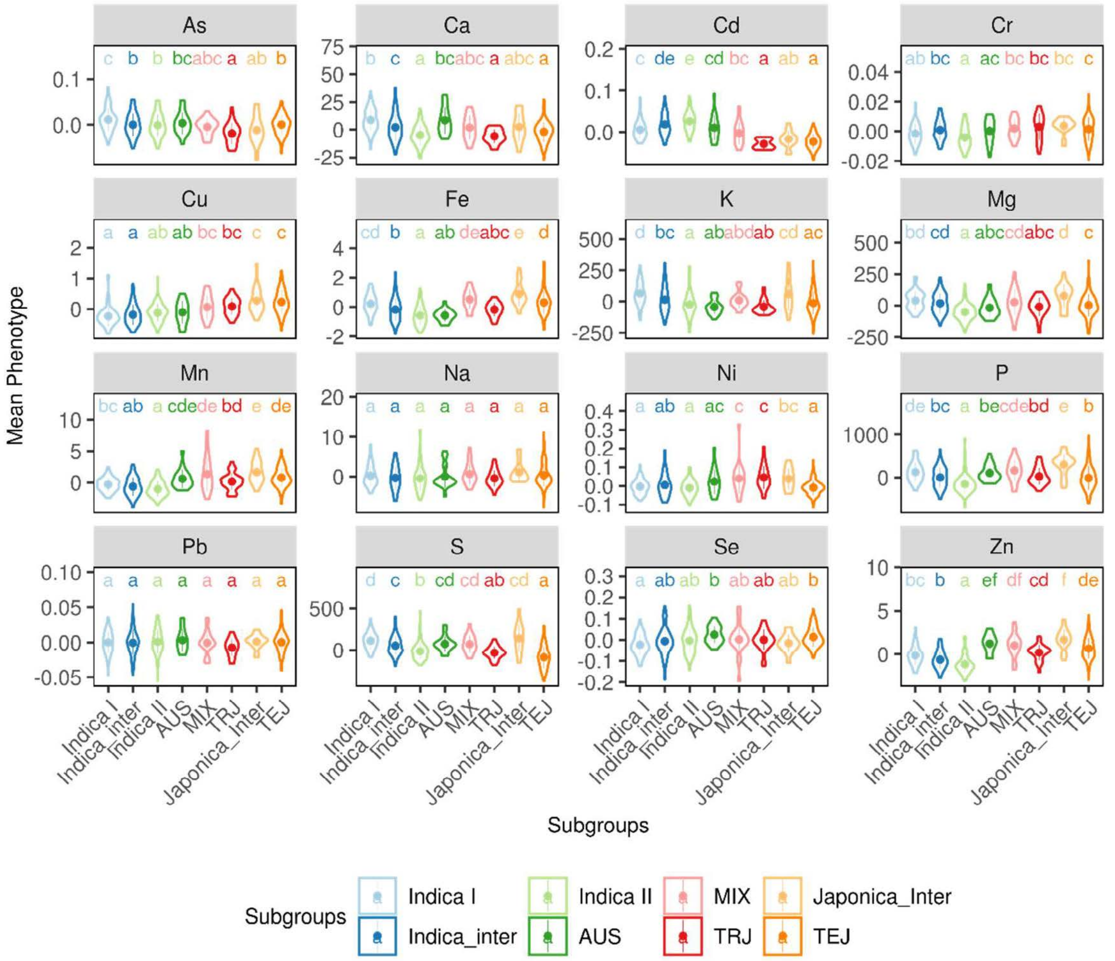

Fig. S 57 Violin plots for the mean phenotype value in each rice subgroups. The width of each violin denotes the kernel density, the point and line in each violin denotes the mean value and the standard deviation. The letters above each violin denote the significant differences between different subgroups (Kruskal-Wallis test, $P < 0.05$).

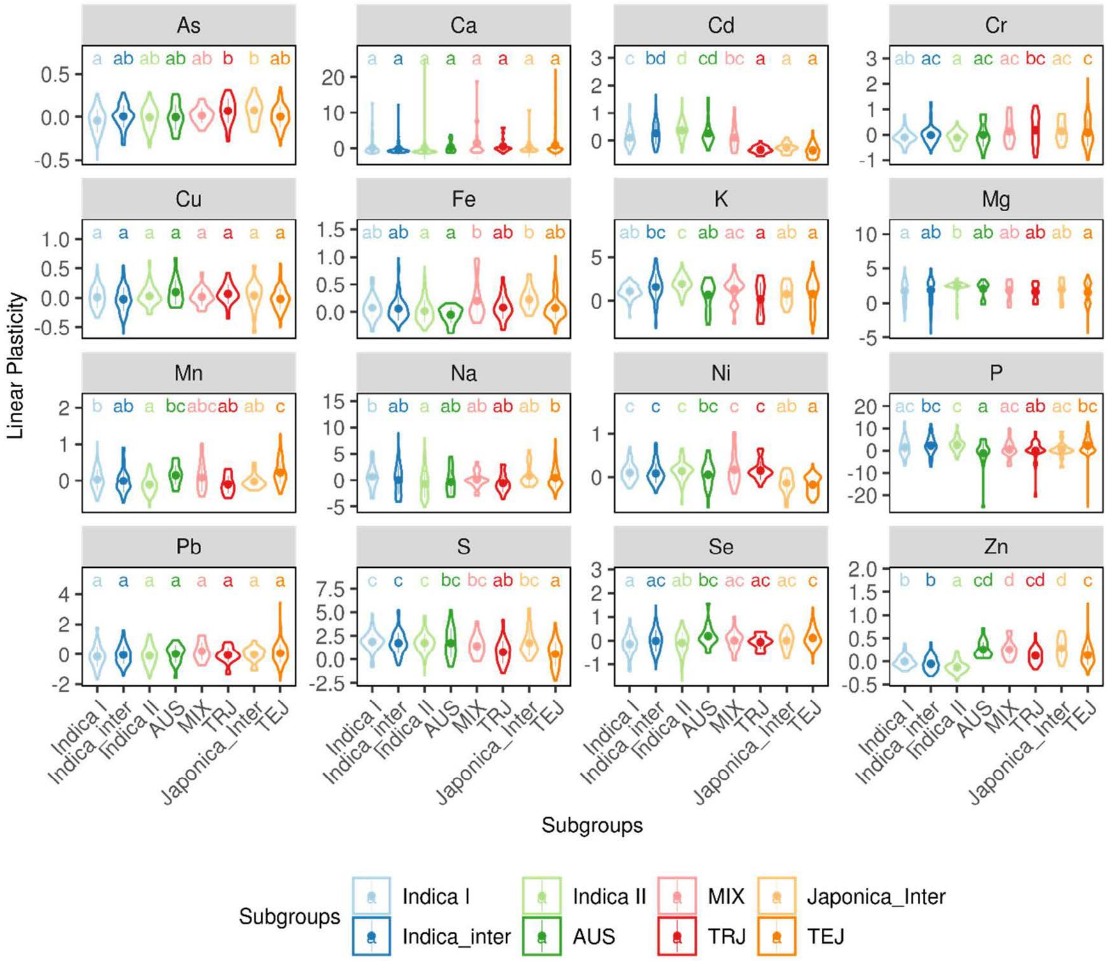

Fig. S 58 Violin plots for the linear plasticity value in each rice subgroups. The width of each violin denotes the kernel density, the point and line in each violin denotes the mean value and the standard deviation. The letters above each violin denote the significant differences between different subgroups (Kruskal-Wallis test, $P < 0.05$).

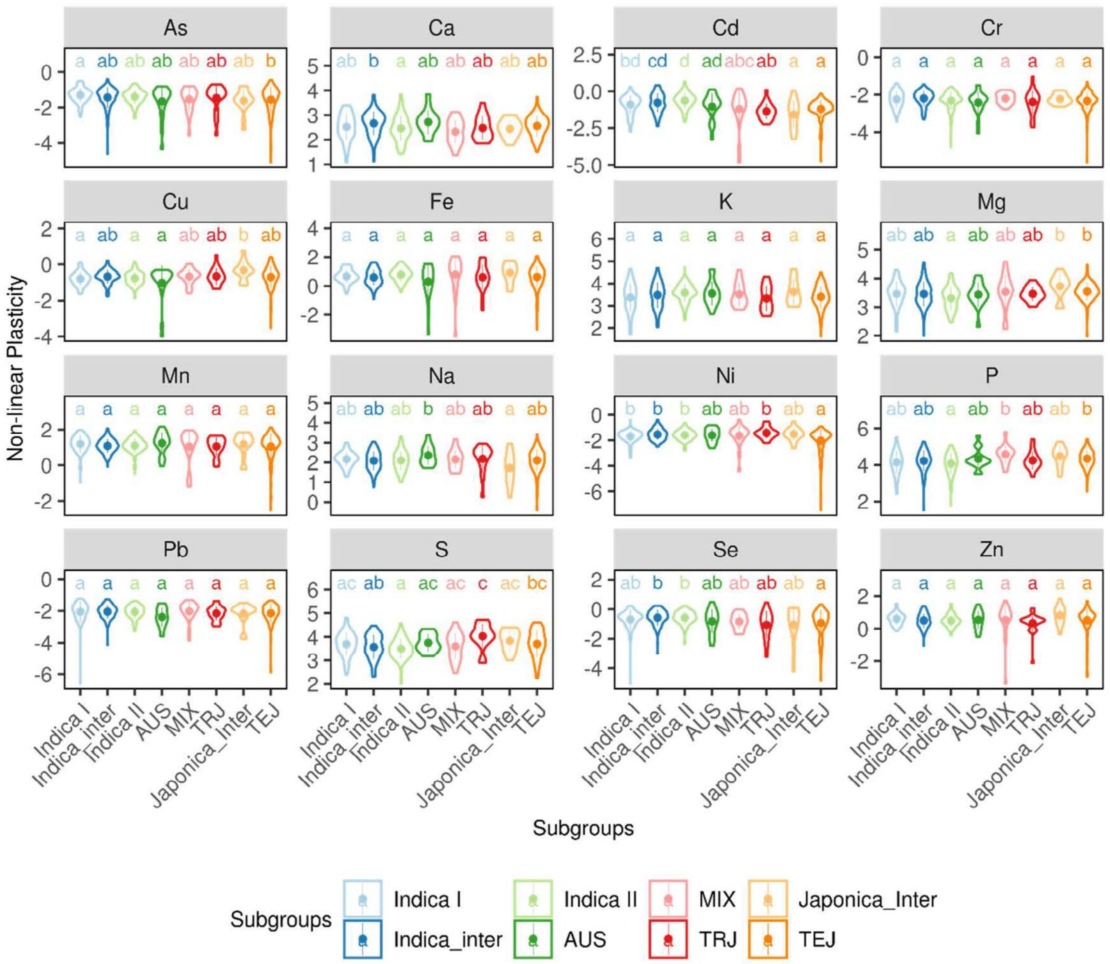

Fig. S 59 Violin plots for the non-linear plasticity value in each rice subgroups. The width of each violin denotes the kernel density, the point and line in each violin denotes the mean value and the standard deviation. The letters above each violin denote the significant differences between different subgroups (Kruskal-Wallis test, $P < 0.05$).

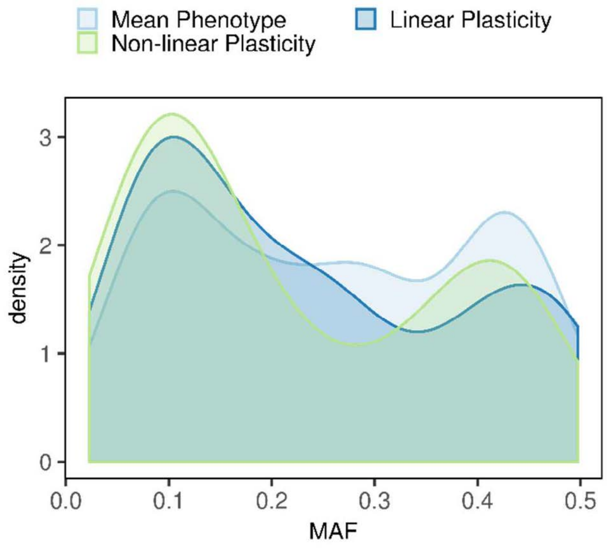

Fig. S 60 Density plot of minor allele frequency (MAF) for SALs related to three phenotypic measures (the mean phenotype, linear-plasticity, and non-linear plasticity). The y-axis denotes the kernel density of the distribution.

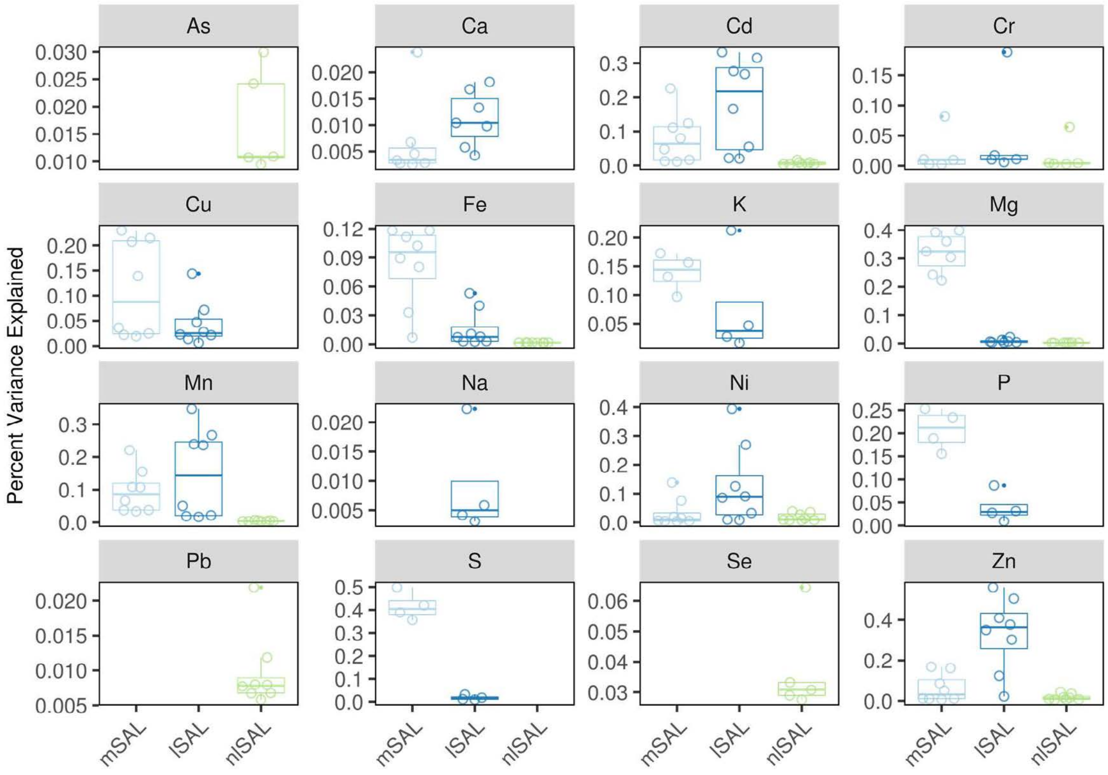

Fig. S 61 Boxplots for the percent variance explained (PVE) by three types of SALs in each environment. The PVE for SALs with a number less than two was not estimated. The blanks indicate the number of identified SALs less than two
